# Exploring the promising potential of induced pluripotent stem cells in cancer research and therapy

**DOI:** 10.1186/s12943-023-01873-0

**Published:** 2023-11-28

**Authors:** Matin Chehelgerdi, Fereshteh Behdarvand Dehkordi, Mohammad Chehelgerdi, Hamidreza Kabiri, Hosein Salehian-Dehkordi, Mohammad Abdolvand, Sharareh Salmanizadeh, Mohsen Rashidi, Anoosha Niazmand, Saba Ahmadi, Sara Feizbakhshan, Saber Kabiri, Nasimeh Vatandoost, Tayebeh Ranjbarnejad

**Affiliations:** 1 Novin Genome (NG) Lab, Research and Development Center for Biotechnology, Shahrekord, Iran; 2 Young Researchers and Elite Club, Shahrekord Branch, Islamic Azad University, Shahrekord, Iran; 3 College of Animal Science and Technology, China Agricultural University, Beijing, 100193 China; 4 Department of Genetics and Molecular Biology, School of Medicine, Isfahan University of Medical Science, Isfahan, Iran; 5 Department of Cell and Molecular Biology and Microbiology, Faculty of Biological Science and Technology, University of Isfahan, Hezar-Jereeb Street, Isfahan, 81746-73441 Iran; 6 Department Pharmacology, Faculty of Medicine, Mazandaran University of Medical Sciences, Sari, Iran; 7 The Health of Plant and Livestock Products Research Center, Mazandaran University of Medical Sciences, Sari, Iran; 8 Department of Molecular and Medical Genetics, Tbilisi State Medical University, Tbilisi, Georgia; 9 Pediatric Inherited Diseases Research Center, Research Institute for Primordial Prevention of Non-Communicable Disease, Isfahan University of Medical Sciences, Isfahan, Iran

**Keywords:** Induced pluripotent stem cells, Tumorigenesis, Therapy, Regenerative medicine, Personalized medicine, Immunotherapies

## Abstract

The advent of iPSCs has brought about a significant transformation in stem cell research, opening up promising avenues for advancing cancer treatment. The formation of cancer is a multifaceted process influenced by genetic, epigenetic, and environmental factors. iPSCs offer a distinctive platform for investigating the origin of cancer, paving the way for novel approaches to cancer treatment, drug testing, and tailored medical interventions. This review article will provide an overview of the science behind iPSCs, the current limitations and challenges in iPSC-based cancer therapy, the ethical and social implications, and the comparative analysis with other stem cell types for cancer treatment. The article will also discuss the applications of iPSCs in tumorigenesis, the future of iPSCs in tumorigenesis research, and highlight successful case studies utilizing iPSCs in tumorigenesis research. The conclusion will summarize the advancements made in iPSC-based tumorigenesis research and the importance of continued investment in iPSC research to unlock the full potential of these cells.

## Introduction

Induced pluripotent stem cells (iPSCs) are a groundbreaking discovery in the field of stem cell research [1]. iPSCs are generated by reprogramming adult cells, such as skin cells or blood cells, back into a pluripotent state, similar to embryonic stem cells [2, 3]. This reprogramming is achieved by introducing a set of specific genes into the adult cells, which reactivates their dormant pluripotent capabilities [4, 5]. Once reprogrammed, iPSCs have the remarkable ability to differentiate into any type of cell in the body, including cells of the nervous system, heart, liver, and more [6–9] This versatility makes iPSCs a powerful tool in regenerative medicine, as they hold the potential to replace damaged or diseased tissues and organs [10]. In addition to their regenerative capabilities, iPSCs have also emerged as a valuable resource in cancer research. Cancer is a complex and multifaceted disease, characterized by the uncontrolled growth and proliferation of cells [11]. It is driven by genetic mutations and alterations in the epigenetic regulation of genes [12]. iPSCs offer a unique model for studying tumorigenesis, as they can be generated from adult cells that carry specific cancer-associated mutations. By studying these iPSCs, researchers can gain insights into the molecular changes that occur during the early stages of cancer development and progression. One of the key advantages of using iPSCs in cancer research is the ability to create disease-specific cell lines [13]. By reprogramming cells from cancer patients, iPSCs can be generated that carry the same genetic mutations found in the patient’s tumor cells. These iPSC-derived cells provide an invaluable tool for studying the molecular mechanisms underlying cancer development and progression [14]. Researchers can compare these iPSC-derived cancer cells with healthy iPSC-derived cells to identify the specific genetic and epigenetic changes associated with the disease. This knowledge can then be used to develop targeted therapies that specifically address the underlying molecular defects in individual patients [15]. Furthermore, iPSCs offer a platform for drug screening and testing in the context of cancer therapy. Traditional cancer drug development often relies on animal models or immortalized cancer cell lines, which may not accurately reflect the complexity of human cancer. iPSCs can be differentiated into various cell types that represent different stages of cancer development, allowing researchers to test the efficacy and toxicity of potential drugs in a more relevant cellular context. This personalized approach to drug screening holds great promise for improving the success rate of cancer treatments and reducing the side effects associated with conventional therapies [16]. The purpose of this review article is to provide a comprehensive overview of iPSCs in the context of tumorigenesis and therapy. We will discuss the current state of iPSC research, including the challenges and limitations associated with iPSC-based cancer therapy. Ethical and social implications of iPSC research will also be explored. Additionally, we will compare iPSCs with other types of stem cells, such as embryonic stem cells (ESCs) and adult stem cells, in terms of their potential for cancer treatment. We will delve into the various applications of iPSCs in tumorigenesis research, including their use in modeling cancer development, understanding molecular changes, and facilitating early detection of cancer. The review will also highlight successful case studies where iPSCs have been utilized to gain insights into tumorigenesis and develop novel therapeutic approaches. Ultimately, we aim to emphasize the importance of continued investment in iPSC research to fully unlock the promising potential of these cells in the field of cancer research and therapy.

## The science behind iPSCs

**Fig. 1:**
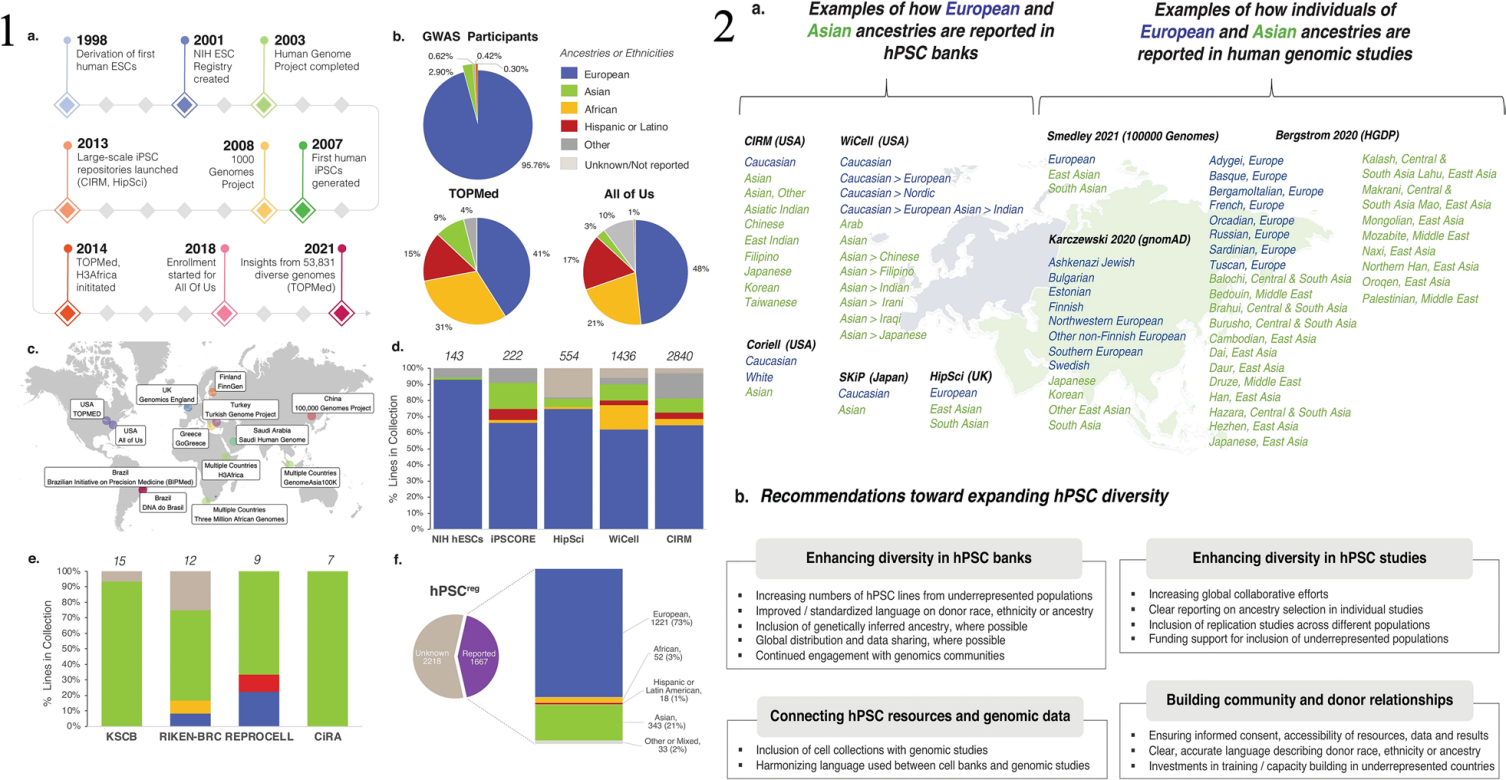
**1** The genetic diversity present in genomic research and stem cell repositories. a) Advances in human genomics and stem cell research in the past two decades have allowed for the exploration of how genetic variation influences diseases through scalable in vitro models. b) Most participants in genome-wide association studies have European ancestry. To address this limitation, initiatives such as the Trans-Omics for Precision Medicine (TOPMed) program and the All of Us Research program aim to include more diverse populations. Ancestry information or self-reported race/ethnicity data from each study is grouped into super populations. c) Various global efforts have been launched to prioritize the inclusion of underrepresented participants in human genomic research. d) The majority of pluripotent stem cell lines in large-scale collections come from donors of European ancestry. The number of cell lines in each collection is specified above each bar. The data is sourced from public repositories and peer-reviewed studies. e) Additional smaller-scale collections from different organizations and institutions are also included, such as the National Stem Cell Bank of Korea, RIKEN BRC, the CiRA Foundation, and REPROCELL. The number of cell lines from independent donors in each collection is indicated above each bar. Data from these collections is categorized into supergroups. f) The breakdown of cell lines with reported race or ethnicity data, represented as percentages within each super population, is shown using data obtained and processed with the support of the human pluripotent stem cell registry (www.hpscreg.eu). **2** Two aspects: the reporting of stem cell diversity and recommendations for expanding it. On the left side, the figure presents examples of how individuals of European and Asian ancestries are currently reported in various human pluripotent stem cell (hPSC) banks, including CIRM (USA), WiCell (USA), Coriell (USA), SKiP (Japan), and HipSci (UK). The colors blue and green represent individuals of European and Asian ancestries, respectively. On the right side, the figure shows examples of how individuals of European and Asian ancestries are reported in human genomic studies. Specifically mentioned studies are Bergstrom et al. 2020 (Human Genome Diversity Project), Karczewski et al. 2020 (gnomAD), and Smedley et al. 2021 (100,000 Genomes Pilot). In panel b, the figure provides key recommendations aimed at expanding hPSC diversity. Unfortunately, the details of these recommendations are not mentioned in the description. The map used in the figure is adapted from Templates by Yourfreetemplates.com. Reprinted from [17] with permission from the Springer Nature

**Fig. 2:**
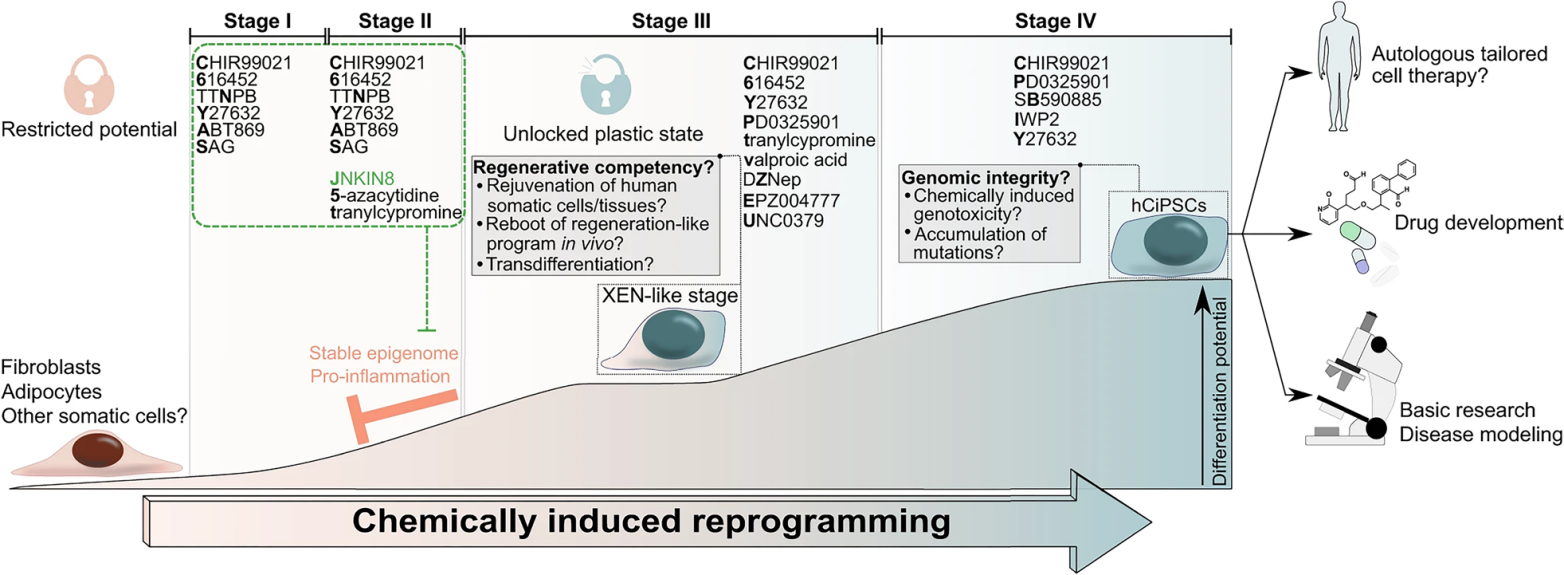
The process of chemically induced reprogramming of human somatic cells into human chemically induced pluripotent stem cells (hCiPSC). The researchers, Guan et al., have developed a well-defined reprogramming protocol consisting of four stages (stage I to stage IV) that utilizes only small molecules. By disrupting the identity and modifying the epigenetics of the somatic cells, the cells are transformed into a flexible XEN-like state with unlocked potential. To facilitate this dedifferentiation and enhance cell plasticity, it was crucial to downregulate pro-inflammatory pathways, which was achieved with the c-Jun N-terminal kinase inhibitor (JNKIN8). The acquisition of cell plasticity in the XEN-like stage enables further reprogramming into stable hCiPSCs. These hCiPSCs have various applications in basic research, such as investigating reprogramming pathways or screening for druggable targets that determine cell fate, which could lead to new therapeutic options. Additionally, the reprogramming process is compliant with Good Manufacturing Practice (GMP) standards and cost-effective, which makes it more feasible to translate iPSCs into personalized autologous cell therapies. Reprinted from [21] with permission from the Springer Nature

Ghosh, Nehme, and Barrett emphasize the critical need for greater genetic diversity within human pluripotent stem cell models. Despite the expansion of repositories and studies in this field, a noticeable lack of genetic diversity persists. The authors underscore the significance of including diverse ancestral backgrounds in these models, highlighting that such inclusion is essential not only for promoting equity but also for expediting advancements in biological research and discovery [17]. The study, depicted in Fig. 1, analyzed the current landscape of genetic diversity in hPSC banks and human genomic studies. The findings revealed a significant underrepresentation of individuals with non-European and non-Asian ancestries in both repositories. This lack of diversity limits the generalizability of research findings and hinders our understanding of the impact of genetic variation on disease and treatment outcomes. The study emphasizes the importance of incorporating iPSCs into research initiatives, as illustrated in Fig. 1. By collecting material for iPSC reprogramming alongside genomic and phenotypic data, a direct link between genetic information and cellular resources can be established. This approach ensures that the genetic diversity of hPSC models is enhanced, enabling more accurate disease modeling and personalized medicine approaches. The iPSCs have emerged as a groundbreaking technology in the field of stem cell research [18] are generated by reprogramming adult somatic cells, such as skin cells or blood cells, into a pluripotent state similar to ESCs [19]. This reprogramming is achieved by introducing a set of defined transcription factors, known as the Yamanaka factors, into the adult cells. The Yamanaka factors include Oct3/4, Sox2, Klf4, and c-Myc, which are capable of reprogramming the cells’ gene expression patterns, allowing them to regain pluripotency [20]. The first generation of iPSCs emerged when Dr. Shinya Yamanaka and his team introduced the groundbreaking 4-factor protocol. This protocol involved the introduction of four key transcription factors—Oct4, Sox2, Klf4, and c-Myc—into somatic cells, effectively reprogramming them into iPSCs. Yamanaka’s discovery represented a pivotal moment in regenerative medicine, as it provided a relatively simple and reproducible method for generating iPSCs. These cells possessed the ability to differentiate into various cell types, making them invaluable for disease modeling, drug screening, and potential therapeutic applications [20]. Subsequent generations of iPSC reprogramming protocols aimed to improve safety and efficiency. The second generation involved the replacement of c-Myc, a potentially oncogenic factor, with alternative genes, such as Nanog or Lin28. This modification reduced the risk of tumorigenicity associated with c-Myc. Third-generation protocols focused on enhancing the efficiency of reprogramming, often incorporating small molecules and microRNAs to accelerate the process and improve the quality of iPSCs generated. These advancements brought iPSC technology closer to clinical applications by minimizing genetic abnormalities and increasing the yield of pluripotent cells [18]. The Yamanaka 4-factor protocol stands as a landmark achievement that laid the foundation for iPSC research, enabling scientists to harness the potential of these cells for various biomedical applications. As iPSC technology continues to evolve, it holds immense promise for personalized medicine, disease modeling, and regenerative therapies, offering hope for a future where patient-specific treatments are commonplace [19]. In a recent study, some researchers provided a groundbreaking approach to reprogram human somatic cells into chemically induced pluripotent stem cells (CiPSCs) was presented [21]. The study aimed to address the safety concerns associated with traditional methods of reprogramming, such as the use of viral vectors or ectopic expression of potential oncogenes. The researchers developed a fully defined and precisely staged chemically induced reprogramming protocol using small molecules. Lange et al. illustrates the process of chemically induced reprogramming, highlighting the key steps involved in transforming human somatic cells into human CiPSCs (Fig. 2). This method offers a potential solution to the challenges faced in clinical translation of iPSCs and opens up new possibilities for regenerative medicine. By generating pluripotent cells through a chemically induced approach, researchers can overcome ethical concerns and create personalized therapies for a wide range of diseases and conditions. The ability to generate iPSCs has revolutionized the field of regenerative medicine and opened up new possibilities for disease modeling, drug discovery, and personalized medicine [22]. iPSCs have the remarkable potential to differentiate into any cell type in the human body, making them an invaluable tool for studying human development, disease mechanisms, and therapeutic interventions [23]. One of the most significant advantages of iPSCs is their capacity to serve as a model for tumorigenesis [24]. Cancer is a complex disease characterized by the accumulation of genetic and epigenetic alterations that disrupt normal cellular processes. iPSCs can be reprogrammed from patient-derived cancer cells, allowing researchers to investigate the molecular changes and genetic abnormalities associated with cancer development [14]. By studying iPSCs derived from cancer patients, scientists can gain insights into the early events that initiate cancer and the subsequent processes that drive its progression [11]. Additionally, iPSCs offer a platform for drug screening and the development of personalized cancer therapies [25]. Patient-specific iPSCs can be differentiated into various cell types, including cancer cells, which can be used to test the efficacy and toxicity of different drugs [14]. This approach allows for the identification of personalized treatment options based on the specific genetic and molecular characteristics of an individual’s cancer [26]. By tailoring therapies to a patient’s unique genomic profile, iPSC-based approaches hold great promise for improving treatment outcomes and minimizing adverse effects. Despite their tremendous potential, there are several challenges associated with iPSC-based cancer therapy [27]. One of the major concerns is the tumorigenic properties of iPSCs themselves. iPSCs have the capacity to form tumors called teratomas when injected into living organisms. Teratomas consist of a mixture of different cell types derived from the three germ layers, highlighting the pluripotent nature of iPSCs [28]. To overcome this obstacle, researchers are actively exploring methods to improve the differentiation efficiency of iPSCs, ensuring that they fully mature into the desired cell type before transplantation. Another challenge is the efficient and safe delivery of iPSC-derived therapeutic cells to the tumor site [29]. Effective targeting and integration of iPSC-derived cells into the tumor microenvironment are critical for successful treatment outcomes [29]. Additionally, the potential immunogenicity of iPSCs and the risk of immune rejection need to be carefully considered when developing iPSC-based cancer therapies [30]. Ethical and legal considerations also come into play when working with iPSCs. The generation of iPSCs involves the use of human embryos or the reprogramming of adult cells, which raises ethical concerns and regulatory issues [31]. It is important to ensure that iPSC research is conducted ethically, with proper informed consent and adherence to established guidelines [32]. Comparative analysis with other stem cell types is crucial for evaluating the potential of iPSCs in cancer treatment (Table 1). While ESCs are considered the gold standard for pluripotent stem cells, their use is limited due to ethical concerns and immune rejection risks [33]. Adult stem cells, such as mesenchymal stem cells (MSCs), offer advantages in terms of immune compatibility but have limited differentiation potential [34]. iPSCs bridge this gap by providing a virtually unlimited source of patient-specific pluripotent stem cells with reduced immune rejection risks. In recent years, significant progress has been made in harnessing the potential of iPSCs for tumorigenesis research [22, 23]. Several successful case studies have demonstrated the utility of iPSCs in understanding the molecular mechanisms underlying cancer development and progression [35]. For instance, iPSCs derived from patients with specific types of cancer, such as leukemia or breast cancer, have been used to recapitulate the disease phenotype in a laboratory setting [15]. By studying these iPSC-derived cancer cells, researchers have gained valuable insights into the genetic and epigenetic alterations that contribute to tumor formation and progression. Moreover, iPSCs have been employed in cancer early detection strategies [36]. The ability to reprogram cancer cells into iPSCs allows for the identification of early molecular changes that occur during tumorigenesis [37]. By comparing iPSCs derived from healthy individuals with those derived from cancer patients, researchers can identify specific biomarkers or gene expression patterns that indicate the presence of cancer [38]. This knowledge could potentially revolutionize cancer diagnostics, enabling early detection and intervention when the disease is most treatable. In addition to cancer modeling and early detection, iPSCs hold promise in the development of precision medicine approaches for cancer treatment [36]. By generating iPSCs from patients with different types of cancer, researchers can create a diverse library of cancer cell lines that reflect the heterogeneity of the disease. Table 2 provides an overview of different iPSC lines. This resource can be used to test the effectiveness of various treatment options on individualized iPSC-derived cancer cells, allowing for the identification of targeted therapies tailored to a patient’s unique genetic profile. iPSC-based precision medicine has the potential to enhance treatment outcomes by improving the specificity and efficacy of cancer therapies while minimizing unnecessary side effects [26]. Looking ahead, the future of iPSCs in tumorigenesis research is filled with exciting possibilities. Advances in gene editing technologies, such as CRISPR-Cas9, combined with iPSCs, offer unprecedented opportunities for understanding the functional consequences of specific genetic alterations in cancer [39]. By precisely modifying the genome of iPSCs, researchers can investigate the effects of specific mutations or gene dysregulation on cancer development and progression. This knowledge can inform the development of targeted therapies that directly address the underlying genetic drivers of cancer [40]. Furthermore, ongoing efforts to improve the safety and efficiency of iPSC generation and differentiation techniques are crucial for their successful translation into clinical applications. Researchers are exploring novel reprogramming methods, such as the use of non-integrating viral vectors or small molecules, to enhance the efficiency and safety of iPSC generation [41]. Additionally, strategies to enhance the differentiation of iPSCs into specific cell types relevant to cancer therapy are being investigated, including the development of defined culture conditions and the use of signaling molecules or growth [29].

**Table 1:** Comparison of iPSCs with other stem cell types for cancer treatment

Stem Cell Types	Characteristics	Advantages	Limitations	Clinical Trials	Future Directions	References
iPSCs	Origin: Induced pluripotent stem cells derived from adult cells	Pluripotency: Can differentiate into various cell types	Differentiation potential: Can be directed towards specific lineages	Immunogenicity: May trigger immune response	Advantages: Easy access to patient-specific cells, potential for personalized therapy	[41]
ESCs	Origin: Derived from embryos	Pluripotency: Can differentiate into various cell types	Differentiation potential: High potential for differentiation	Immunogenicity: May trigger immune response	Advantages: Robust differentiation capacity, well-established research tool	[38]
Adult stem cells	Origin: Found in various tissues and organs	Pluripotency: Limited differentiation potential	Differentiation potential: Primarily restricted to tissue-specific lineages	Immunogenicity: Low immunogenicity	Advantages: Easy access, reduced risk of tumor formation	[15]
Cancer stem cells	Origin: Subset of cells within tumors	Pluripotency: Capable of self-renewal and differentiation	Differentiation potential: Tumor-specific cell types	Immunogenicity: May evade immune response	Advantages: Targeting tumor-specific cells, potential for eradicating cancer-initiating cells	[32]
Mesenchymal stem cells (MSCs)	Origin: Found in various tissues (e.g., bone marrow, umbilical cord)	Pluripotency: Limited differentiation potential	Differentiation potential: Can differentiate into mesodermal lineages	Immunogenicity: Low immunogenicity	Advantages: Immunomodulatory properties, potential for tissue repair	[42]
Neural stem cells	Origin: Found in the central nervous system	Pluripotency: Limited differentiation potential	Differentiation potential: Primarily neural lineages	Immunogenicity: Variable immunogenicity	Advantages: Ability to target brain tumors, potential for neural repair	[43]
Hematopoietic stem cells (HSCs)	Origin: Found in bone marrow and peripheral blood	Pluripotency: Limited differentiation potential	Differentiation potential: Can differentiate into various blood cell types	Immunogenicity: Low immunogenicity	Advantages: Well-established source of stem cells for clinical use, potential for reconstitution of the immune system	[44]
Induced neural stem cells (iNSCs)	Origin: Induced from fibroblasts using transcription factors	Pluripotency: Limited differentiation potential	Differentiation potential: Primarily neural lineages	Immunogenicity: Variable immunogenicity	Advantages: Easy access to patient-specific cells, potential for neural repair	[45]
Pluripotent stem cells (PSCs)	Origin: Can be derived from embryos or reprogrammed from adult cells	Pluripotency: Can differentiate into various cell types	Differentiation potential: Can be directed towards specific lineages	Immunogenicity: May trigger immune response	Advantages: Wide range of potential applications, robust differentiation capacity	[46]
Dental pulp stem cells (DPSCs)	Origin: Found in dental pulp	Pluripotency: Limited differentiation potential	Differentiation potential: Primarily mesenchymal lineages	Immunogenicity: Low immunogenicity	Advantages: Easy access to source tissue, potential for tissue repair	[47]
Endothelial progenitor cells (EPCs)	Origin: Found in bone marrow and peripheral blood	Pluripotency: Limited differentiation potential	Differentiation potential: Primarily endothelial lineages	Immunogenicity: Low immunogenicity	Advantages: Potential for vascular repair and regeneration, low risk of tumor formation	[48]
Adipose-derived stem cells (ADSCs)	Origin: Found in adipose tissue	Pluripotency: Limited differentiation potential	Differentiation potential: Primarily mesenchymal lineages	Immunogenicity: Low immunogenicity	Advantages: Abundant source, easy access, potential for tissue repair	[49]
Induced hepatic progenitor cells (iHepPCs)	Origin: Induced from fibroblasts or other somatic cells	Pluripotency: Limited differentiation potential	Differentiation potential: Primarily hepatic lineages	Immunogenicity: Variable immunogenicity	Advantages: Potential for liver regeneration and transplantation	[50]
Amniotic fluid stem cells (AFSCs)	Origin: Found in amniotic fluid	Pluripotency: Limited differentiation potential	Differentiation potential: Primarily mesenchymal lineages	Immunogenicity: Low immunogenicity	Advantages: Non-invasive collection, low ethical concerns, potential for tissue repair	[51]
Skeletal muscle-derived stem cells (Sk-MSCs)	Origin: Found in skeletal muscle tissue	Pluripotency: Limited differentiation potential	Differentiation potential: Primarily mesenchymal lineages	Immunogenicity: Low immunogenicity	Advantages: Easy access to tissue source, potential for muscle repair and regeneration	[52]
Wharton's jelly-derived mesenchymal stem cells (WJ-MSCs)	Origin: Found in Wharton's jelly of the umbilical cord	Pluripotency: Limited differentiation potential	Differentiation potential: Primarily mesenchymal lineages	Immunogenicity: Low immunogenicity	Advantages: Non-invasive collection, abundant source, potential for tissue repair	[53]

**Table 2:** An overview of different types of iPSCs and their characteristics

iPSC Line	Origin of cells used for iPSC generation	Method of iPSC Generation	Differentiation Potential	Characteristics	Functions	Limitations	References
HiPSC	Human somatic cells	Induced pluripotent stem cells	Multilineage	Expression of pluripotency markers	Cell replacement therapy, drug discovery	Epigenetic memory, variability in differentiation potential	[54]
MiPSC	Mouse somatic cells	Induced pluripotent stem cells	Multilineage	Expression of pluripotency markers	Disease modeling, developmental biology	Tumorigenic potential, incomplete reprogramming	[55]
CiPSC	Chimpanzee fibroblasts	Induced pluripotent stem cells	Multilineage	Expression of pluripotency markers	Comparative genomics, conservation biology	Limited availability, ethical considerations	[56]
siPSC	Porcine somatic cells	Induced pluripotent stem cells	Multilineage	Expression of pluripotency markers	Xenotransplantation, agriculture	Limited availability, ethical considerations	[57]
EpiSC	Mouse or human embryonic cells	Epiblast stem cells	Limited differentiation potential	Expression of pluripotency markers	Developmental biology, tissue engineering	Unstable in culture, low efficiency of reprogramming	[58]
FiPSC	Feline somatic cells	Induced pluripotent stem cells	Multilineage	Expression of pluripotency markers	Disease modeling, regenerative medicine	Limited availability, ethical considerations	[59]
NiPSC	Canine somatic cells	Induced pluripotent stem cells	Multilineage	Expression of pluripotency markers	Regenerative medicine, veterinary research	Limited availability, ethical considerations	[60]
ViPSC	Viral vectors	Induced pluripotent stem cells	Multilineage	Expression of pluripotency markers	Gene therapy, disease modeling	Safety concerns, potential for viral integration	[61]
CiPSC	Chemical compounds	Chemical-induced pluripotent stem cells	Multilineage	Expression of pluripotency markers	Disease modeling, regenerative medicine	Limited efficiency, potential toxicity	[21]
SiPSC	Synthetic transcription factors	Synthetic-induced pluripotent stem cells	Multilineage	Expression of pluripotency markers	Disease modeling, drug discovery	Limited efficiency, potential off-target effects	[62]
AiPSC	Human amniotic fluid cells	Induced pluripotent stem cells	Multilineage	Expression of pluripotency markers	Cell replacement therapy, disease modeling	Limited availability, potential for genetic abnormalities	[63]
PiPSC	Porcine pluripotent stem cells	Genetic engineering	Multilineage	Expression of pluripotency markers	Tissue engineering, regenerative medicine	Limited availability, ethical considerations	[64]
GiPSC	Germline cells	Induced pluripotent stem cells	Limited differentiation potential	Expression of germline markers	Reproductive biology, disease modeling	Ethical concerns, low efficiency	[65]
LiPSC	Lipoblasts	Induced pluripotent stem cells	Adipogenic differentiation	Expression of adipogenic markers	Adipose tissue engineering, disease modeling	Limited differentiation potential	[63]
XiPSC	Xenopus tropicalis tadpole cells	Induced pluripotent stem cells	Multilineage	Expression of pluripotency markers	Developmental biology, regeneration research	Limited availability, potential ethical considerations	[64]
NiPSC	Neonatal foreskin fibroblasts	Induced pluripotent stem cells	Multilineage	Expression of pluripotency markers	Disease modeling, regenerative medicine	Limited availability	[66]
TiPSC	Testicular cells	Induced pluripotent stem cells	Limited differentiation potential	Expression of germline markers	Reproductive biology, disease modeling	Ethical concerns, low efficiency	[47]
ZiPSC	Zebrafish embryos	Induced pluripotent stem cells	Multilineage	Expression of pluripotency markers	Developmental biology, regeneration research	Limited availability, potential ethical considerations	[67]
AiPSC	Adult neural stem cells	Induced pluripotent stem cells	Neural differentiation	Expression of neural markers	Neural regeneration, disease modeling	Limited differentiation potential	[68]
DiPSC	Dental pulp stem cells	Induced pluripotent stem cells	Mesenchymal differentiation	Expression of mesenchymal markers	Dental tissue engineering, disease modeling	Limited differentiation potential	[42]
CiPSC	Corneal stromal cells	Induced pluripotent stem cells	Corneal differentiation	Expression of corneal markers	Corneal tissue engineering, disease modeling	Limited differentiation potential	[43]
AiPSC	Aortic endothelial cells	Induced pluripotent stem cells	Endothelial differentiation	Expression of endothelial markers	Vascular tissue engineering, disease modeling	Limited differentiation potential	[44]
OiPSC	Ovarian surface epithelial cells	Induced pluripotent stem cells	Limited differentiation potential	Expression of ovarian markers	Reproductive biology, disease modeling	Limited differentiation potential	[45]
SiPSC	Skeletal muscle cells	Induced pluripotent stem cells	Myogenic differentiation	Expression of myogenic markers	Skeletal muscle tissue engineering, disease modeling	Limited differentiation potential	[46]
TiPSC	Tonsil-derived cells	Induced pluripotent stem cells	Multilineage	Expression of pluripotency markers	Disease modeling, regenerative medicine	Limited availability	[47]
FiPSC	Fat-derived cells	Induced pluripotent stem cells	Adipogenic differentiation	Expression of adipogenic markers	Adipose tissue engineering, disease modeling	Limited differentiation potential	[48]
LiPSC	Liver cells	Induced pluripotent stem cells	Hepatic differentiation	Expression of hepatic markers	Liver tissue engineering, disease modeling	Limited differentiation potential	[49]
RiPSC	Retinal pigment epithelial cells	Induced pluripotent stem cells	Retinal differentiation	Expression of retinal markers	Retinal tissue engineering, disease modeling	Limited differentiation potential	[69]
KiPSC	Kidney cells	Induced pluripotent stem cells	Renal differentiation	Expression of renal markers	Renal tissue engineering, disease modeling	Limited differentiation potential	[70]
SiPSC	Salivary gland-derived cells	Induced pluripotent stem cells	Limited differentiation potential	Expression of salivary gland markers	Salivary gland tissue engineering, disease modeling	Limited differentiation potential	[71]
LiPSC	Liver cells	Induced pluripotent stem cells	Hepatic differentiation	Expression of hepatic markers	Liver tissue engineering, disease modeling	Limited differentiation potential	[70]
RiPSC	Retinal pigment epithelial cells	Induced pluripotent stem cells	Retinal differentiation	Expression of retinal markers	Retinal tissue engineering, disease modeling	Limited differentiation potential	[70]
KiPSC	Kidney cells	Induced pluripotent stem cells	Renal differentiation	Expression of renal markers	Renal tissue engineering, disease modeling	Limited differentiation potential	[72]
SiPSC	Salivary gland-derived cells	Induced pluripotent stem cells	Limited differentiation potential	Expression of salivary gland markers	Salivary gland tissue engineering, disease modeling	Limited differentiation potential	[70]

### How iPSCs are derived

**Fig. 3:**
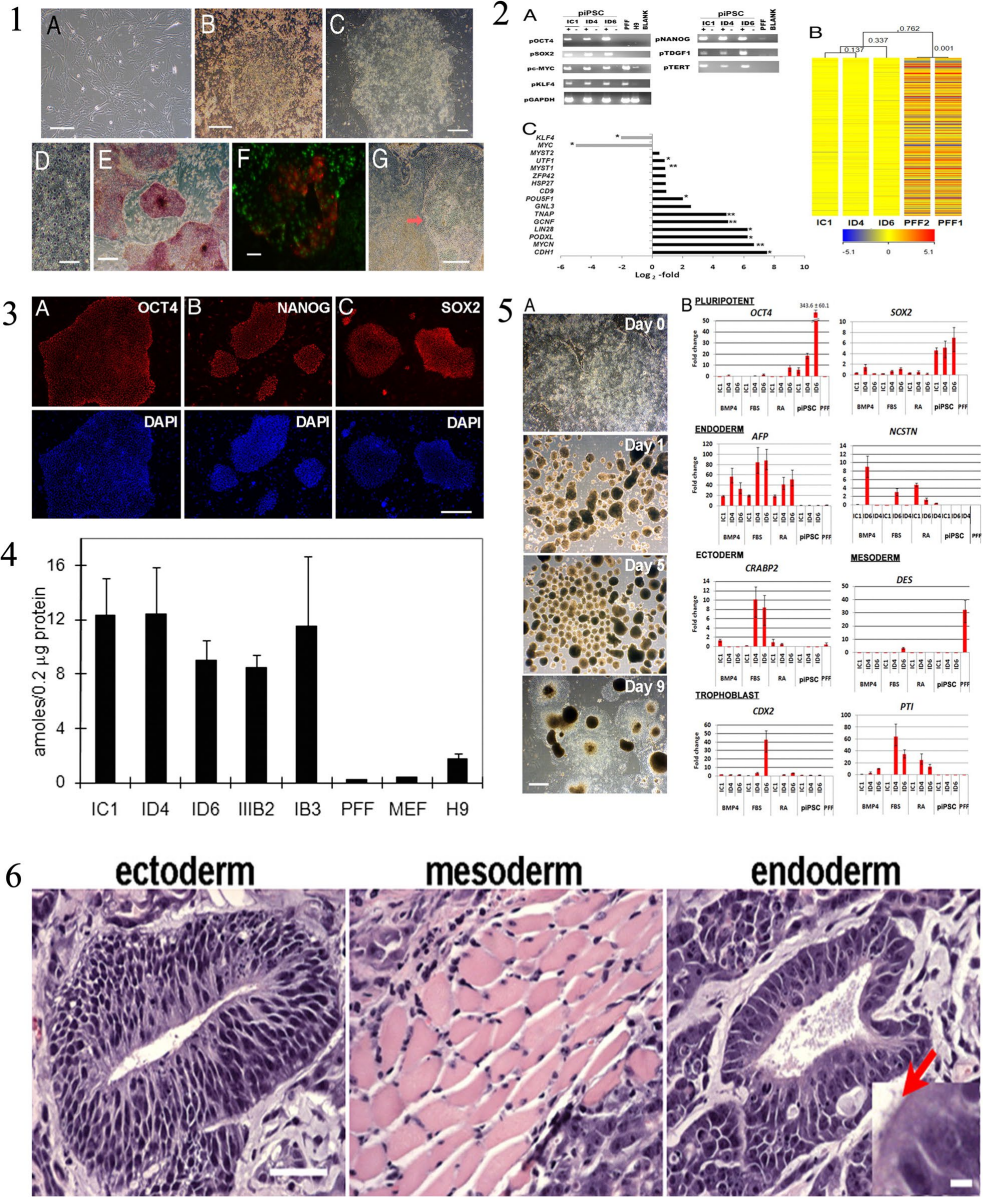
**1** The process of generating piPSC colonies from PFF (pluripotent stem cells derived from preimplantation embryos). (A) The first image is a phase contrast image of PFF. (B) The second image shows granulated piPSC colonies similar to mouse and human iPSC that begin to appear approximately three weeks after viral infection. (C) The third image represents a representative piPSC colony after multiple passages, resembling hESC (human embryonic stem cells), shown at a lower magnification. (D) The fourth image is a higher magnification of the same piPSC colony shown in (C). (E) The piPSC colonies express alkaline phosphatase (AP), as depicted in the image. (F) The image shows nuclear localization of OCT4 (green) and surface SSEA1 (red) in the piPSC colonies. (G) Some piPSC colonies have a tendency to undergo spontaneous differentiation, as indicated by the area (arrow) on the right side of the colony. The differentiated cells exhibit cobblestone morphology with a relatively low nucleus to cytoplasm ratio. **2** The results of gene expression analysis conducted on piPSC (porcine-induced pluripotent stem cells) compared to PFF (porcine fetal fibroblast) and H9 hESC (human embryonic stem cells). The analysis involved different techniques, as described in the following paragraphs. In panel A, the researchers used RT-PCR (Reverse Transcription Polymerase Chain Reaction) to examine the expression of specific pluripotency genes in piPSC, PFF, and H9 hESC. The primers used were designed to target porcine genes rather than their human counterparts. However, it was observed that the primers for pc-MYC and pKLF4 also showed some level of cross-reactivity. Panel B displays the results of hierarchical clustering analysis performed on microarray data from three piPSC lines (IC1, ID4, and ID6) and two PFF cells (1 and 2). The clustering was based on Pearson-centered single-linkage rule, and it aimed to identify patterns of gene expression similarity or dissimilarity among the samples. The analysis included all genes (totaling 8,015) that exhibited a fold-change of at least 1.3 in their normalized expression between piPSC and PFF, with a significance level (P value) of 0.05 or lower. The values indicated next to the branches represent Pearson distances, which indicate the degree of dissimilarity between the gene expression profiles. In panel C, the fold differences (Log2) in gene expression between piPSC and PFF are presented. The black bars on the right-hand side of the axis represent genes that were up-regulated (showed increased expression) in piPSC compared to PFF, while the gray bars on the left side represent down-regulated (showed decreased expression) genes. The significance of the differences was assessed using P values, with '*' indicating a significance level of 0.05 or lower and '**' indicating a significance level of 0.01 or lower. **3** The results of immunofluorescence staining carried out on piPSC colonies cultured on MEF, focusing on pluripotent markers. The upper panels (A, B, and C) depict the immunofluorescence staining of OCT4, NANOG, and SOX2 respectively. The lower panels (A–C) confirm the specific localization of these markers to the nuclei, as indicated by the blue staining with DAPI. **4** The measurement of telomerase activity in different types of cells. The telomerase activities of several piPSC lines (IC1 passage 10, ID4 passage 10, ID6 passage 10, IIIB2 passage 3, and IB3 passage 8) are compared to their parental cells, including EGFP-PFF passage 10, MEF passage 4, and H9 hESC passage 41. The assay was conducted using triplicate samples, each containing 0.2 μg of total cell protein, and the TRAPESE-RT Telomerase Detection Kit (Chemicon) was utilized. The telomerase activity is represented by the value in amole, which indicates the number of extended primers containing telomeric repeats. **5** The process of differentiating piPSC (pluripotent induced pluripotent stem cells) into embryoid bodies (EB). In Part A, Day 0 shows piPSC cells plated on MEF (mouse embryonic fibroblasts). Day 1 shows an image of the resulting EB obtained on the next day, while Day 5 displays an image after 5 days of differentiation. Finally, Day 9 exhibits cells treated with 5% FBS (fetal bovine serum) for a duration of 9 days. Part B presents the results of real-time RT-PCR analysis, which measures the relative concentrations of transcript molecules of pluripotent and lineage-specific genes in various cell lines. These cell lines include piPSC lines (IC1, ID4, and ID6), PFF (pluripotent fetal fibroblasts), and piPSC that were differentiated into EB using BMP4, FBS, or retinoic acid (RA) as differentiation agents. The y-axis represents the fold change relative to the expression of GAPDH (glyceraldehyde 3-phosphate dehydrogenase), which is a reference gene commonly used in gene expression studies. **6** A microscopic image of a tumor taken from the peritoneum of a hairless mouse. The tumor, which was surgically removed, was formed by injecting cells from the piPSC line ID6 under the skin of the mouse. The tumor exhibited a high level of differentiation and consisted of various types of tissues. These tissues included neural epithelium (ectoderm) on the left side, striated muscle (mesoderm) in the middle, and epithelium with a brush border (endoderm) on the right side. The magnification used for all three tissues is the same. An inset on the right side provides a closer view of the brush border, indicated by a red arrow, and the scale bar in the image corresponds to 5 μm. Reprinted from [83] with permission from the PNAS

The iPSCs are derived through a groundbreaking technique that reprograms adult cells, enabling them to regain the pluripotent state similar to ESCs [73]. This discovery, made by Shinya Yamanaka in 2006, opened up new avenues in regenerative medicine, disease modeling, and drug discovery. Understanding how iPSCs are derived is essential to appreciate their potential and the implications for various fields of research and therapy [23]. The process of iPSC derivation involves the introduction of specific reprogramming factors into somatic cells, which are differentiated adult cells. These reprogramming factors can reset the cellular state, erasing the specialized characteristics of the somatic cells and reverting them back to a pluripotent state [74]. The most commonly used reprogramming factors are Oct4 (octamer-binding transcription factor 4), Sox2 (sex-determining region Y-box 2), Klf4 (Kruppel-like factor 4), and c-Myc (avian myelocytomatosis viral oncogene homolog) [20]. These factors work together to induce the expression of genes associated with pluripotency, while simultaneously repressing genes involved in cellular differentiation. The first step in iPSC derivation involves obtaining somatic cells from an individual [75]. These cells can be sourced from various tissues, including skin fibroblasts, blood cells, or even urine-derived cells. The choice of cell type depends on the research goals and the ease of accessibility [19]. Once the somatic cells are isolated, they are cultured in a laboratory setting and prepared for reprogramming. The reprogramming process typically involves the use of viral vectors or non-integrating methods to introduce the reprogramming factors into the somatic cells. Viral vectors, such as retroviruses or lentiviruses, have been widely used in the past. These vectors deliver the reprogramming factors into the somatic cells’ DNA, integrating the reprogramming genes into the host [76]. However, this method poses the risk of insertional mutagenesis and unwanted genetic changes. To address these concerns, researchers have developed non-integrating methods, such as the use of episomal plasmids, mRNA, proteins, or small molecules, which do not integrate into the host genome [77]. After the introduction of the reprogramming factors, the somatic cells undergo a transformation process. Over time, the cells gradually lose their original characteristics and acquire pluripotent features. The reprogramming factors initiate changes in gene expression patterns, leading to the reactivation of pluripotency-associated genes and the suppression of somatic cell-specific genes. This transformation can be visually observed, as the cells transition from a flat, adherent morphology to a distinct colony-like structure resembling ESCs [40]. The reprogramming process typically takes a few weeks, during which the cells are subjected to specific culture conditions to support their transition. These conditions often include the use of culture media supplemented with growth factors and small molecules that enhance the reprogramming efficiency [35]. The media composition and culture conditions vary depending on the specific protocols and the desired outcome of iPSC derivation. Once the iPSC colonies have formed, they are isolated and expanded for further characterization and experimentation. These iPSCs exhibit key characteristics of ESCs, such as self-renewal capacity and the ability to differentiate into cells of all three germ layers: ectoderm, endoderm, and mesoderm. iPSCs can be maintained in culture for prolonged periods, allowing for the generation of large quantities of cells for downstream applications [78]. The quality of derived iPSCs is crucial, as it affects their usability in various research and therapeutic applications. iPSCs must undergo rigorous characterization to ensure their pluripotent state and genomic integrity. Techniques such as immunostaining, gene expression analysis, and karyotyping are employed to confirm the expression of pluripotency markers and to assess the absence of genetic abnormalities or chromosomal aberrations. Immunostaining involves the use of specific antibodies to detect the presence of pluripotency markers, such as Oct4, Nanog, SSEA-4, and Tra-1–60, in iPSC [79]. Positive staining for these markers indicates that the cells have successfully acquired pluripotency and resemble ESCs. Additionally, gene expression analysis, such as reverse transcription-polymerase chain reaction (RT-PCR) or RNA sequencing, is performed to confirm the activation of pluripotency-associated genes and the silencing of somatic cell-specific genes [80]. Karyotyping, on the other hand, is a technique used to examine the chromosomal composition of iPSCs. It helps identify any chromosomal abnormalities or genetic mutations that may have occurred during the reprogramming process. This step is essential to ensure the genomic stability of iPSCs and to avoid potential issues related to aberrant chromosomal rearrangements [81]. After thorough characterization, the derived iPSCs can be used for a wide range of applications. They serve as a valuable tool for disease modeling, allowing researchers to study the underlying mechanisms of various diseases by generating patient-specific iPSCs. These iPSCs can be differentiated into specific cell types affected by the disease, providing a platform for understanding disease progression and developing personalized therapeutic strategies. Moreover, iPSCs hold great promise for drug discovery and screening [82]. In the field of regenerative medicine, iPSCs offer the potential for personalized cell-based therapies. By reprogramming a patient’s own somatic cells, iPSCs can be generated with the same genetic makeup as the individual, reducing the risk of immune rejection. These iPSCs can be differentiated into the desired cell types and used for transplantation, aiming to replace damaged or diseased tissues and organs [1, 7]. In a recent study conducted by Ezashi et al. (2009), the researchers successfully derived iPSCs from pig somatic cells, addressing the challenges associated with generating embryonic stem cells (ESCs) from ungulates [83]. Ezashi et al. employed lentiviral transduction of four human genes (hOCT4, hSOX2, hKLF4, and hc-MYC) commonly used for iPSC generation in mice and humans [83]. The iPSCs were derived from porcine fetal fibroblasts and cultured on irradiated mouse embryonic fibroblasts (MEFs) in a medium supplemented with knockout serum replacement and FGF2. Figure 3 of the study illustrates the process of generating piPSC colonies from plated fibroblasts (PFF), showcasing the successful reprogramming of porcine somatic cells. Furthermore, gene expression analysis conducted on the piPSCs is depicted in Fig. 3, confirming their pluripotent nature. Immunofluorescence staining results of piPSC colonies cultured on MEF can be seen in Fig. 3, providing visual evidence of their characteristics. Figure 3 compares the telomerase activity in different cell lines, demonstrating the high telomerase activity exhibited by the derived piPSCs, indicative of their cellular immortality. Moreover, the role of iPSCs in the study is particularly highlighted in Fig. 3, which shows the differentiation of pluripotent iPSCs into embryoid bodies (EB), representing the potential of these cells to give rise to various tissue types. Additionally, Fig. 3 presents a histological cross-section of a solid tumor surgically removed from the peritoneum of a hairless mouse, showcasing the in vivo potential and versatility of the piPSCs. Overall, this recent study successfully generated piPSCs from porcine somatic cells, shedding light on their potential applications in regenerative medicine, tissue engineering, and preclinical studies. The derivation of iPSCs involves the reprogramming of adult somatic cells to regain their pluripotent state. Through the introduction of specific reprogramming factors, somatic cells undergo a transformation process, losing their specialized characteristics and acquiring pluripotent features. The reprogramming process can be achieved using viral vectors or non-integrating methods [76]. The derived iPSCs must undergo rigorous characterization to confirm their pluripotency and genomic integrity. iPSCs hold immense potential in various fields, including disease modeling, drug discovery, and regenerative medicine, offering opportunities for personalized therapies and advancing our understanding of human development and disease [1, 7]. Continued research in iPSC technology will undoubtedly unveil further advancements and applications, paving the way for innovative approaches in biomedical research and clinical practice [23].

### Characteristics of iPSCs and their potential in regenerative medicine

#### Characteristics of iPSCs

**Fig. 4:**
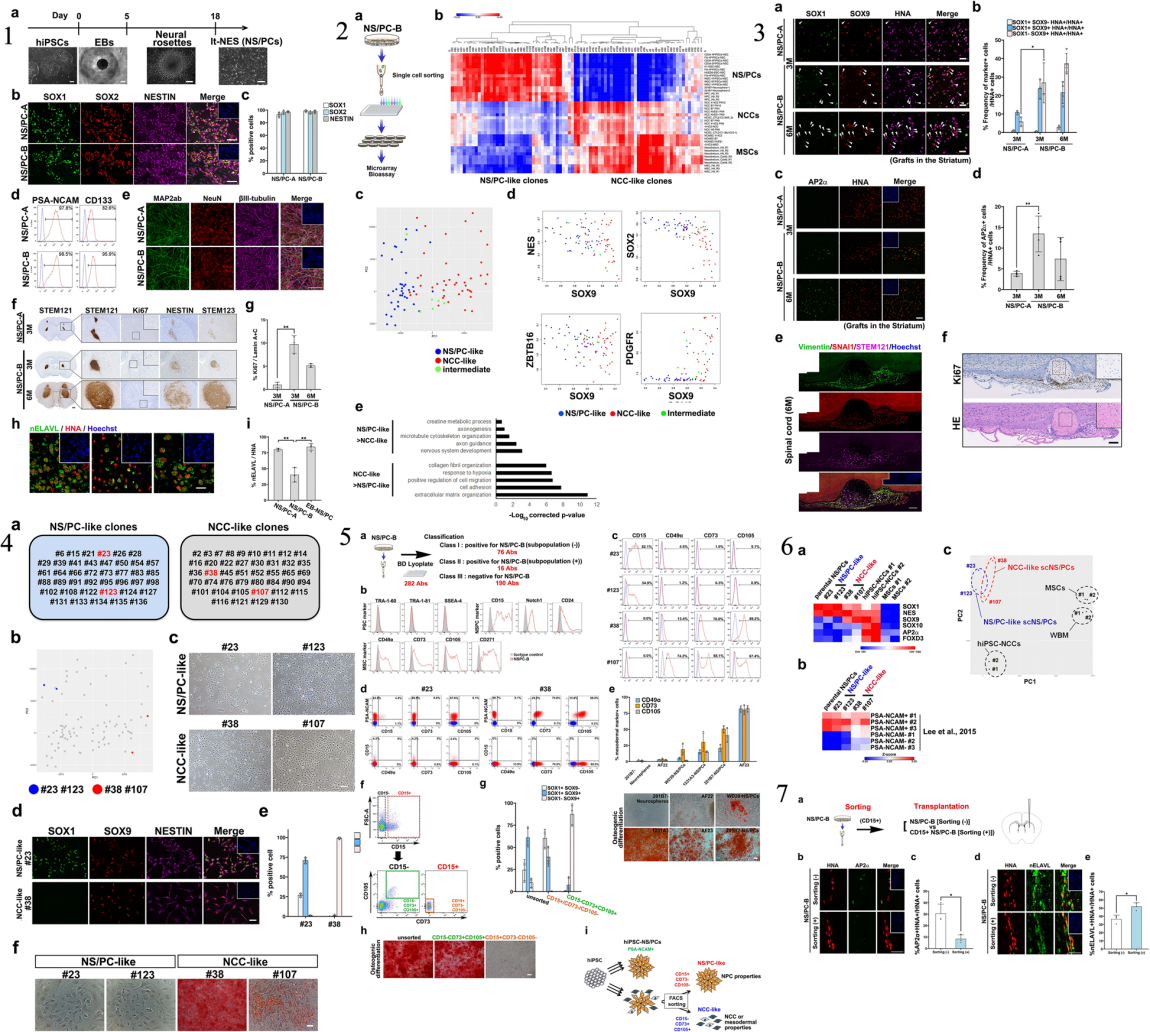
**1** The characterization of neural stem/progenitor cells (NS/PCs) derived from human induced pluripotent stem cells (hiPSCs). In panel (a), the process of generating NS/PCs from feeder-free cultured hiPSCs is depicted, accompanied by representative images of cells at each stage of differentiation. The scale bar in the image is 200 μm. Panels (b) and (c) show representative immunocytochemical images (b) and quantification (c) of hiPSC-NS/PCs using specific antibodies against SOX1, SOX2, and NESTIN. The inset in panel (b) displays Hoechst nuclear staining of the same sample, and the scale bar is 50 μm. Panel (d) presents representative images of cell surface markers PSA-NCAM and CD133 on hiPSC-NS/PCs. The differentiation capacity of hiPSC-NS/PCs is demonstrated in panel (e) with representative images of neuronal differentiation for each cell line. Neuronal markers, including MAP2ab (green), NeuN (red), and βIII-tubulin (purple), are expressed after 14 days of differentiation. The scale bar in the image is 100 μm. Panel (f) shows histological evaluation of hiPSC-NS/PCs after transplantation into immunodeficient mice. Representative tissue sections of the striatum are displayed, and graft survival is assessed using the marker STEM121, which indicates human cytoplasm. The differentiation capacity of hiPSC-NS/PCs in the graft is evaluated using antibodies against Ki67, NESTIN, and human-specific GFAP (STEM123). Insets in the panel provide a closer look at the Ki67 signal in specific regions. The scale bars in the images are 500 μm. Panel (g) quantifies the number of Ki67 + cells among human-specific Lamin A + C + cells at the indicated time point. Panels (h) and (i) demonstrate neuronal differentiation of hiPSC-NS/PCs after transplantation, as indicated by the expression of the neuronal marker nELAVL in HNA + grafts. The insets in panel (h) show Hoechst nuclear staining of the same sample, and the scale bar is 20 μm. Quantification of neuronal differentiation is shown in panel (i). Statistical values are provided as means ± standard deviation, and asterisks indicate statistical significance (NS/PC-A, *n* = 3; NS/PC-B, *n* = 3; EB-NS/PC, *n* = 4, **p < 0.01). **2** The examination of the variability within hiPSC-NS/PCs (human induced pluripotent stem cell-derived neural stem/progenitor cells) using a single-cell-based method. a) The diagram illustrates the process of fluorescence-activated cell sorting (FACS) of NS/PC-B, followed by cell expansion for subsequent biological analyses. b) The figure displays a correlation analysis between gene expression profiles of single-cell-derived NS/PCs (scNS/PCs) obtained through microarray analysis and gene expression in NS/PCs, neural crest cells (NCCs), and MSCs from publicly available datasets. The clustering of these profiles is also presented, with the color indicating the significance of correlation (z-value). c) Principal component analysis of scNS/PCs is shown. NS/PC-like scNS/PCs are represented by red dots, NCC-like scNS/PCs by blue dots, and unclassified scNS/PCs (intermediate scNS/PCs) by light green dots. d) A comparison of gene expression related to neural (NES, SOX2, and ZBTB16) and mesodermal (SOX9 and PDGFR) lineages is demonstrated in NS/PC-like (blue), intermediate (light green), and NCC-like (red) scNS/PCs. e) The figure presents a gene ontology (GO) analysis of differentially expressed genes in NS/PC-like scNS/PCs compared to NCC-like scNS/PCs. **3** The presence of cells displaying mesodermal characteristics in grafts derived from human-induced pluripotent stem cell-derived neural stem/progenitor cells (hiPSC-NS/PCs). In panel (a), the grafts in the striatum were examined histologically using antibodies against SOX1 and SOX9, and the arrows highlight cells that are positive for SOX1 and SOX9 among the HNA-positive cells. The scale bar represents a length of 25 μm. Panel (b) provides a quantification of the SOX1-SOX9 positive cells in the grafts, with the mean values and standard deviations indicated [NS/PC-A (3M) *n* = 3; NS/PC-B (3M) *n* = 3; NS/PC-B (6M) *n* = 4, *p < 0.05]. In panel (c), representative images show the expression of AP2α in the NS/PC-derived grafts in the striatum, with the inset demonstrating Hoechst nuclear staining of the same field. Panel (d) quantifies the frequency of AP2α-positive cells in the grafts, with mean values and standard deviations provided [NS/PC-A (3M) *n* = 4; NS/PC-B (3M, 6M) *n* = 4, **p < 0.01]. Panel (e) displays representative images of Vimentin and SNAI1 expression in STEM121-positive grafts six months after transplantation into an injured spinal cord. The scale bar represents a length of 100 μm. Panel (f) presents a bone-like structure derived from the grafts in the injured spinal cord region. Immunohistochemical staining of Ki67 (upper panel) and H&E staining (lower panel) of serial sections corresponding to the area shown in (e) is shown. The inset provides a higher magnification of the boxed field. The scale bar represents a length of 100 μm. **4** The osteogenic differentiation capacity of neural stem/progenitor cells (NS/PCs) that have characteristics resembling neural crest cells (NCCs). Panel (a) provides detailed information about the cluster numbers within NS/PCs and NCC-like NS/PCs, with the selected cells for further analysis highlighted in red. Panel (b) shows a principal component analysis (PCA) plot of the transcriptome in the NS/PCs, with additional information about the selected NS/PCs highlighted in blue (NS/PC-like) and red (NCC-like). Panel (c) presents representative images of the selected NS/PCs, with a scale bar of 100 μm for size reference. Panel (d) displays the results of immunocytochemical analysis of the NS/PC-like and NCC-like NS/PCs using antibodies against SOX1 (green), SOX9 (red), and NESTIN (purple). The inset in this panel shows Hoechst nuclear staining of the same field, with a scale bar of 50 μm. Panel (e) provides quantification data based on the immunocytochemical analysis shown in panel (d). Finally, panel (f) shows the results of Alizarin red S staining after osteogenic differentiation of the NS/PC-like and NCC-like NS/PCs. The scale bar in this panel is 100 μm. **5** The process of identifying specific cell surface markers to determine populations that possess the ability to generate bone tissue. In part (a), a screening was conducted on a subset of hiPSC-NS/PCs (human-induced pluripotent stem cell-derived neural stem/progenitor cells) using the BD Lyoplate screening panel. The results from flow cytometry categorized the antibodies into three groups. Part (b) shows the flow cytometric analysis of cell surface markers for pluripotent stem cells (PSCs), NS/PCs, and MSCs on NS/PC-B cells. Part (c) validates the cell surface marker screening using NS/PC-like and NCC-like scNS/PCs (single-cell-derived NS/PCs and neural crest cell-like NS/PCs). Flow cytometric analysis displays the frequencies of cells expressing the antigens. Part (d) provides a representative image of coexpression analysis between NS/PC markers and NCC markers on NS/PC-like and NCC-like scNS/PCs. In part (e), the expression of NCC markers on various types of iPSC-NS/PCs is evaluated, along with representative images of Alizarin red S staining after inducing osteogenic differentiation. Part (f) involves sorting NS/PC-B cells based on CD15, CD73, and CD105 expression. The sorted cells are then subjected to further evaluation. Part (g) quantifies the sorted fractions based on SOX1 and SOX9 expression. Part (h) examines the sorted cells for their ability to differentiate into bone cells using Alizarin red S staining. Finally, part (i) presents a proposed model for the cellular heterogeneity of hiPSC-NS/PCs. **6** The transcriptome characteristics of iPSC-NS/PCs in comparison to authentic NCCs and MSCs. In panel (a), a heatmap demonstrates the expression levels of genes associated with NS/PCs (SOX1 and NES) and genes associated with NCCs (SOX9, SOX10, AP2α, and FOXD3) in parental NS/PCs, scNS/PCs, hiPSC-NCCs, and MSCs. Panel (b) displays a heatmap that shows the correlation in gene expression between parental NS/PCs and scNS/PCs with gene expression data from previously published datasets of PSA-NCAM + and PSA-NCAM- NS/PCs. The color scale represents the z-value, indicating the significance of the correlation. In panel (c), a principal component analysis is presented, comparing scNS/PCs with referenced cells such as hiPSC-NCCs, WBM, and MSCs. **7** The process of ensuring the quality of neural stem/progenitor cells (NS/PCs) through purification using CD15. In panel (a), a diagram shows the transplantation of NS/PCs derived from NS/PC-B, either sorted with an anti-CD15 antibody [sorting ( +)] or without it [sorting (-)], into the striatum of immunodeficient mice. After 10 weeks, immunohistochemical analysis was conducted to evaluate the differentiation capacity of the transplanted NS/PCs. Representative images (b) and corresponding quantification (c) demonstrate the expression of AP2α in HNA + grafts as an indicator of differentiation capacity. The insets in panel (b) display Hoechst nuclear staining of the same area. Quantitative data is presented in the right panel. The scale bar represents 50 μm. Mean values ± standard deviation (*n* = 3, *p < 0.05) are provided. Similarly, representative images (d) and quantification (e) show the expression of nELAVL in HNA + grafts to assess the differentiation capacity of the transplanted NS/PCs. The insets in panel (d) display Hoechst nuclear staining of the same area. Reprinted from [84] with permission from the Springer Nature

The iPSCs possess several key characteristics that make them highly valuable in regenerative medicine and disease research [1, 7]. The iPSCs possess several key characteristics that are highly relevant to the characterization of neural stem/progenitor cells (NS/PCs) derived from human iPSCs. Firstly, iPSCs share the defining feature of pluripotency, meaning they have the potential to differentiate into cells of all three germ layers, including neural cells [70]. This property allows for the generation of NS/PCs from iPSCs, serving as a valuable model system for studying neural development and disease. Secondly, iPSCs can be derived from adult somatic cells, such as skin cells, through reprogramming techniques, avoiding the ethical concerns associated with embryonic stem cells [1, 7]. This enables the generation of patient-specific iPSC lines, allowing for personalized medicine approaches and the study of neurological disorders using patient-derived cells. Additionally, iPSCs exhibit self-renewal capacity, allowing them to proliferate indefinitely in culture, thereby providing a sustainable source of NS/PCs for experimentation and potential therapeutic applications. Lastly, iPSCs can be genetically modified to introduce specific mutations or gene editing techniques, facilitating the investigation of genetic factors underlying neural development and diseases [70]. Isoda et al., the research focuses on understanding the tumorigenic potential of neural stem/progenitor cells derived from human induced pluripotent stem cells (hiPSC-NS/PCs), which are considered a promising source for cell-based therapies. The study establishes single cell-derived NS/PC clones (scNS/PCs) from hiPSC-NS/PCs that produced undesired grafts after transplantation. Through bioassays, the researchers identified unique subsets of scNS/PCs with a transcriptome signature resembling mesenchymal lineages. These scNS/PCs expressed both neural and mesenchymal markers and possessed osteogenic differentiation capacity. Significantly, removing CD73 + CD105 + cells from the parental hiPSC-NS/PC population was found to enhance the quality of hiPSC-NS/PCs and mitigate their tumorigenic potential. This research highlights the presence of unexpected cell populations within NS/PCs, shedding light on the tumorigenicity concerns associated with hiPSC-NS/PCs in the context of regenerative medicine [84]. Figure 4 provided a characterization of NS/PCs derived from hiPSCs, highlighting the importance of hiPSCs as a source for generating these neural cells. The study utilized a single cell-based approach, as depicted in Fig. 4, to analyze the variations within hiPSC-NS/PCs. This comprehensive analysis allowed the researchers to identify distinct subsets of scNS/PCs with a transcriptome signature indicative of mesenchymal lineages. Figure 4 demonstrated the presence of cells displaying mesodermal properties in grafts derived from hiPSC-NS/PCs, underscoring the importance of understanding the heterogeneity within the NS/PC population. Furthermore, Fig. 4 showcased the osteogenic differentiation capacity of NCC-like scNS/PCs, suggesting their potential to differentiate into bone-forming cells. To identify specific cell surface markers associated with osteogenic capacity, the researchers employed a strategy illustrated in Fig. 4. This allowed for the identification and purification of desired cell populations within hiPSC-NS/PCs, which is crucial for maintaining their quality and safety. Figure 4 presented a comparative analysis of the transcriptome signature of iPSC-NS/PCs, bona fide NCCs, and MSCs. This analysis shed light on the unique characteristics of iPSC-NS/PCs and their resemblance to both neural and mesenchymal lineages. Lastly, Fig. 4 demonstrated the process of purifying NS/PCs using CD15 as a marker to ensure the quality of the cell population. Overall, this recent study highlighted the mesenchymal properties observed in specific subsets of hiPSC-NS/PCs and their potential contribution to tumorigenicity. The findings emphasize the importance of understanding and characterizing the different cell populations within hiPSC-NS/PCs to enhance their safety and efficacy for future regenerative medicine applications. The study showcased the valuable role of iPSCs in generating NS/PCs and provided insights into their transcriptome signature, osteogenic differentiation capacity, and purification methods using specific cell surface markers.

##### Pluripotency

**Fig. 5:**
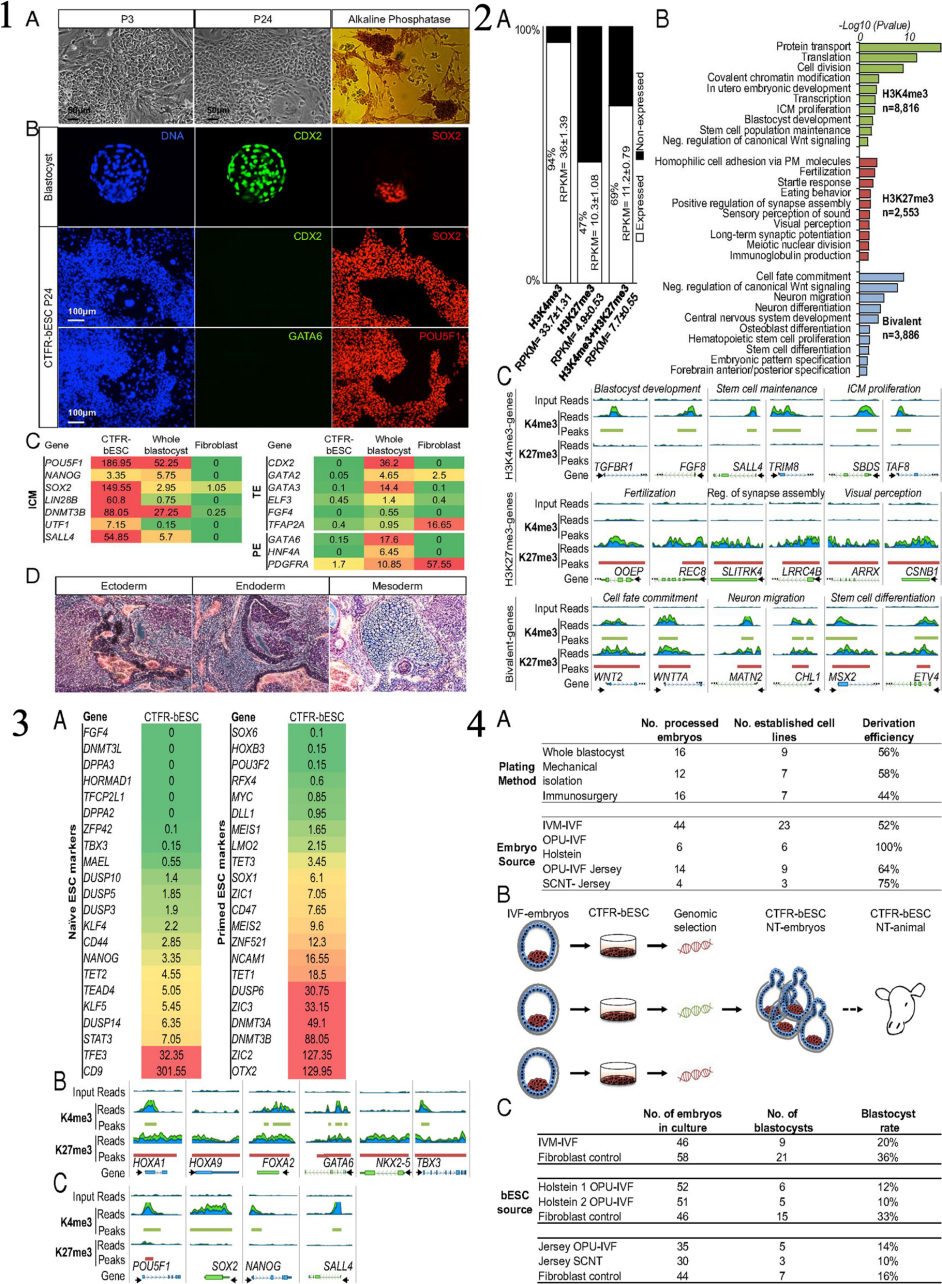
**1** The process and characterization of CTFR-bESCs. In panel A, bright-field images and AP staining are shown, illustrating the typical colony morphologies of CTFR-bESCs. It is important to note that the feeder layer in the images is negative for AP staining. The passages shown are P3 (passage 3) and P24 (passage 24). The scale bars in the images represent a length of 50 μm. Panel B displays immunofluorescence (IF) staining for various markers, including SOX2, POU5F1, GATA6, and CDX2. The top row shows bovine blastocysts at a magnification of 20 × objective, while the middle and bottom rows show CTFR-bESCs. Panel C presents the results of expression analysis for markers specific to different lineages: ICM (inner cell mass), TE (trophectoderm), and PE (primitive endoderm). The analysis was performed using RNA-seq, and the samples include two independent CTFR-bESC lines (P10), two independent pools of whole blastocysts (10 each), and two lines of bovine fibroblasts. The color scale indicates expression levels, ranging from red (high expression) to green (low/no expression). In panel D, representative images exhibit H&E staining of histological sections obtained from teratomas generated by CTFR-bESCs. These teratomas contain tissues from all three germ lineages: ectoderm, endoderm, and mesoderm. The magnification used for these images is 10 × . **2** The pattern of histone methylation in CTFR-induced pluripotent stem cells (CTFR-bESCs). In part (A), the transcriptional status of genes containing H3K4me3, H3K27me3, or bivalent domains is depicted. Genes with an RPKM (Reads Per Kilobase Million) value of 0.4 or higher are considered expressed, while genes with an RPKM value below 0.4 are considered nonexpressed. The bar plot inside the figure shows the average RPKM values ± SEM (Standard Error of the Mean) for expressed genes, while the x-axis displays the average RPKM values ± SEM for all genes (both expressed and nonexpressed). In part (B), the functional characteristics of genes containing H3K4me3, H3K27me3, or bivalent domains are presented. The figure displays the top 10 Gene Ontology (GO) terms associated with these genes. The bar plot represents the negative logarithm (base 10) of the P-value for selected GO terms related to biological processes, as determined by DAVID (Database for Annotation, Visualization, and Integrated Discovery). In part (C), a snapshot of the genome browser is provided, showing specific genes associated with H3K4me3, H3K27me3, or bivalent domains. The genes are listed for each category, such as TGFBR1, FGF8, SALL4, TRIM8, SBDS, and TAF8 for H3K4me3; OOEP, REC8, SLITRK4, LRRC4B, ARRX, and CSNB1 for H3K27me3; and WNT2, WNT7A, MATN2, CHL1, MSX2, and ETV4 for bivalent domains. These genes are associated with three distinct GO terms. The start of each gene is indicated by a black arrow in the genome browser snapshot. **3** The molecular characteristics of CTFR-bESCs, indicating their state of primed pluripotency. In panel A, the expression levels of specific markers for naive and primed pluripotency were analyzed using RNA-seq, and the results are represented using red (expressed genes with RPKM ≥ 0.4) and green (nonexpressed genes with RPKM < 0.4) color-coding. The data shown are the means of two independent biological replicates. Panel B provides snapshots from a genome browser displaying the histone methylation profiles of markers associated with primed and naive pluripotency in CTFR-bESCs. Panel C displays genome browser snapshots of H3K4me3 and H3K27me3 marks on key pluripotency genes (POU5F1, SOX2, NANOG, SALL4) in CTFR-bESCs. **4** The potential applications of CTFR-bESCs (Chimeric Trained Functional RNA-blastocyst-derived Embryonic Stem Cells) in genomic selection. In part A, the efficiency of deriving CTFR-bESCs is evaluated using different plating methods (whole blastocyst, mechanical isolation of inner cell mass [ICM], and immunosurgery-derived ICM) and various embryo sources (in vitro maturation [IVM]-in vitro fertilization [IVF], ovum pick-up [OPU]-IVF, somatic cell nuclear transfer [SCNT], and Holstein and Jersey breeds). The derivation efficiency is measured by calculating the percentage of blastocysts that successfully produce a stable CTFR-bESC line at the third passage (P3) in relation to the total number of embryos seeded using each method. Part B presents a schematic diagram illustrating the strategy of utilizing CTFR-bESCs for genomic selection. This approach aims to produce animals with superior genetic value through a highly efficient process involving CTFR-bESC derivation and somatic cell nuclear transfer (NT). The diagram demonstrates the potential of using CTFR-bESCs to select desirable genetic traits and generate animals with enhanced genetic characteristics. Part C highlights that CTFR-bESCs generated from different sources can serve as nuclear donors for cloning. This suggests that CTFR-bESCs derived from various embryo sources can be utilized in the cloning process to produce genetically identical copies of an organism. Reprinted from with [87] permission from the PNAS

**Fig. 6:**
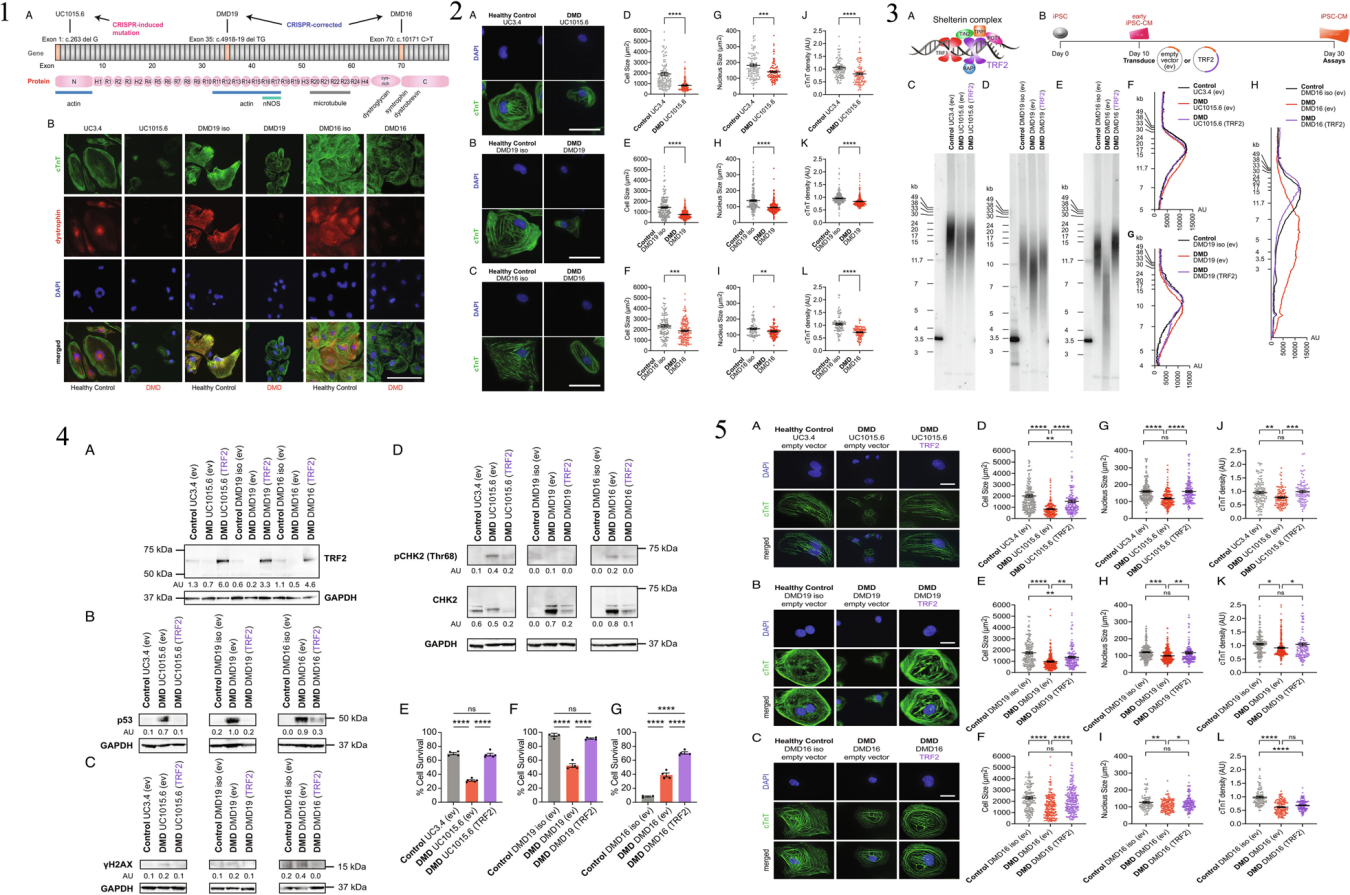
**1** The process of generating cardiomyocytes from iPSCs lacking the dystrophin gene. The figure consists of two parts. In Part A, a diagram depicts the dystrophin gene and the specific mutations found in the cell lines. The UC1015.6 line has a CRISPR-induced mutation that leads to the production of a truncated dystrophin protein without the N terminus. The DMD19 and DMD16 lines are derived from patients and possess nonsense mutations. In Part B, immunostaining is performed on day 30 iPSC-derived cardiomyocytes (iPSC-CMs) to visualize the presence of cTnT (cardiac troponin T) and dystrophin. The UC lines are stained using the MANEX1A antibody, which detects the N terminus of dystrophin. On the other hand, the DMD19 and DMD16 lines are stained using the ab15277 antibody, which recognizes the C terminus of dystrophin. Nuclei are marked in blue using DAPI. The scale bar represents a length of 100 µm. **2** The observed deficiencies in cell size, nuclear size, and sarcomere density of DMD iPSC-CMs (induced pluripotent stem cell-derived cardiomyocytes) on the 30th day of the differentiation process. The figure includes images of immunostaining for cTnT (cardiac troponin T) and DAPI (4',6-diamidino-2-phenylindole) staining for nuclei, comparing UC3.4 and UC1015.6 iPSC-CMs (A), DMD19 iso and DMD19 iPSC-CMs (B), and DMD16 iso and DMD16 iPSC-CMs (C). The scale bar in the images represents a length of 50 µm. Additionally, the figure presents the cell area measurements for UC3.4 and UC1015.6 iPSC-CMs (Figure D), DMD19 iso and DMD19 iPSC-CMs (Figure E), and DMD16 iso and DMD16 iPSC-CMs (F). The nuclear size measurements are provided for UC3.4 and UC1015.6 iPSC-CMs (Figure G), DMD19 iso and DMD19 iPSC-CMs (Figure H), and DMD16 iso and DMD16 iPSC-CMs (I). Furthermore, the figure displays the sarcomere density, quantified by the cTnT signal relative to the cell area, for UC3.4 and UC1015.6 iPSC-CMs (J), DMD19 iso and DMD19 iPSC-CMs (K), and DMD16 iso and DMD16 iPSC-CMs (L). The data were collected from three differentiation experiments, involving a total of 97 to 205 cells. **3** The effect of TRF2 overexpression on rescuing telomere attrition. The shelterin complex, consisting of six subunits, includes TRF1 and TRF2, which directly bind to telomere sequences. To investigate the impact of TRF2, cardiomyocytes were differentiated from iPSCs and transduced with either an empty retroviral vector without an open reading frame (ev) or TRF2 on day 10. Assays were conducted on day 30 of differentiation. Southern blot analysis was performed on telomere restriction fragments of iPSC-derived cardiomyocytes from UC, DMD19, and DMD16. The signal distribution of telomere lengths from the Southern blots is represented in arbitrary units (AU) for UC iPSC-CMs, DMD19 iPSC-CMs, and DMD16 iPSC-CMs. **4** The impact of TRF2 on the DNA damage response and cell survival. The experiment involved transducing cells with either ev (control) or TRF2 on day 10 and assessing them on day 30. The figure presents several Western blot analyses and survival percentages for different cell types. (A) TRF2 levels were analyzed using Western blot, with glyceraldehyde-3-phosphate dehydrogenase (GAPDH) as a loading control. TRF2 signal was normalized to the GAPDH signal and measured in arbitrary units (AU). The expected sizes for TRF2 and GAPDH are 65 kDa and 35 kDa, respectively. (B) Western blot analysis of P53 levels normalized to the GAPDH signal in AU. The expected size for P53 is 50 kDa. (C) Western blot analysis of gH2AX levels normalized to the GAPDH signal in AU. The expected size for gH2AX is 17 kDa. (D) Western blot analysis of CHK2 phosphorylated at threonine 68 (phosphor-CHK2) and total CHK2. The signals were normalized to the GAPDH signal in AU. The expected size for phosphor-CHK2 is 62 kDa. (E), (F), and (G) show the percentage of cells that survived on day 40 compared to day 30 of differentiation for UC iPSC-CMs, DMD19 iPSC-CMs, and DMD16 iPSC-CMs, respectively. Survival percentages were determined based on three to five differentiation experiments, with cell numbers ranging from 375 to 12,036 on day 30. **5** The effects of TRF2 on various cellular characteristics, including cell size, nuclear size, and sarcomere density. The experiment involved transducing cells with either the control vector (ev) or TRF2 on day 10 and assessing them on day 30. The images (A), (B), and (C) show immunostaining for cTnT and DAPI staining for nuclei in UC iPSC-CMs, DMD19 iPSC-CMs, and DMD16 iPSC-CMs, respectively. The scale bar represents a length of 20 µm. The area of cells in (D), (E), and (F) represents UC iPSC-CMs, DMD19 iPSC-CMs, and DMD16 iPSC-CMs, respectively. The nuclear size in (G), (H), and (I) corresponds to UC iPSC-CMs, DMD19 iPSC-CMs, and DMD16 iPSC-CMs, respectively. The sarcomere density, indicated by the cTnT signal over the cell area, is depicted in (J), (K), and (L) for UC iPSC-CMs, DMD19 iPSC-CMs, and DMD16 iPSC-CMs, respectively. The cells were evaluated based on three differentiation experiments, with a total of 90 to 230 cells analyzed. Reprinted from [91] with permission from the PNAS

Pluripotency is a defining characteristic of iPSCs that has revolutionized the field of regenerative medicine. Pluripotent cells have the remarkable ability to differentiate into cells of all three germ layers: ectoderm, endoderm, and mesoderm. This unique property allows iPSCs to give rise to various cell types found in the human body, making them highly valuable for therapeutic applications, disease modeling, and tissue engineering [85]. In this article, we will explore the concept of pluripotency, its significance in iPSC research, and the potential it holds for advancing regenerative medicine. Pluripotency refers to the developmental potential of a cell to give rise to multiple cell lineages. It is characterized by the capacity to differentiate into cells of all three germ layers, which are the precursors of different tissues and organs in the body. Pluripotent cells are similar to the inner cell mass of the blastocyst, a stage of early embryonic development [85, 86]. In a recent study conducted by Bogliotti et al. (2023), the researchers focused on the efficient derivation of stable pluripotent bovine embryonic stem cells (bESCs) from bovine blastocysts. In a recent study conducted by Bogliotti et al. (2023), the primary focus was on achieving the efficient generation of robust pluripotent bovine embryonic stem cells (bESCs) from bovine blastocysts. The primary objective of their research was to tackle the challenge of establishing stable lines of bESCs, which hold great importance for a wide range of applications in genomics, genome engineering, and disease modeling. To optimize the derivation process, the research team employed a culture system that incorporated fibroblast growth factor 2 (FGF2) and an inhibitor of the Wnt-signaling pathway. Remarkably, they successfully produced pluripotent bESCs with consistent characteristics in terms of morphology, transcriptome, karyotype, population-doubling rate, expression of pluripotency marker genes, and epigenetic features. Notably, the study underscored the pivotal role of iPSCs within its context. Furthermore, the researchers emphasized the potential applications of the obtained bESCs, particularly in genomic selection, and the figures presented in the research yielded valuable insights [87]. Figure 5 illustrated the process and characterization of CTFR-bESCs, highlighting the significance of cystic fibrosis transmembrane conductance regulator (CTFR) in bESC derivation. Figure 5 depicted the distribution of histone methylation patterns in CTFR-bESCs, emphasizing the epigenetic features of these cells. Figure 5 presented molecular characteristics of CTFR-bESCs, shedding light on their genetic and transcriptional profiles. Finally, Fig. 5 showcased the various potential applications of CTFR-bESCs, specifically their role in genomic selection. Collectively, this recent study provides valuable insights into the efficient derivation of stable bESCs and highlights the significant role of iPSCs, as well as the potential applications of CTFR-bESCs in genomic selection. ESCs derived from the inner cell mass are naturally pluripotent. However, the groundbreaking discovery by Shinya Yamanaka in 2006 demonstrated that adult somatic cells can also be reprogrammed to a pluripotent state, resulting in the generation of iPSCs [88]. The induction of pluripotency involves reprogramming adult somatic cells back to an embryonic-like state. This process requires the introduction of specific transcription factors, known as the Yamanaka factors, into the somatic cells. These factors include Oct4, Sox2, Klf4, and c-Myc. The Yamanaka factors work together to activate pluripotency-associated genes and silence lineage-specific genes, effectively resetting the cell to a pluripotent state [89]. The discovery of iPSCs opened up new avenues for regenerative medicine, as it bypassed the ethical concerns associated with the use of human embryos and offered the potential for personalized cell therapies [7]. Pluripotency is a crucial characteristic of iPSCs that distinguishes them from other cell types. It provides researchers with an abundant and ethically sound source of pluripotent cells for various applications. iPSCs can be derived from patient-specific somatic cells, allowing for the generation of autologous pluripotent cells. This personalized approach overcomes the challenges of immune rejection often faced in transplantation therapies. Moreover, the pluripotent nature of iPSCs enables their differentiation into specific cell types relevant to regenerative medicine [1, 90]. Through controlled differentiation protocols, iPSCs can be directed to become cardiomyocytes, neurons, hepatocytes, or any other desired cell type. This ability opens up possibilities for tissue engineering, where iPSC-derived cells can be used to replace damaged or dysfunctional cells in various organs [23]. A recent study conducted by Eguchi et al. (2023) investigated the study delved into the impact of telomere shortening in cardiomyocytes affected by Duchenne muscular dystrophy (DMD). Furthermore, the research explored the feasibility of preserving telomeres as a potential therapeutic approach for addressing this condition [91]. The researchers utilized iPSCs derived from DMD patients to generate cardiomyocytes. In Fig. 6, the authors illustrated the differentiation process of iPSCs lacking the dystrophin gene into cardiomyocytes. The study compared DMD iPSC-derived cardiomyocytes (DMD iPSC-CMs) to control cells on day 30 of differentiation, as shown in Fig. 6. The DMD iPSC-CMs exhibited reduced cell size, nuclear size, and sarcomere density compared to the control cells. To address telomere attrition, the researchers focused on the telomeric repeat-binding factor 2 (TRF2), a key component of the shelterin complex. In Fig. 6, the study summarized the effects of TRF2 overexpression on preventing telomere attrition in DMD iPSC-CMs. The results showed that TRF2 expression rescued the deficiencies in cell size and sarcomere density. To assess telomere length, the researchers employed a bioengineered platform for calcium imaging and performed Southern blots of telomere restriction fragments. The study also investigated the impact of TRF2 on the DNA damage response and cell survival, as depicted in Fig. 6. The findings indicated that preventing telomere attrition through TRF2 overexpression ameliorated the activation of the DNA damage response and reduced premature cell death. Importantly, Fig. 6 demonstrated the effects of TRF2 on various cellular characteristics, including cell morphology, telomere length, activation of DNA damage response, and cell viability. Overall, these findings highlight the crucial role of iPSCs in studying disease mechanisms and therapeutic interventions, and suggest that preserving telomere length through TRF2 may hold promise for treating DMD-associated cardiac failure.

##### Self-renewal

Self-renewal is a fundamental property of stem cells that enables them to proliferate and maintain their undifferentiated state [15]. This property is critical for the regenerative potential of stem cells as it ensures a constant supply of cells for tissue repair and regeneration [38]. Self-renewal is a complex process that involves the regulation of multiple signaling pathways and gene networks. Understanding the mechanisms underlying self-renewal is essential for harnessing the therapeutic potential of stem cells. Self-renewal refers to the ability of stem cells to divide symmetrically or asymmetrically to produce daughter cells that retain their stem cell identity [92]. This process ensures a continuous supply of undifferentiated cells that can differentiate into specialized cell types. The balance between self-renewal and differentiation is tightly regulated to maintain tissue homeostasis and prevent the depletion of stem cell pools. Self-renewal is a fundamental characteristic of stem cells that underpins their regenerative potential [93]. The ability of stem cells to continuously proliferate while maintaining their undifferentiated state is crucial for tissue repair, transplantation, and disease modeling. The self-renewal capacity of stem cells, combined with their differentiation potential, makes them valuable tools for regenerative medicine, personalized therapies, and drug discovery [94]. Despite the challenges and complexities associated with self-renewal, ongoing research is steadily advancing our understanding of stem cell biology and paving the way for innovative applications in regenerative medicine. With further advancements in the field, the harnessing of self-renewal in stem cells holds great promise for revolutionizing healthcare and improving the treatment options available for a wide range of diseases and injuries [95].

##### Self-renewal in ESCs

ESCs are derived from the inner cell mass of blastocysts and are characterized by their pluripotency and unlimited self-renewal capacity. The self-renewal of ESCs is controlled by multiple signaling pathways, including the Wnt, FGF, and TGF-beta pathways, which interact with transcription factors such as Oct4, Sox2, and Nanog. These factors form a regulatory network that maintains the pluripotent state of ESCs and suppresses differentiation cues [96]. Adult stem cells, also known as somatic stem cells, are found in various tissues throughout the body and are responsible for tissue repair and regeneration. Adult stem cells have a more restricted differentiation potential than ESCs and are typically committed to a specific lineage. The self-renewal of adult stem cells is regulated by both intrinsic and extrinsic factors, including growth factors, cytokines, and niche microenvironments. One of the key intrinsic factors that regulate self-renewal in adult stem cells is the transcription factor Sox2 [97]. Sox2 is required for the maintenance of neural stem cells, hematopoietic stem cells, and mesenchymal stem cells. In addition, the Notch signaling pathway has been shown to play a critical role in the self-renewal of adult stem cells in various tissues, including the intestinal epithelium and the skin [19].

##### Challenges in self-renewal

While self-renewal is a critical property of stem cells, it can also lead to the accumulation of genetic and epigenetic changes that increase the risk of cancer and other diseases. The regulation of self-renewal is therefore a delicate balance that must be tightly controlled to prevent the over proliferation of stem cells. Another challenge in self-renewal is the loss of potency that can occur during long-term culture. As stem cells divide and differentiate, they may lose their ability to generate certain cell types or become more prone to differentiation into specific lineages. This loss of potency can limit the therapeutic potential of stem cells and must be addressed through rigorous quality control measures and optimization of culture conditions [25].

##### Applications of self-renewal in regenerative medicine

The self-renewal capacity of stem cells is critical for the development of regenerative therapies that aim to replace damaged or diseased tissues. By harnessing the regenerative potential of stem cells, researchers hope to develop new treatments for a wide range of diseases, including cardiovascular disease, diabetes, and neurodegenerative disorders. One of the most promising applications of self-renewal is in tissue engineering [19]. By combining stem cells with biomaterials and growth factors, researchers aim to generate functional tissues and organs that can be transplanted into patients. The ability of stem cells to self-renew ensures a sufficient supply of cells for tissue engineering, allowing for the creation of large-scale, complex tissues. For example, MSCs have shown promising self-renewal capacity and can differentiate into various cell types, making them valuable for engineering bone, cartilage, and other connective tissues [98]. In addition to tissue engineering, self-renewal plays a crucial role in the field of regenerative medicine by enabling the expansion of stem cell populations for therapeutic purposes. For instance, hematopoietic stem cells (HSCs) have the ability to self-renew and differentiate into different blood cell types. HSC transplantation has been successfully used to treat various blood disorders, such as leukemia and aplastic anemia, where the self-renewal capacity of HSCs ensures a sustained production of healthy blood cells. The concept of self-renewal is also integral to the development of personalized medicine [99]. By isolating patient-specific stem cells and inducing their self-renewal, it is possible to generate a renewable source of cells for transplantation back into the same individual. This approach minimizes the risk of rejection and graft-versus-host disease, making autologous stem cell transplantation a promising option for personalized therapies. Furthermore, the self-renewal potential of stem cells holds significant implications for drug discovery and toxicology studies. Stem cells, including iPSCs, can be used to generate disease-specific or genetically modified cell lines that recapitulate the characteristics of certain diseases or specific patient populations [19]. This allows for the screening of potential drug candidates and evaluation of their efficacy and safety profiles, thereby facilitating the development of more targeted and personalized therapeutics [22].

#### Genetic stability

In the field of regenerative medicine, the genetic stability of iPSCs is a crucial aspect to consider. iPSCs, derived by reprogramming adult somatic cells, hold great promise for tissue engineering, disease modeling, and therapeutic applications. However, it is essential to ensure that the reprogramming process and subsequent culture conditions do not introduce genetic abnormalities or mutations that could compromise the safety and efficacy of iPSC-based therapies [1, 7]. This article explores the significance of genetic stability in iPSCs and the strategies employed to ensure the reliability of these cells for regenerative medicine purposes. Genetic stability refers to the preservation of the genetic information of iPSCs throughout the reprogramming process and subsequent cell culture. Any alterations or mutations in the genomic DNA of iPSCs can have profound implications for their clinical use. Genetic instability can lead to unintended consequences, such as aberrant differentiation potential, compromised functionality, or even the development of tumorigenic properties. Therefore, it is crucial to ensure the genetic integrity of iPSCs to maximize their potential in regenerative medicine [59].

##### Reprogramming methods

The choice of reprogramming methods can significantly influence the genetic stability of iPSCs. Various techniques, such as viral integration, non-integrating methods, and episomal vectors, have been used to deliver the reprogramming factors. Viral integration, although efficient, carries the risk of insertional mutagenesis. Non-integrating methods, such as mRNA or protein-based delivery, minimize the risk of genetic alterations. Researchers have focused on developing safer reprogramming approaches to enhance the genetic stability of iPSCs [100].

##### Quality control measures

Rigorous quality control measures are essential to assess the genetic stability of iPSCs. Whole-genome sequencing (WGS) and karyotyping are commonly employed techniques to analyze the entire genome and detect any chromosomal abnormalities or genetic mutations. WGS allows for the identification of single nucleotide variants (SNVs), insertions, deletions, copy number variations (CNVs), and structural variations (SVs). Karyotyping, on the other hand, enables the examination of chromosome number and structure. These analyses help identify any genetic variations and ensure the genetic stability of iPSCs [101].

##### Epigenetic reprogramming

Epigenetic modifications play a vital role in cellular identity and function. During the reprogramming process, the epigenetic landscape of somatic cells is reset to an embryonic-like state. However, incomplete or aberrant epigenetic reprogramming can lead to genetic instability in iPSCs. Techniques such as DNA methylation profiling and histone modification analysis are used to assess the epigenetic status of iPSCs. Proper epigenetic reprogramming is crucial to maintain the genetic stability and pluripotency of iPSCs [102].

##### Long-term culturing conditions

Maintaining iPSCs in culture for extended periods can increase the risk of genetic instability. Factors such as culture media composition, substrate coating, and passaging methods can influence the genetic stability of iPSCs. It is important to optimize culture conditions to minimize the accumulation of genetic alterations over time. Researchers are continually exploring novel culture systems, such as feeder-free culture or defined media formulations, to enhance the genetic stability of iPSCs during long-term culturing [103].

##### Clonal expansion and characterization

Clonal expansion of iPSCs involves isolating and expanding individual iPSC colonies to ensure clonality and genetic homogeneity. This step helps identify and eliminate any genetically unstable iPSC lines. Furthermore, characterization of iPSCs at the molecular and functional level is essential to assess their genetic stability. Techniques such as immunocytochemistry, flow cytometry, and gene expression analysis can be employed to evaluate the pluripotency markers and differentiation potential of iPSCs. Additionally, functional assays can be performed to confirm their ability to differentiate into various cell lineages. Comprehensive characterization ensures that only genetically stable and functionally competent iPSC lines are selected for downstream applications [104].

##### Genome editing tools

**Fig. 7:**
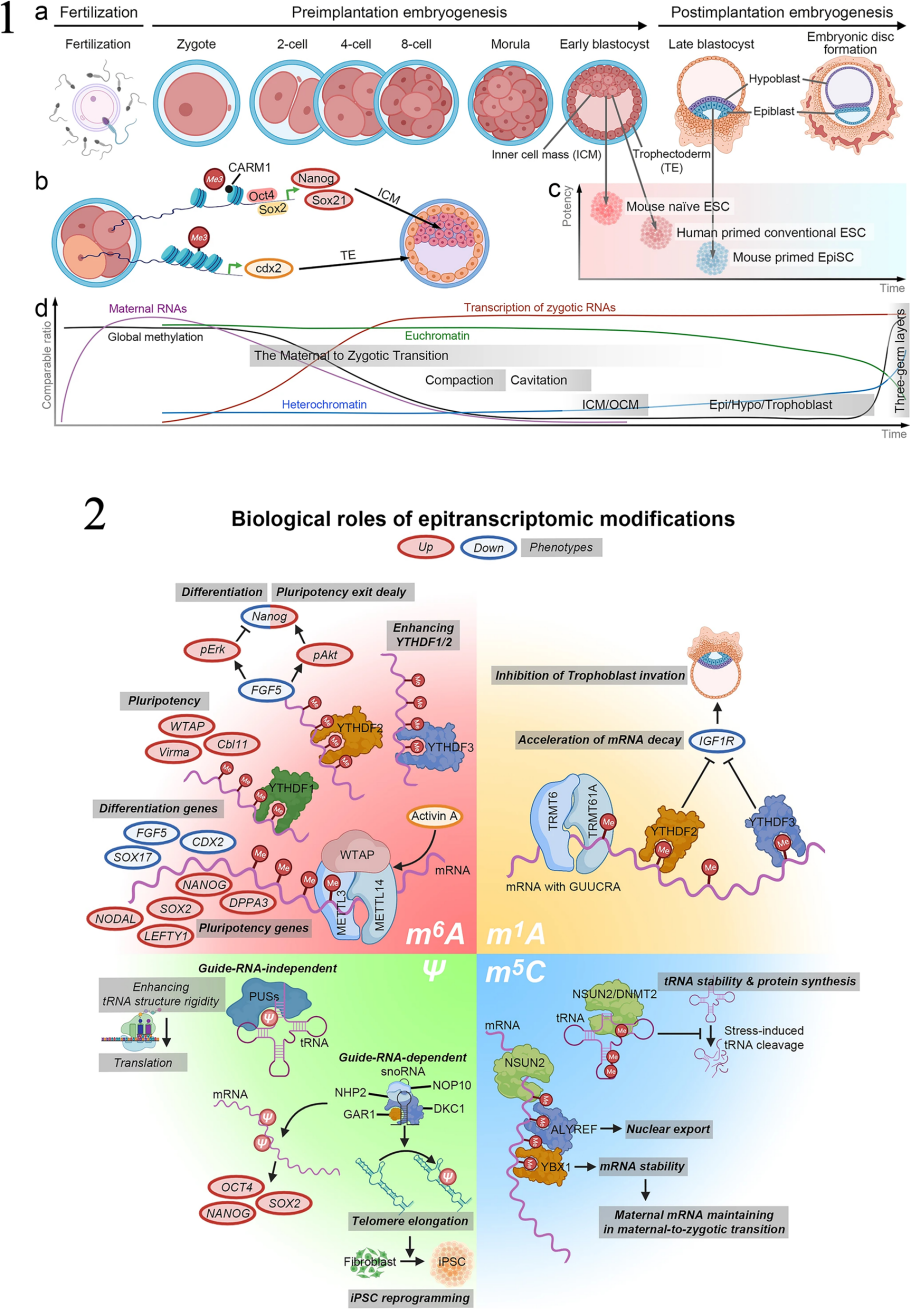
**1** The progression of stem cell development, focusing on various aspects. In panel a, it depicts the morphological transformations that occur at different stages of stem cell development. Panel b highlights the molecular processes involved in the acquisition of cellular diversity. Panel c showcases the pluripotent nature of stem cells obtained at different developmental phases. Lastly, panel d provides an overview of the molecular characteristics observed throughout the course of stem cell development. **2** The control of different characteristics in pluripotent stem cells through epitranscriptomic modifications. The regulatory effects of m6A (indicated in red), m1A (in yellow), pseudouridine (in green), and m5C (in blue) modifications are linked to specific biological traits exhibited by pluripotent stem cells. Reprinted from [106] with permission from the Springer Nature

Advances in genome editing technologies, such as CRISPR-Cas9, provide valuable tools to correct genetic abnormalities or introduce specific genetic modifications in iPSCs. This approach can be used to repair genetic mutations or eliminate unwanted genomic variations. Genome editing serves as a powerful strategy to enhance the genetic stability of iPSCs and ensure their suitability for regenerative medicine applications [105]. Recent studies have provided valuable insights into the epitranscriptomic control of pluripotent stem cell fate. In a groundbreaking investigation conducted the researchers delved into the influence of RNA modifications on the destiny determination of iPSCs [106]. Utilizing advanced high-throughput sequencing techniques, they discerned dynamic alterations in RNA modifications throughout the reprogramming process, where somatic cells were transformed into iPSCs. Their findings highlighted the critical importance of specific epitranscriptomic modifications, such as N6-methyladenosine (m6A) and N1-methyladenosine (m1A), in both acquiring and sustaining pluripotency. Furthermore, the researchers demonstrated that the manipulation of enzymes responsible for RNA modifications could enhance the efficiency of iPSC generation and steer the differentiation potential of iPSCs towards particular lineages. These results underscore the pivotal role of the epitranscriptome in governing iPSC fate and accentuate the potential utilization of epitranscriptomic modifications as targets for enhancing iPSC-based therapies. Additionally, Fig. 7 illustrates these findings, while Fig. 7 visually represents the diverse effects of various epitranscriptomic modifications on pluripotent stem cell characteristics, highlighting the intricate regulatory functions of RNA modifications in shaping the fate of pluripotent stem cells.

#### Immune compatibility

Regenerative medicine is a rapidly evolving field that aims to restore, replace, or regenerate damaged tissues and organs. One of the key challenges in this field is to develop therapeutic strategies that can avoid immune rejection and improve the long-term survival of transplanted cells and tissues. iPSCs have emerged as a promising tool in this regard, as they offer the potential to generate patient-specific cells that are immunologically compatible with the host [30]. While iPSCs offer great potential for immune compatibility in regenerative medicine, there are still challenges that need to be addressed. One major concern is the potential for immune rejection of iPSC-derived cells due to residual expression of immunogenic antigens or incomplete reprogramming. It is crucial to thoroughly characterize iPSC lines to ensure their safety and immunological compatibility before clinical application [25]. Another challenge is the long-term survival and functionality of iPSC-derived cells in the host environment. Even if immune rejection is minimized, the transplanted cells may still face challenges in integrating into the host tissue, establishing functional connections, and maintaining their desired phenotype [107]. Further research is needed to optimize differentiation protocols, enhance cell maturation, and improve the engraftment of iPSC-derived cells for successful long-term outcomes. Additionally, ethical considerations surrounding the use of iPSCs in regenerative medicine should not be overlooked. Issues such as the informed consent of donors, privacy of genetic information, and the potential for commercialization of iPSC-based therapies need to be carefully addressed to ensure responsible and ethical implementation [108].

##### Immune compatibility of iPSCs

The iPSCs possess several advantages over other sources of stem cells in terms of immune compatibility. Unlike ESCs, which are derived from embryos and may elicit an immune response when transplanted, iPSCs can be generated from adult somatic cells, such as skin fibroblasts or blood cells. This means that iPSCs can be produced from the same individual who will receive the therapy, minimizing the risk of immune rejection [25].

##### Autologous iPSCs

The use of autologous iPSCs, generated from a patient’s own cells, offers the greatest potential for immune compatibility. As the iPSCs are genetically identical to the donor, there is no risk of rejection or graft-versus-host disease. This approach is particularly useful in the treatment of diseases such as Parkinson’s disease, diabetes, or spinal cord injuries, where the goal is to replace lost or damaged cells with healthy ones. In these cases, iPSCs can be differentiated into the desired cell type, such as dopaminergic neurons or pancreatic beta cells, before transplantation. However, the generation of autologous iPSCs is not always feasible or practical. For example, patients with genetic disorders may have mutations present in all of their cells, making the generation of disease-free iPSCs difficult. Additionally, patients with advanced or widespread disease may not have a sufficient number of healthy cells to generate iPSCs. In these cases, allogeneic iPSCs, generated from a donor’s cells, may be utilized [109].

##### Allogeneic iPSCs

The use of allogeneic iPSCs raises the possibility of immune rejection, as the cells are genetically different from the host. However, several approaches are being explored to overcome this challenge. One approach is to use gene editing technologies, such as CRISPR/Cas9, to introduce genetic modifications into the iPSCs that reduce their immunogenicity. For example, researchers can delete or downregulate genes that code for major histocompatibility complex (MHC) molecules, which are responsible for presenting antigens to T cells and triggering an immune response. By reducing MHC expression, iPSCs may be able to evade detection by the host immune system. Another approach is to use immunosuppressive drugs, such as cyclosporine or tacrolimus, to dampen the host immune response. However, these drugs can have significant side effects and increase the risk of infection or malignancy. Therefore, alternative strategies that promote immune tolerance and reduce the need for immunosuppression are being investigated [110].

##### Promoting immune tolerance

One approach to promoting immune tolerance is to generate iPSC-derived cells that express immune-modulatory molecules, such as indoleamine 2,3-dioxygenase (IDO), programmed death-ligand 1 (PD-L1), or galectin-1. These molecules can inhibit T cell activation and promote the generation of regulatory T cells, which suppress immune responses. By incorporating these molecules into iPSC-derived cells, researchers aim to create a “tolerogenic” environment that can induce immune tolerance and reduce the risk of rejection [111].

### Potential applications in regenerative medicine

The iPSCs can be differentiated into specific cell types relevant to tissue repair and regeneration. For instance, they can be directed to differentiate into cardiomyocytes for cardiac tissue regeneration, hepatocytes for liver tissue engineering, or neurons for the treatment of neurological disorders. The ability to generate patient-specific cells holds great promise for developing customized therapies and overcoming the limitations associated with organ transplantation. The iPSCs provide a valuable platform for studying the mechanisms underlying various diseases. By reprogramming cells from patients with genetic disorders or complex diseases, researchers can generate disease-specific iPSC lines that recapitulate the pathology in a controlled laboratory setting [19]. These disease models allow for a better understanding of disease progression, identification of novel therapeutic targets, and screening of potential drugs. Traditional drug development processes rely heavily on animal models and can be time-consuming and costly. iPSCs offer an alternative approach by providing human-specific cell models for drug screening and toxicity testing. iPSC-derived cells, such as cardiomyocytes or hepatocytes, can be utilized to evaluate the efficacy and safety of drug candidates, reducing the need for animal testing and improving the accuracy of preclinical studies. In certain degenerative diseases, such as Parkinson’s disease or diabetes, iPSCs hold the potential to replace damaged or dysfunctional cells [23]. By differentiating iPSCs into the desired cell type, such as dopaminergic neurons or pancreatic beta cells, researchers aim to restore lost function and alleviate disease symptoms. However, challenges related to the scalability, maturation, and long-term safety of iPSC- derived cells need to be addressed before widespread clinical application [7].

### Advancements in the field of iPSC research

Over the past decade, the field of iPSC research has seen significant advancements and progress. iPSCs are a type of stem cell that are created by reprogramming adult cells to a pluripotent state, meaning they have the potential to develop into any type of cell in the body. This technology has opened up new avenues for regenerative medicine, disease modeling, drug discovery, and personalized medicine [1]. One of the most significant advancements in iPSC research has been the development of more efficient and reliable methods for reprogramming adult cells. Early methods of reprogramming involved the introduction of transcription factors into cells using viruses, which posed a risk of mutations and potential tumorigenesis. However, recent advances have utilized non-viral methods such as mRNA, microRNA, and small molecules, which are safer and more efficient [112]. These methods have also led to the production of high-quality iPSCs with improved differentiation potential and reduced genomic abnormalities. Another area of advancement in iPSC research is the development of more sophisticated techniques for manipulating and controlling iPSC differentiation. Scientists can now direct iPSCs to differentiate into specific cell types, such as neurons, cardiac cells, and pancreatic cells, using specific growth factors, signaling molecules, and genetic manipulation [19]. This has led to the creation of more complex in vitro models of human disease, allowing for the study of disease mechanisms and drug screening. In addition, iPSC research has made significant strides in the field of personalized medicine. iPSCs can be generated from a patient’s own cells and then differentiated into specific cell types, allowing for the creation of personalized disease models and drug screening platforms. This has the potential to revolutionize drug discovery, as it allows for the identification of patient-specific drug targets and the development of personalized treatment plans. Another major advancement in the field of iPSC research is the use of gene editing techniques such as CRISPR-Cas9 to manipulate the genome of iPSCs [7]. This has allowed for the creation of disease-specific iPSC lines with specific genetic mutations, which can be used to study disease mechanisms and test potential therapies. Gene editing also holds potential for the development of gene therapies, which could treat genetic diseases by correcting mutations in iPSCs and then differentiating them into the affected cell type. Advancements in iPSC research have also led to new approaches in cancer research and therapy [1]. iPSCs can be used to model cancer development and progression, allowing for the identification of genetic and epigenetic changes that drive tumor growth. Additionally, iPSCs can be used to develop personalized cancer treatments, such as chimeric antigen receptor (CAR) T cell therapy. CAR T cell therapy involves reprogramming a patient’s own T cells using iPSC technology, allowing them to specifically target and kill cancer cells [113]. Finally, advancements in iPSC research have raised important ethical and legal considerations. The creation and use of iPSCs raises questions about ownership, consent, and the potential for genetic manipulation and designer babies. Additionally, the use of iPSCs in drug screening and disease modeling could lead to the exploitation of vulnerable populations, such as those with rare genetic diseases [22].

## iPSCs in cancer therapy

One of the key advantages of iPSCs in cancer therapy is their ability to serve as models for studying cancer biology [1]. By reprogramming somatic cells from cancer patients into iPSCs, researchers can generate patient-specific cell lines that retain the genetic and epigenetic characteristics of the original tumor [2]. These iPSC-derived cancer cells can be differentiated into different cell types and used to study the mechanisms of cancer development, progression, and response to treatment [3]. This approach provides a unique opportunity to investigate individualized responses to various therapies and develop personalized treatment strategies [4].

### iPSCs as a source of cancer cells for research and drug screening

The iPSCs have demonstrated great potential as a valuable tool for studying cancer biology and developing novel therapies [2]. One significant application of iPSCs in cancer research is their use as a source of cancer cells for in vitro studies and drug screening [4]. By reprogramming somatic cells from cancer patients, researchers can generate iPSC lines that harbor the genetic and epigenetic alterations characteristic of the individual’s cancer [1]. These patient-specific iPSCs can be further differentiated into various cancer cell types, allowing for the creation of in vitro models that closely resemble the patient’s tumor [1]. This enables researchers to study the molecular mechanisms underlying cancer initiation, progression, and response to treatment. By comparing iPSC-derived cancer cells with healthy cells from the same individual, scientists can identify key genetic and epigenetic changes that contribute to cancer development [5]. Moreover, iPSC-derived cancer cells provide a platform for drug screening and testing. Researchers can expose these cells to different therapeutic agents to evaluate their efficacy and selectivity against the specific cancer type [5]. iPSC-derived models allow for personalized medicine approaches, where drugs can be screened on patient-specific cancer cells to identify the most effective treatment options [6]. This personalized approach has the potential to revolutionize cancer therapy by tailoring treatments to individual patients, improving treatment outcomes, and reducing adverse effects [7]. Table 3 provides an overview of the utilization of iPSCs as a valuable source of cancer cells for research and drug screening purposes.

**Table 3:** iPSCs as a source of cancer cells for research and drug screening

Cancer Type	iPSC Line used	In vitro Model of Cancer Cells	Name of Drug	Comparison with traditional cancer cell lines	Mechanism	References
Breast cancer	BC1 iPSCs	Triple-negative breast cancer	Olaparib	Higher genetic diversity, greater resemblance to in vivo tumors	PARP inhibitor, induces DNA damage	[114–116]
Lung cancer	H1975 iPSCs	Non-small cell lung cancer	Erlotinib	More accurate representation of patient-specific tumors	EGFR inhibitor, blocks signal transduction	[117]
Leukemia	CML-iPSCs	Chronic myeloid leukemia	Imatinib	Reflects heterogeneity of disease, allows for personalized therapy	BCR-ABL inhibitor, inhibits tyrosine kinase activity	[118, 119]
Prostate cancer	PC-iPSCs	Androgen receptor-positive prostate cancer	Enzalutamide	More relevant for studying tumor evolution, allows for drug resistance testing	Androgen receptor inhibitor, blocks signaling pathway	[120]
Melanoma	Mel-iPSCs	Metastatic melanoma	Ipilimumab	Offers insight into immunotherapy resistance, allows for drug screening	CTLA-4 antibody, enhances T cell response against tumor cells	[121]
Pancreatic cancer	iPSCs derived from cancerous tissue	Pancreatic ductal adenocarcinoma	Gemcitabine	Provides more accurate drug sensitivity testing, facilitates drug discovery	Nucleoside analog, inhibits DNA replication	[122]
Ovarian cancer	Ov-iPSCs	High-grade serous ovarian carcinoma	Carboplatin	Provides patient-specific drug response data	Platinum-based chemotherapy, DNA crosslinking	[123, 124]
Brain cancer	GBM-iPSCs	Glioblastoma multiforme	Temozolomide	Captures intratumoral heterogeneity, enables personalized therapy	Alkylating agent, DNA damage inducer	[125, 126]
Pancreatic cancer	PDAC-iPSCs	Pancreatic ductal adenocarcinoma	Nab-paclitaxel	Represents patient-specific responses to therapy	Microtubule inhibitor, disrupts mitosis	[127]
Lymphoma	Lymp-iPSCs	Diffuse large B-cell lymphoma	Rituximab	Captures tumor heterogeneity and response to immunotherapy	CD20 antibody, targets B-cell lymphoma cells	[128]
Gastric cancer	GC-iPSCs	Gastric adenocarcinoma	Trastuzumab	Enables personalized treatment based on HER2 expression	HER2 antibody, blocks HER2 signaling	[50]
Bladder cancer	Blad-iPSCs	Urothelial carcinoma	Pembrolizumab	Allows for evaluation of immune checkpoint inhibitors	PD-1 antibody, enhances immune response	[51]
Prostate cancer	PC-iPSCs	Androgen receptor-positive prostate cancer	Abiraterone	Represents patient-specific response to androgen deprivation therapy	Androgen synthesis inhibitor, blocks testosterone production	[52]
Pancreatic cancer	PDAC-iPSCs	Pancreatic ductal adenocarcinoma	FOLFIRINOX	Reflects patient-specific chemotherapy response	Combination chemotherapy (5-FU, irinotecan, oxaliplatin)	[53]
Brain cancer	GBM-iPSCs	Glioblastoma multiforme	Bevacizumab	Recapitulates anti-angiogenic therapy response	VEGF antibody, inhibits angiogenesis	[129]
Colorectal cancer	iPSCs derived from cancerous tissue	Colorectal adenocarcinoma	Cetuximab	Represents patient-specific response to targeted therapy	EGFR antibody, inhibits EGFR signaling	[130]
Lung cancer	NSCLC-iPSCs	Non-small cell lung cancer	Pembrolizumab	Enables assessment of immune checkpoint inhibitor efficacy	PD-1 antibody, enhances immune response	[131]
Leukemia	AML-iPSCs	Acute myeloid leukemia	Venetoclax	Represents patient-specific response to targeted therapy	BCL-2 inhibitor, promotes apoptosis	[132]
Pancreatic cancer	PDAC-iPSCs	Pancreatic ductal adenocarcinoma	Panobinostat	Represents patient-specific response to histone deacetylase inhibitors	HDAC inhibitor, alters gene expression and induces apoptosis	[133]
Breast cancer	BC-iPSCs	HER2-positive breast cancer	Trastuzumab emtansine	Reflects patient-specific response to antibody–drug conjugates	HER2-targeted antibody–drug conjugate, inhibits cell division	[134]
Prostate cancer	PC-iPSCs	Castration-resistant prostate cancer	Docetaxel	Represents patient-specific response to chemotherapy	Microtubule inhibitor, disrupts cell division	[135]
Liver cancer	Hep-iPSCs	Hepatocellular carcinoma	Lenvatinib	Better recapitulation of liver tumor angiogenesis	Multi-kinase inhibitor, inhibits VEGF signaling	[136]
Lung cancer	NSCLC-iPSCs	EGFR-mutated non-small cell lung cancer	Osimertinib	Represents patient-specific response to EGFR tyrosine kinase inhibitors	EGFR inhibitor, blocks mutant EGFR signaling	[137]
Colorectal cancer	CRC-iPSCs	KRAS-mutated colorectal cancer	Cetuximab	Reflects patient-specific response to anti-EGFR therapy	EGFR antibody, inhibits EGFR signaling	[130, 138]
Ovarian cancer	Ov-iPSCs	Epithelial ovarian cancer	Niraparib	Represents patient-specific response to PARP inhibitors	PARP inhibitor, disrupts DNA repair mechanisms	[139]
Melanoma	Mel-iPSCs	BRAF-mutated melanoma	Vemurafenib	Represents patient-specific response to BRAF inhibitors	BRAF inhibitor, blocks mutant BRAF signaling	[140, 141]
Breast cancer	BC-iPSCs	Luminal B breast cancer	Palbociclib	Enables investigation of CDK4/6 inhibitor response	CDK4/6 inhibitor, arrests cell cycle	[142]
Prostate cancer	PC-iPSCs	Neuroendocrine prostate cancer	Cabazitaxel	Represents patient-specific response to taxane chemotherapy	Taxane chemotherapeutic, disrupts microtubule function	[143]
Lung cancer	NSCLC-iPSCs	ALK-positive non-small cell lung cancer	Alectinib	Reflects patient-specific response to ALK inhibitors	ALK inhibitor, blocks ALK signaling	[144, 145]
Colorectal cancer	CRC-iPSCs	MSI-high colorectal cancer	Pembrolizumab	Enables evaluation of immune checkpoint blockade in MSI-high tumors	PD-1 antibody, enhances immune response	[146]
Pancreatic cancer	PDAC-iPSCs	Pancreatic ductal adenocarcinoma	Napabucasin	Represents patient-specific response to cancer stem cell inhibitors	Cancer stem cell inhibitor, targets cancer stem cells	[147]
Breast cancer	BC-iPSCs	HER2-negative breast cancer	Paclitaxel	Enables investigation of taxane chemotherapy response	Microtubule inhibitor, disrupts cell division	[148, 149]
Prostate cancer	PC-iPSCs	Metastatic castration-resistant prostate cancer	Radium-223	Reflects patient-specific response to targeted alpha therapy	Alpha particle emitter, targets bone metastases	[150]
Lung cancer	NSCLC-iPSCs	ROS1-positive non-small cell lung cancer	Crizotinib	Represents patient-specific response to ROS1 inhibitors	ROS1 inhibitor, blocks ROS1 signaling	[151]
Colorectal cancer	CRC-iPSCs	BRAF-mutated colorectal cancer	Encorafenib + Cetuximab	Reflects patient-specific response to combination therapy	BRAF inhibitor + EGFR antibody, dual pathway inhibition	[152, 153]
Liver cancer	Hep-iPSCs	Fibrolamellar hepatocellular carcinoma	Debio 1347	Represents patient-specific response to FGFR inhibitors	FGFR inhibitor, blocks FGFR signaling	[154]
Breast cancer	BC-iPSCs	Triple-positive breast cancer	Trastuzumab + Pertuzumab	Reflects patient-specific response to HER2-targeted combination therapy	Dual HER2 inhibition, blocks HER2 signaling	[155]
Prostate cancer	PC-iPSCs	Androgen receptor-negative prostate cancer	Cabozantinib	Enables investigation of MET inhibitor response	MET inhibitor, blocks MET signaling	[156]
Lung cancer	NSCLC-iPSCs	KRAS-mutated non-small cell lung cancer	Binimetinib	Represents patient-specific response to MEK inhibitors	MEK inhibitor, blocks MEK signaling	[157]
Colorectal cancer	CRC-iPSCs	Microsatellite-stable colorectal cancer	5-Fluorouracil + Oxaliplatin	Reflects patient-specific response to combination chemotherapy	Combination chemotherapy, disrupts DNA replication and cell division	[158]
Ovarian cancer	Ov-iPSCs	Endometrioid ovarian carcinoma	Carboplatin + Paclitaxel	Represents patient-specific response to standard chemotherapy	Combination chemotherapy, disrupts DNA replication and cell division	[159]
Liver cancer	Hep-iPSCs	Fibrolamellar hepatocellular carcinoma	Atezolizumab + Bevacizumab	Enables evaluation of immune checkpoint blockade and anti-angiogenic therapy	PD-L1 antibody + VEGF antibody, enhances immune response and inhibits angiogenesis	[160, 161]
Pancreatic cancer	PDAC-iPSCs	Pancreatic ductal adenocarcinoma	Gemcitabine + Abraxane	Represents patient-specific response to combination chemotherapy	Combination chemotherapy, disrupts DNA replication and cell division	[162]
Breast cancer	BC-iPSCs	Triple-negative breast cancer	Pembrolizumab	Enables evaluation of immune checkpoint blockade	PD-1 antibody, enhances immune response	[163, 164]
Colorectal cancer	CRC-iPSCs	APC-mutated colorectal cancer	Celecoxib	Represents patient-specific response to COX-2 inhibitors	COX-2 inhibitor, inhibits inflammation and cell proliferation	[165]
Ovarian cancer	Ov-iPSCs	Serous ovarian carcinoma	Olaparib	Enables investigation of PARP inhibitor response	PARP inhibitor, disrupts DNA repair mechanisms	[166]
Breast cancer	BC-iPSCs	Estrogen receptor-positive breast cancer	Fulvestrant	Enables investigation of estrogen receptor degradation	Selective estrogen receptor degrader, inhibits estrogen receptor signaling	[167]
Prostate cancer	PC-iPSCs	TMPRSS2-ERG fusion-positive prostate cancer	Enzalutamide + Apalutamide	Represents patient-specific response to dual androgen receptor inhibitors	Dual androgen receptor inhibitors, block androgen signaling	[168]
Lung cancer	NSCLC-iPSCs	ALK-positive non-small cell lung cancer	Ceritinib	Reflects patient-specific response to ALK inhibitors	ALK inhibitor, blocks ALK signaling	[169]
Colorectal cancer	CRC-iPSCs	CEA-positive colorectal cancer	Regorafenib	Represents patient-specific response to multi-kinase inhibitors	Multi-kinase inhibitor, inhibits angiogenesis and cell proliferation	[170–172]
Ovarian cancer	Ov-iPSCs	High-grade serous ovarian cancer	Topotecan	Enables investigation of topoisomerase I inhibitor response	Topoisomerase I inhibitor, disrupts DNA replication	[173]
Liver cancer	Hep-iPSCs	Hepatocellular carcinoma with TP53 mutation	Sorafenib + Vorinostat	Represents patient-specific response to combination targeted therapy	Multi-kinase inhibitor + HDAC inhibitor, inhibits angiogenesis and alters gene expression	[174]
Pancreatic cancer	PDAC-iPSCs	Pancreatic ductal adenocarcinoma	Ibrutinib	Represents patient-specific response to targeted therapy	Bruton's tyrosine kinase inhibitor, inhibits B-cell signaling	[175]
Breast cancer	BC-iPSCs	Basal-like breast cancer	Talazoparib	Enables investigation of PARP inhibitor response	PARP inhibitor, disrupts DNA repair mechanisms	[176, 177]
Prostate cancer	PC-iPSCs	AR-V7-positive castration-resistant prostate cancer	Darolutamide	Represents patient-specific response to androgen receptor antagonists	Androgen receptor antagonist, inhibits androgen receptor signaling	[143, 178]
Lung cancer	NSCLC-iPSCs	MET-amplified non-small cell lung cancer	Capmatinib	Reflects patient-specific response to MET inhibitors	MET inhibitor, blocks MET signaling	[179]
Colorectal cancer	CRC-iPSCs	BRAFV600E-mutated colorectal cancer	Encorafenib + Binimetinib	Represents patient-specific response to targeted combination therapy	BRAF inhibitor + MEK inhibitor, dual pathway inhibition	[180]
Ovarian cancer	Ov-iPSCs	Low-grade serous ovarian carcinoma	MEK162	Enables investigation of MEK inhibitor response	MEK inhibitor, blocks MEK signaling	[181]
Liver cancer	Hep-iPSCs	Hepatocellular carcinoma with AFP overexpression	Ramucirumab	Represents patient-specific response to anti-angiogenic therapy	VEGFR2 antibody, inhibits angiogenesis	[179]
Pancreatic cancer	PDAC-iPSCs	Pancreatic ductal adenocarcinoma	PEGPH20	Enables investigation of hyaluronan-targeted therapy	Hyaluronidase, degrades hyaluronan in the tumor microenvironment	[182]
Prostate cancer	PC-iPSCs	PTEN-deficient prostate cancer	BKM120	Enables investigation of PI3K inhibitor response	PI3K inhibitor, blocks PI3K signaling	[175]
Lung cancer	NSCLC-iPSCs	RET-rearranged non-small cell lung cancer	Selpercatinib	Reflects patient-specific response to RET inhibitors	RET inhibitor, blocks RET signaling	[183]
Colorectal cancer	CRC-iPSCs	KRAS wild-type colorectal cancer	Panitumumab	Represents patient-specific response to EGFR inhibitors	EGFR antibody, blocks EGFR signaling	[130, 172]
Liver cancer	Hep-iPSCs	Hepatocellular carcinoma with TP53 mutation	Tivantinib	Represents patient-specific response to MET inhibitors	MET inhibitor, blocks MET signaling	[179]
Pancreatic cancer	PDAC-iPSCs	Pancreatic ductal adenocarcinoma	Olaratumab	Enables investigation of platelet-derived growth factor receptor (PDGFR) inhibition	PDGFR antibody, inhibits PDGFR signaling	[184]
Breast cancer	BC-iPSCs	HER2-positive breast cancer	Lapatinib	Represents patient-specific response to HER2-targeted therapy	Dual HER2 and EGFR inhibitor, blocks HER2 and EGFR signaling	[185]
Prostate cancer	PC-iPSCs	PTEN-deleted prostate cancer	Abiraterone acetate	Enables investigation of androgen synthesis inhibitor response	Androgen synthesis inhibitor, inhibits androgen production	[186]
Lung cancer	NSCLC-iPSCs	EGFR T790M-positive non-small cell lung cancer	Osimertinib	Reflects patient-specific response to EGFR T790M inhibitors	EGFR T790M inhibitor, blocks EGFR T790M signaling	[187]
Colorectal cancer	CRC-iPSCs	MSI-high colorectal cancer	Pembrolizumab	Enables evaluation of immune checkpoint blockade	PD-1 antibody, enhances immune response	[146]
Liver cancer	Hep-iPSCs	Cholangiocarcinoma	Erdafitinib	Enables investigation of FGFR inhibitor response	FGFR inhibitor, blocks FGFR signaling	[188]
Pancreatic cancer	PDAC-iPSCs	Pancreatic ductal adenocarcinoma	Rucaparib	Enables investigation of PARP inhibitor response	PARP inhibitor, disrupts DNA repair mechanisms	[189]
Breast cancer	BC-iPSCs	Hormone receptor-positive, HER2-negative breast cancer	Palbociclib	Represents patient-specific response to CDK4/6 inhibitors	CDK4/6 inhibitor, inhibits cell cycle progression	[190]
Prostate cancer	PC-iPSCs	AR-V7-positive castration-resistant prostate cancer	Apalutamide	Represents patient-specific response to androgen receptor inhibitors	Androgen receptor inhibitor, blocks androgen receptor signaling	[191]
Lung cancer	NSCLC-iPSCs	ROS1-rearranged non-small cell lung cancer	Entrectinib	Reflects patient-specific response to ROS1 inhibitors	ROS1 inhibitor, blocks ROS1 signaling	[192]
Colorectal cancer	CRC-iPSCs	BRAF-mutated colorectal cancer	Encorafenib + Cetuximab	Represents patient-specific response to targeted combination therapy	BRAF inhibitor + EGFR antibody, dual pathway inhibition	[152]
Ovarian cancer	Ov-iPSCs	High-grade serous ovarian carcinoma	Bevacizumab	Enables investigation of anti-angiogenic therapy	VEGF antibody, inhibits angiogenesis	[193]
Liver cancer	Hep-iPSCs	Hepatocellular carcinoma with TP53 mutation	Lenvatinib + Pembrolizumab	Represents patient-specific response to combination targeted therapy and immune checkpoint blockade	Multi-kinase inhibitor + PD-1 antibody, dual pathway inhibition and enhances immune response	[194]
Prostate cancer	PC-iPSCs	Androgen-sensitive prostate cancer	Bicalutamide	Represents patient-specific response to androgen receptor antagonists	Androgen receptor antagonist, inhibits androgen receptor signaling	[143]
Colorectal cancer	CRC-iPSCs	Microsatellite stable colorectal cancer	Irinotecan	Represents patient-specific response to chemotherapy	Topoisomerase I inhibitor, disrupts DNA replication	[195]
Ovarian cancer	Ov-iPSCs	Mucinous ovarian carcinoma	Paclitaxel	Enables investigation of microtubule-targeting chemotherapy	Microtubule stabilizer, inhibits cell division	[149]
Liver cancer	Hep-iPSCs	Hepatocellular carcinoma with HBV infection	Entecavir	Represents patient-specific response to antiviral therapy	Nucleoside analog, inhibits viral replication	[196]
Pancreatic cancer	PDAC-iPSCs	Pancreatic ductal adenocarcinoma	Nivolumab	Enables evaluation of immune checkpoint blockade	PD-1 antibody, enhances immune response	[197]
Liver cancer	Hep-iPSCs	Hepatocellular carcinoma with MET amplification	Crizotinib	Represents patient-specific response to MET inhibitors	MET inhibitor, blocks MET signaling	[198]
Pancreatic cancer	PDAC-iPSCs	Pancreatic ductal adenocarcinoma	Onivyde	Enables investigation of liposomal chemotherapy	Liposomal formulation of irinotecan, improves drug delivery	[197]
Breast cancer	BC-iPSCs	HER2-positive breast cancer	Trastuzumab	Represents patient-specific response to HER2-targeted therapy	HER2 antibody, blocks HER2 signaling	[199]
Lung cancer	NSCLC-iPSCs	EGFR-mutated non-small cell lung cancer	Afatinib	Reflects patient-specific response to EGFR inhibitors	EGFR inhibitor, blocks EGFR signaling	[200]
Colorectal cancer	CRC-iPSCs	BRAF-mutated colorectal cancer	Vemurafenib	Represents patient-specific response to BRAF inhibitors	BRAF inhibitor, blocks BRAF signaling	[180]
Ovarian cancer	Ov-iPSCs	Serous ovarian carcinoma	Cisplatin	Enables investigation of platinum-based chemotherapy	Platinum-based chemotherapeutic agent, induces DNA damage	[201]
Pancreatic cancer	PDAC-iPSCs	Pancreatic ductal adenocarcinoma	Pemetrexed	Represents patient-specific response to chemotherapy	Antifolate chemotherapy agent, inhibits DNA synthesis	[202]
Lung cancer	NSCLC-iPSCs	KRAS-mutated non-small cell lung cancer	Tipifarnib	Represents patient-specific response to KRAS inhibitors	KRAS inhibitor, blocks KRAS signaling	[203, 204]
Colorectal cancer	CRC-iPSCs	MSI-high colorectal cancer	Nivolumab + Ipilimumab	Enables investigation of immune checkpoint blockade combination therapy	PD-1 antibody + CTLA-4 antibody, enhances immune response	[180]
Liver cancer	Hep-iPSCs	Hepatocellular carcinoma with FGFR fusion	Infigratinib	Represents patient-specific response to FGFR inhibitors	FGFR inhibitor, blocks FGFR signaling	[154, 205]
Pancreatic cancer	PDAC-iPSCs	Pancreatic ductal adenocarcinoma	Olaparib	Enables investigation of PARP inhibitor response	PARP inhibitor, disrupts DNA repair mechanisms	[206]
Breast cancer	BC-iPSCs	Luminal A breast cancer	Anastrozole	Represents patient-specific response to aromatase inhibitors	Aromatase inhibitor, blocks estrogen production	[129]
Prostate cancer	PC-iPSCs	TMPRSS2-ERG fusion-positive prostate cancer	Enzalutamide	Represents patient-specific response to androgen receptor inhibitors	Androgen receptor inhibitor, inhibits androgen receptor signaling	[207]
Lung cancer	NSCLC-iPSCs	MET exon 14 skipping non-small cell lung cancer	Capmatinib	Represents patient-specific response to MET inhibitors	MET inhibitor, blocks MET signaling	[200]
Ovarian cancer	Ov-iPSCs	Clear cell ovarian carcinoma	Temsirolimus	Enables investigation of mTOR inhibitor response	mTOR inhibitor, inhibits mTOR signaling	[208]
Pancreatic cancer	PDAC-iPSCs	Pancreatic ductal adenocarcinoma	Trametinib	Represents patient-specific response to MEK inhibitors	MEK inhibitor, inhibits MAPK signaling pathway	[209]
Breast cancer	BC-iPSCs	HER2-positive breast cancer	Lapatinib	Represents patient-specific response to dual HER2/EGFR inhibitors	Dual HER2/EGFR inhibitor, blocks HER2 and EGFR signaling	[210]
Prostate cancer	PC-iPSCs	Neuroendocrine prostate cancer	Cabazitaxel	Enables investigation of chemotherapy for neuroendocrine tumors	Microtubule-targeting chemotherapy agent, inhibits cell division	[211]
Colorectal cancer	CRC-iPSCs	Cetuximab-resistant colorectal cancer	Panitumumab	Represents patient-specific response to EGFR inhibitors	EGFR antibody, blocks EGFR signaling	[212]
Liver cancer	Hep-iPSCs	Hepatocellular carcinoma with TP53 mutation	Pembrolizumab	Enables evaluation of immune checkpoint blockade	PD-1 antibody, enhances immune response	[213]

### Personalized cancer treatment using iPSCs

The ability to generate patient-specific iPSCs opens up new possibilities for personalized cancer treatment [2]. Traditional cancer treatments, such as chemotherapy and radiation therapy, often lack specificity and can have significant side effects. iPSCs offer a unique opportunity to develop personalized therapies that target cancer cells while sparing healthy tissues [5]. By reprogramming somatic cells from patients into iPSCs, researchers can differentiate these iPSCs into various cell types, including immune cells [6]. iPSC-derived immune cells can be genetically modified to enhance their tumor-targeting capabilities, such as expressing chimeric antigen receptors (CARs) or T-cell receptors (TCRs) specific to cancer antigens [8]. These modified immune cells, also known as iPSC-derived CAR-T or TCR-T cells, can be expanded in large quantities and reinfused into the patient [2]. As these cells are derived from the patient’s own cells, the risk of immune rejection is minimized. Personalized cancer immunotherapies using iPSC-derived immune cells have shown promising results in preclinical studies [8]. They hold the potential to improve the efficacy and specificity of cancer treatment while reducing off-target effects. However, challenges such as ensuring the safety and effectiveness of iPSC-derived immune cells, optimizing their differentiation protocols, and overcoming immune evasion mechanisms employed by cancer cells need to be addressed before their widespread clinical application [9]. Table 4 provides an overview of the potential of personalized cancer treatment through the use of iPSCs.

**Table 4:** An overview of personalized cancer treatment utilizing iPSCs

Cancer Type	Specific iPSC Lines	iPSC Line Generated	Differentiation Potential Details	Characteristics (e.g., expression of tumor-specific markers)	References
Breast Cancer	iPSC-BRCA1	iPSCs reprogrammed from breast cancer patient with BRCA1 mutation	Can differentiate into epithelial and mesenchymal lineages	Expresses HER2 and estrogen receptor (ER)	[109, 214, 215]
Lung Cancer	iPSC-LUNG1	iPSCs derived from lung cancer patient	Differentiates into lung epithelial cells and fibroblasts	Expresses EGFR and KRAS mutations	[216]
Colon Cancer	iPSC-COLON2	Colon cancer patient-derived iPSCs	Shows potential to differentiate into colonic epithelial cells and smooth muscle cells	Overexpression of CD44 and β-catenin	[14, 217]
Prostate Cancer	iPSC-PROST1	iPSCs reprogrammed from prostate cancer patient	Can differentiate into prostate epithelial cells and stromal cells	Expresses androgen receptor (AR) and prostate-specific antigen (PSA)	[218–221]
Melanoma	iPSC-MELA2	Melanoma patient-derived iPSCs	Differentiates into melanocytes and neural crest cells	Expresses BRAF and MITF mutations	[222–224]
Leukemia	iPSC-LEUK1	iPSCs generated from leukemia patient	Differentiates into hematopoietic stem cells and lymphoid cells	Shows abnormal expression of leukemic markers (e.g., CD34, CD38, CD123)	[225–228]
Brain Tumor	iPSC-BRAIN3	iPSCs derived from brain tumor patient	Differentiates into neural stem cells and glial cells	Expresses EGFRvIII mutation and Nestin	[125, 229–231]
Ovarian Cancer	iPSC-OVARY1	iPSCs reprogrammed from ovarian cancer patient	Can differentiate into ovarian surface epithelial cells and granulosa cells	Overexpression of CA125 and BRCA2 mutation	[232–234]
Pancreatic Cancer	iPSC-PANCREAS2	Pancreatic cancer patient-derived iPSCs	Differentiates into pancreatic ductal cells and endocrine cells	Expresses KRAS and TP53 mutations	[235–238]
Sarcoma	iPSC-SARCOMA1	iPSCs generated from sarcoma patient	Can differentiate into mesenchymal stem cells and osteogenic cells	Overexpression of CD99 and EWSR1-FLI1 fusion gene	[239, 240]
Gastric Cancer	iPSC-GASTRIC3	iPSCs reprogrammed from gastric cancer patient	Differentiates into gastric epithelial cells and smooth muscle cells	Expresses HER2 and p53 mutations	[241–244]
Lymphoma	iPSC-LYMPHO2	iPSCs derived from lymphoma patient	Can differentiate into lymphoid cells and B cells	Abnormal expression of CD20 and BCL2	[245, 246]
Liver Cancer	iPSC-LIVER2	iPSCs reprogrammed from liver cancer patient	Can differentiate into hepatocytes and bile duct cells	Expresses AFP and HNF4A	[174, 247–249]
Bladder Cancer	iPSC-BLADDER1	Bladder cancer patient-derived iPSCs	Differentiates into urothelial cells and smooth muscle cells	Overexpression of CD44 and FGFR3 mutation	[250–252]
Esophageal Cancer	iPSC-ESOPHAGEAL3	iPSCs derived from esophageal cancer patient	Differentiates into esophageal epithelial cells and fibroblasts	Expresses HER2 and TP53 mutations	[253–255]
Bone Cancer	iPSC-BONE2	iPSCs reprogrammed from bone cancer patient	Can differentiate into osteoblasts and chondrocytes	Overexpression of CD99 and TP53 mutations	[256, 257]
Kidney Cancer	iPSC-KIDNEY1	Kidney cancer patient-derived iPSCs	Differentiates into renal epithelial cells and mesenchymal cells	Expresses VHL and MET mutations	[258–263]
Thyroid Cancer	iPSC-THYROID2	iPSCs reprogrammed from thyroid cancer patient	Can differentiate into thyroid follicular cells and C-cells	Overexpression of RET and BRAF mutations	[264, 265]
Prostate Cancer	iPSC-PROST2	iPSCs derived from prostate cancer patient	Can differentiate into prostate epithelial cells and neuroendocrine cells	Expresses AR and PTEN mutations	[52, 266–268]
Leukemia	iPSC-LEUK2	Leukemia patient-derived iPSCs	Differentiates into hematopoietic stem cells and myeloid cells	Shows abnormal expression of leukemic markers (e.g., CD33, CD123, MPO)	[118, 269–271]
Bone Marrow Cancer	iPSC-BONEMAR1	iPSCs reprogrammed from bone marrow cancer patient	Can differentiate into hematopoietic stem cells and plasma cells	Expresses abnormal immunoglobulin rearrangements	[272, 273]
Skin Cancer	iPSC-SKIN3	iPSCs derived from skin cancer patient	Differentiates into keratinocytes and melanocytes	Overexpression of BRAF and p16INK4a mutations	[274–276]
Head and Neck Cancer	iPSC-HEADNECK2	Head and neck cancer patient-derived iPSCs	Differentiates into squamous epithelial cells and fibroblasts	Expresses EGFR and TP53 mutations	[277–279]
Soft Tissue Sarcoma	iPSC-SOFTTIS2	iPSCs reprogrammed from soft tissue sarcoma patient	Can differentiate into mesenchymal stem cells and adipocytes	Overexpression of MDM2 and CDK4	[241, 280–282]
Osteosarcoma	iPSC-OSTEO1	Osteosarcoma patient-derived iPSCs	Differentiates into osteoblasts and osteocytes	Expresses TP53 and RB1 mutations	[240, 283, 284]
Brain Cancer	iPSC-BRAIN4	iPSCs reprogrammed from brain cancer patient	Can differentiate into neuronal cells and astrocytes	Overexpression of EGFRvIII and PTEN mutations	[285–289]
Cervical Cancer	iPSC-CERVICAL2	iPSCs derived from cervical cancer patient	Differentiates into cervical epithelial cells and mesenchymal cells	Expresses HPV integration and p53 mutations	[290, 291]
Gallbladder Cancer	iPSC-GALLBLAD1	Gallbladder cancer patient-derived iPSCs	Can differentiate into gallbladder epithelial cells and smooth muscle cells	Overexpression of HER2 and TP53 mutations	[292, 293]
Testicular Cancer	iPSC-TESTIS2	iPSCs reprogrammed from testicular cancer patient	Differentiates into testicular germ cells and Sertoli cells	Expresses OCT4 and NANOG	[294, 295]
Uterine Cancer	iPSC-UTERINE1	Uterine cancer patient-derived iPSCs	Can differentiate into uterine epithelial cells and stromal cells	Overexpression of PTEN and CTNNB1 mutations	[296, 297]
Multiple Myeloma	iPSC-MYEL1	iPSCs reprogrammed from multiple myeloma patient	Can differentiate into plasma cells and bone marrow stromal cells	Overexpression of IgH translocations and MAF mutations	[273, 298]
Gallbladder Cancer	iPSC-GALLBLAD2	Gallbladder cancer patient-derived iPSCs	Differentiates into gallbladder epithelial cells and smooth muscle cells	Expresses HER2 and TP53 mutations	[299, 300]
Bladder Cancer	iPSC-BLADDER2	iPSCs reprogrammed from bladder cancer patient	Can differentiate into urothelial cells and fibroblasts	Overexpression of FGFR3 and p53 mutations	[251, 301]
Glioblastoma	iPSC-GLIO1	Glioblastoma patient-derived iPSCs	Differentiates into neural stem cells and astrocytes	Expresses EGFRvIII and PTEN mutations	[302, 303]
Ovarian Cancer	iPSC-OVARY2	iPSCs reprogrammed from ovarian cancer patient	Can differentiate into ovarian surface epithelial cells and granulosa cells	Overexpression of CA125 and BRCA1 mutations	[232, 233, 304]
Liver Cancer	iPSC-LIVER3	Liver cancer patient-derived iPSCs	Differentiates into hepatocytes and bile duct cells	Expresses AFP and TP53 mutations	[305–307]
Ewing Sarcoma	iPSC-EWING1	iPSCs reprogrammed from Ewing sarcoma patient	Can differentiate into mesenchymal stem cells and osteoblasts	Overexpression of EWSR1-FLI1 fusion gene	[239, 308, 309]
Retinoblastoma	iPSC-RETINO1	Retinoblastoma patient-derived iPSCs	Differentiates into retinal progenitor cells and photoreceptor cells	Expresses RB1 mutation and abnormal retinal development markers	[256, 310]
Pancreatic Cancer	iPSC-PANCREAS3	iPSCs reprogrammed from pancreatic cancer patient	Can differentiate into pancreatic ductal cells and endocrine cells	Overexpression of KRAS and TP53 mutations	[236, 238, 311]
Nasopharyngeal Cancer	iPSC-NASOPHAR1	Nasopharyngeal cancer patient-derived iPSCs	Differentiates into nasopharyngeal epithelial cells and fibroblasts	Expresses EBV infection and TP53 mutations	[312–314]
Renal Cell Carcinoma	iPSC-RENAL1	iPSCs reprogrammed from renal cell carcinoma patient	Can differentiate into renal tubular cells and mesenchymal cells	Overexpression of VHL and MET mutations	[301, 315, 316]
Ovarian Germ Cell Tumor	iPSC-OVARYG1	Ovarian germ cell tumor patient-derived iPSCs	Differentiates into germ cell-like cells and ovarian stromal cells	Expresses OCT4 and SOX17	[65, 317, 318]
Adrenocortical Carcinoma	iPSC-ADRENAL1	iPSCs reprogrammed from adrenocortical carcinoma patient	Can differentiate into adrenocortical cells and steroidogenic cells	Overexpression of TP53 and CTNNB1 mutations	[241, 319]
Cholangiocarcinoma	iPSC-CHOLANGIO1	Cholangiocarcinoma patient-derived iPSCs	Differentiates into cholangiocytes and hepatocytes	Expresses FGFR2 fusion gene and abnormal bile duct markers	[136, 320]
Neuroblastoma	iPSC-NEUROBLAST1	iPSCs reprogrammed from neuroblastoma patient	Can differentiate into neural crest cells and sympathetic neurons	Overexpression of MYCN and ALK mutations	[321, 322]
Thymoma	iPSC-THYMOMA1	Thymoma patient-derived iPSCs	Differentiates into thymic epithelial cells and T lymphocytes	Expresses TTF-1 and MAGE markers	[323, 324]
Merkel Cell Carcinoma	iPSC-MERKEL1	iPSCs reprogrammed from Merkel cell carcinoma patient	Can differentiate into Merkel cells and keratinocytes	Overexpression of Merkel cell polyomavirus and p63	[325]
Choriocarcinoma	iPSC-CHORIO1	Choriocarcinoma patient-derived iPSCs	Differentiates into trophoblast cells and placental cells	Expresses abnormal hCG production and p57 marker	[326–328]
Adrenal Cortex Carcinoma	iPSC-ADRENAL2	iPSCs reprogrammed from adrenal cortex carcinoma patient	Can differentiate into adrenocortical cells and steroidogenic cells	Overexpression of CTNNB1 and TP53 mutations	[329, 330]
Uveal Melanoma	iPSC-UVEAL1	Uveal melanoma patient-derived iPSCs	Differentiates into melanocytes and neural crest cells	Expresses GNAQ and SF3B1 mutations	[224]
Osteoblastoma	iPSC-OSTEOBL1	iPSCs reprogrammed from osteoblastoma patient	Can differentiate into osteoblasts and osteocytes	Overexpression of HMGA2 and CDK4	[331–333]
Ampullary Carcinoma	iPSC-AMPULLARY1	Ampullary carcinoma patient-derived iPSCs	Differentiates into ampullary epithelial cells and smooth muscle cells	Expresses KRAS and TP53 mutations	[292, 334]
Pleural Mesothelioma	iPSC-MESOTHELI1	iPSCs reprogrammed from pleural mesothelioma patient	Can differentiate into mesothelial cells and fibroblasts	Overexpression of BAP1 and CDKN2A mutations	[335, 336]
Neuroendocrine Tumor	iPSC-NEUROENDO1	Neuroendocrine tumor patient-derived iPSCs	Differentiates into neuroendocrine cells and pancreatic islet cells	Expresses chromogranin A and MEN1 mutation	[337]
Small Cell Lung Cancer	iPSC-LUNGSM1	iPSCs reprogrammed from small cell lung cancer patient	Can differentiate into lung epithelial cells and neuroendocrine cells	Overexpression of MYC and TP53 mutations	[338, 339]
Chondrosarcoma	iPSC-CHONDRO1	Chondrosarcoma patient-derived iPSCs	Differentiates into chondrocytes and mesenchymal cells	Expresses IDH1 and TP53 mutations	[340–342]
Ampulla of Vater Cancer	iPSC-AMPULLA2	iPSCs reprogrammed from ampulla of Vater cancer patient	Can differentiate into ampullary epithelial cells and smooth muscle cells	Overexpression of KRAS and TP53 mutations	[343, 344]
Salivary Gland Cancer	iPSC-SALIVARY1	Salivary gland cancer patient-derived iPSCs	Differentiates into salivary gland epithelial cells and myoepithelial cells	Expresses MYB-NFIB fusion gene and abnormal salivary gland markers	[250, 345, 346]
Primary Central Nervous System Lymphoma	iPSC-PCNSL1	iPSCs reprogrammed from primary central nervous system lymphoma patient	Can differentiate into lymphoid cells and B cells	Overexpression of MYD88 and CD79B mutations	[125, 347]
Gastrointestinal Stromal Tumor	iPSC-GIST1	Gastrointestinal stromal tumor patient-derived iPSCs	Differentiates into gastrointestinal stromal cells and smooth muscle cells	Expresses KIT and PDGFRA mutations	[341]
Choriocarcinoma	iPSC-CHORIO2	Choriocarcinoma patient-derived iPSCs	Can differentiate into trophoblast cells and placental cells	Overexpression of NLRP7 and p57 markers	[348, 349]
Waldenström Macroglobulinemia	iPSC-WALDEN1	iPSCs reprogrammed from Waldenström macroglobulinemia patient	Differentiates into plasma cells and B lymphocytes	Expresses MYD88 and CXCR4 mutations	[341]
Liposarcoma	iPSC-LIPO1	Liposarcoma patient-derived iPSCs	Can differentiate into adipocytes and fibroblasts	Overexpression of MDM2 and CDK4	[341, 350, 351]
Vulvar Cancer	iPSC-VULVAR1	Vulvar cancer patient-derived iPSCs	Differentiates into vulvar epithelial cells and fibroblasts	Expresses p16INK4a and HPV infection	[352, 353]
Chronic Lymphocytic Leukemia	iPSC-CLL1	iPSCs reprogrammed from chronic lymphocytic leukemia patient	Can differentiate into lymphoid cells and B cells	Overexpression of TP53 and ATM mutations	[354–356]
Fibrolamellar Carcinoma	iPSC-FIBRO1	Fibrolamellar carcinoma patient-derived iPSCs	Differentiates into hepatocytes and bile duct cells	Expresses DNAJB1-PRKACA fusion gene	[305, 357]
Synovial Sarcoma	iPSC-SYNOV1	Synovial sarcoma patient-derived iPSCs	Can differentiate into mesenchymal cells and synoviocytes	Overexpression of SS18-SSX fusion gene	[358, 359]
Esophageal Cancer	iPSC-ESOPHAGEAL2	iPSCs reprogrammed from esophageal cancer patient	Differentiates into esophageal epithelial cells and smooth muscle cells	Expresses TP53 and EGFR mutations	[254, 360]
Malignant Melanoma	iPSC-MELANOMA2	Malignant melanoma patient-derived iPSCs	Can differentiate into melanocytes and neural crest cells	Overexpression of BRAF and NRAS mutations	[274, 361]
Hepatoblastoma	iPSC-HEPATOBL1	iPSCs reprogrammed from hepatoblastoma patient	Differentiates into hepatocyte-like cells and bile duct cells	Expresses abnormal AFP and Wnt signaling	[362, 363]
Pleomorphic Sarcoma	iPSC-PLEOMORPH1	Pleomorphic sarcoma patient-derived iPSCs	Can differentiate into mesenchymal cells and fibroblasts	Overexpression of TP53 and RB1 mutations	[254, 360]
Myelodysplastic Syndrome	iPSC-MDS1	iPSCs reprogrammed from myelodysplastic syndrome patient	Differentiates into hematopoietic stem cells and myeloid cells	Expresses abnormal cytogenetics and dysplastic markers	[364–366]
Uterine Sarcoma	iPSC-UTERINE1	iPSCs reprogrammed from uterine sarcoma patient	Can differentiate into uterine smooth muscle cells and fibroblasts	Overexpression of MED12 and TP53 mutations	[367, 368]
Thymic Carcinoma	iPSC-THYMIC1	Thymic carcinoma patient-derived iPSCs	Differentiates into thymic epithelial cells and T lymphocytes	Expresses FOXN1 and p63 markers	[369–371]
Rhabdomyosarcoma	iPSC-RHABDO1	iPSCs reprogrammed from rhabdomyosarcoma patient	Can differentiate into myoblasts and skeletal muscle cells	Overexpression of PAX3-FOXO1 fusion gene	[372–375]
Gastric Cancer	iPSC-GASTRIC2	iPSCs reprogrammed from gastric cancer patient	Differentiates into gastric epithelial cells and mesenchymal cells	Expresses HER2 and CDH1 mutations	[242, 243, 376]
Angiosarcoma	iPSC-ANGIO1	Angiosarcoma patient-derived iPSCs	Can differentiate into endothelial cells and pericytes	Overexpression of MYC and TP53 mutations	[377–379]
Thymoma	iPSC-THYMOMA2	Thymoma patient-derived iPSCs	Differentiates into thymic epithelial cells and T lymphocytes	Expresses CD5 and CD117 markers	[255, 322, 380]
Osteosarcoma	iPSC-OSTEO1	Osteosarcoma patient-derived iPSCs	Can differentiate into osteoblasts and bone cells	Overexpression of TP53 and RB1 mutations	[240, 283, 381]
Glioblastoma	iPSC-GLIOBLAST1	iPSCs reprogrammed from glioblastoma patient	Differentiates into neural stem cells and glial cells	Expresses EGFR amplification and IDH1 mutation	[382–385]
Uterine Leiomyosarcoma	iPSC-LEIOMYO1	Uterine leiomyosarcoma patient-derived iPSCs	Can differentiate into smooth muscle cells and fibroblasts	Overexpression of HMGA2 and TP53 mutations	[386]
Ovarian Cancer	iPSC-OVARIAN2	iPSCs reprogrammed from ovarian cancer patient	Differentiates into ovarian epithelial cells and stromal cells	Expresses BRCA1/2 mutations and abnormal hormone receptor status	[232, 233, 387]
Chordoma	iPSC-CHORDO1	Chordoma patient-derived iPSCs	Can differentiate into notochordal cells and mesenchymal cells	Overexpression of T gene and brachyury	[388, 389]
Hepatocellular Carcinoma	iPSC-HEPATOC1	iPSCs reprogrammed from hepatocellular carcinoma patient	Differentiates into hepatocytes and bile duct cells	Expresses abnormal AFP and abnormal liver-specific markers	[320, 390, 391]
Bladder Cancer	iPSC-BLADDER1	iPSCs reprogrammed from bladder cancer patient	Can differentiate into urothelial cells and smooth muscle cells	Overexpression of FGFR3 and TP53 mutations	[216, 251, 301, 392]
Medulloblastoma	iPSC-MEDULLO1	Medulloblastoma patient-derived iPSCs	Differentiates into neural stem cells and cerebellar cells	Expresses PTCH1 and MYC amplification	[393, 394]
Soft Tissue Sarcoma	iPSC-SOFTTISSUE1	Soft tissue sarcoma patient-derived iPSCs	Can differentiate into mesenchymal cells and fibroblasts	Overexpression of MDM2 and CDK4	[341, 395, 396]
Renal Cell Carcinoma	iPSC-RENAL1	iPSCs reprogrammed from renal cell carcinoma patient	Differentiates into renal epithelial cells and mesenchymal cells	Expresses VHL and MET mutations	[241, 397]
Ovarian Germ Cell Tumor	iPSC-GERMCELL1	Ovarian germ cell tumor patient-derived iPSCs	Can differentiate into germ cells and ovarian follicular cells	Overexpression of OCT3/4 and c-KIT mutations	[318, 398]
Basal Cell Carcinoma	iPSC-BASAL1	iPSCs reprogrammed from basal cell carcinoma patient	Differentiates into basal keratinocytes and hair follicle cells	Expresses PTCH1 and TP53 mutations	[399, 400]
Pancreatic Cancer	iPSC-PANCREATIC2	iPSCs reprogrammed from pancreatic cancer patient	Can differentiate into pancreatic ductal cells and acinar cells	Overexpression of KRAS and TP53 mutations	[236, 401, 402]
Ovarian Clear Cell Carcinoma	iPSC-CLEARCELL1	Ovarian clear cell carcinoma patient-derived iPSCs	Differentiates into ovarian clear epithelial cells and stromal cells	Expresses ARID1A and PIK3CA mutations	[233, 403]
Prostate Cancer	iPSC-PROSTATE2	iPSCs reprogrammed from prostate cancer patient	Can differentiate into prostate epithelial cells and smooth muscle cells	Overexpression of PTEN and TMPRSS2-ERG fusion gene	[267, 404–406]
Ewing Sarcoma	iPSC-EWING1	Ewing sarcoma patient-derived iPSCs	Differentiates into mesenchymal stem cells and bone cells	Expresses EWS-FLI1 fusion gene	[239, 407]
Head and Neck Squamous Cell Carcinoma	iPSC-HEADNECK1	iPSCs reprogrammed from head and neck squamous cell carcinoma patient	Can differentiate into squamous epithelial cells and fibroblasts	Overexpression of TP53 and HPV infection	[278, 408, 409]
Primary Brain Tumor	iPSC-BRAINTUMOR1	Primary brain tumor patient-derived iPSCs	Differentiates into neural stem cells and glial cells	Expresses IDH1 and EGFR amplification	[125, 230]

### iPSCs for developing immunotherapies for cancer

Immunotherapies have emerged as a breakthrough approach in cancer treatment, harnessing the power of the immune system to selectively target and eliminate cancer cells. iPSCs offer unique advantages for developing immunotherapies by providing an unlimited source of immune cells for manipulation and expansion [8]. One promising strategy is the generation of iPSC-derived dendritic cells (DCs). DCs play a crucial role in initiating and regulating immune responses. iPSC-derived DCs can be engineered to express specific tumor antigens or antigen-presenting molecules, enhancing their ability to activate immune cells against cancer cells [10]. These iPSC-derived DCs can be used as vaccines to stimulate an anti-tumor immune response in patients. Additionally, iPSCs can be differentiated into natural killer (NK) cells, a type of immune cell known for their ability to recognize and kill cancer cells. iPSC-derived NK cells can be genetically engineered to enhance their tumor-targeting capabilities and to improve their persistence and cytotoxicity [11]. These modified iPSC-derived NK cells can then be used as a potent immunotherapy for cancer treatment [12]. Furthermore, iPSCs can be utilized to generate tumor-specific T cells. Tumor-infiltrating lymphocytes (TILs) obtained from cancer patients have shown promising results in immunotherapy. However, their limited supply and functional exhaustion pose challenges for clinical applications [13]. iPSCs provide a renewable source for generating TILs in large quantities. iPSC-derived T cells can be genetically modified to express tumor-specific TCRs or CARs, enabling them to recognize and eliminate cancer cells [14]. These iPSC-derived T cells can be expanded and infused back into the patient to mount an effective anti-tumor immune response [15]. The use of iPSCs for developing immunotherapies offers the potential for personalized treatments and improved clinical outcomes [15]. By combining the advantages of iPSC technology with the specificity and potency of immune cells, researchers are paving the way for more targeted and effective cancer therapies. Table 5 highlights the utilization of iPSCs in the advancement of immunotherapies for cancer.

**Table 5:** iPSCs for developing immunotherapies for cancer

Cancer Type	In vitro Model of Cancer Cells	Immune Response Observed	Comparison with Traditional Cancer Cell Lines	References
Breast Cancer	3D organoid culture	Enhanced tumor antigen presentation and T cell activation	Better recapitulation of tumor microenvironment and cellular heterogeneity	[410–412]
Lung Cancer	Co-culture with immune cells	Increased production of cytotoxic T lymphocytes (CTLs) and cytokines	Improved immune checkpoint inhibitor screening and drug development	[413, 414]
Colorectal Cancer	Patient-derived tumor spheroids	Enhanced infiltration and activation of NK cells	Better representation of tumor-stromal interactions and drug sensitivity	[415–417]
Melanoma	2D and 3D melanoma models	Induction of tumor-specific CD8 + T cell response	Accurate modeling of immune evasion and resistance mechanisms	[418–420]
Prostate Cancer	Tumor-stroma co-culture system	Augmented tumor-associated macrophage polarization and cytokine secretion	Improved understanding of tumor-immune cell crosstalk and therapeutic targeting	[421, 422]
Leukemia	Hematopoietic differentiation	Generation of functional NK cells for adoptive cell therapy	Enhanced scalability and standardized production of immune effector cells	[423–425]
Pancreatic Cancer	Pancreatic tumor organoids	Activation of tumor-infiltrating lymphocytes (TILs) and increased cytotoxicity	Recapitulation of tumor architecture and drug screening using patient-specific iPSCs	[426, 427]
Ovarian Cancer	3D tumor spheroids	Enhanced infiltration and activation of DCs	Improved study of antigen presentation and personalized immunotherapy	[428–430]
Brain Cancer	Glioblastoma multiforme (GBM) organoids	Immune checkpoint blockade-induced tumor regression	More reliable testing of immune checkpoint inhibitors and personalized treatment strategies	[431–433]
Gastric Cancer	Co-culture with tumor-associated fibroblasts (TAFs)	Modulation of myeloid-derived suppressor cells (MDSCs) and improved anti-tumor immune response	Better understanding of tumor-stromal interactions and therapeutic resistance mechanisms	[434, 435]
Lymphoma	Hematopoietic differentiation	Generation of CAR T cells for targeted therapy	Scalable production of CAR T cells from patient-specific iPSCs	[436–438]
Bladder Cancer	Bladder cancer cell line co-culture	Induction of tumor-specific CD4 + T helper cell response	Accurate modeling of immune evasion and immunosuppressive mechanisms	[439, 440]
Pancreatic Cancer	Patient-derived pancreatic tumor organoids	Activation of tumor-specific CD4 + and CD8 + T cells	Better representation of tumor heterogeneity and drug response	[441–445]
Sarcoma	3D scaffold-based tumor models	Increased infiltration and activation of TILs	Improved understanding of immune evasion mechanisms in sarcoma	[407, 446, 447]
Liver Cancer	Liver tumor organoids	Enhanced NK cell cytotoxicity against cancer cells	Better recapitulation of liver tumor microenvironment and drug screening	[448–452]
Head and Neck Cancer	Co-culture with oral epithelial cells	Induction of anti-tumor immune response through enhanced antigen presentation	Study of immune escape mechanisms and development of targeted therapies	[453]
Bone Cancer	Osteosarcoma tumor spheroids	Activation of osteoclasts and osteoblasts for bone remodeling and immune cell interaction	Improved understanding of bone metastasis and novel immunotherapeutic targets	[454–456]
Esophageal Cancer	3D esophageal organoids	Generation of tumor-specific CTLs for adoptive cell therapy	Accurate modeling of tumor-stromal interactions and drug response in esophageal cancer	[457, 458]
Prostate Cancer	Prostate tumor organoids	Activation of tumor-specific CD4 + T helper cells and CD8 + CTLs	Better representation of tumor heterogeneity and drug screening	[267, 459–462]
Brain Cancer	Glioblastoma stem cell culture	Enhanced infiltration of immune effector cells and increased cytokine production	Improved understanding of the tumor microenvironment and immunomodulation	[302, 463]
Cervical Cancer	Co-culture with cervical epithelial cells	Induction of tumor-specific CD8 + T cell response and cytotoxicity	Enhanced study of human papillomavirus (HPV)-related immune responses	[464]
Bone Marrow Cancer	Hematopoietic differentiation	Generation of functional NK cells and DCs for immunotherapy	Scalable production of immune effector cells with desired functionalities	[25, 465, 466]
Liver Cancer	3D liver tumor organoids	Enhanced activation of tumor-infiltrating lymphocytes (TILs) and immune checkpoint modulation	Improved mimicry of liver tumor microenvironment and drug response	[467–469]
Thyroid Cancer	Thyroid tumor spheroids	Induction of tumor-specific antibody production by B cells	Study of tumor-immune cell interactions and antibody-based therapies	[470–472]

## Mechanisms of iPSC-based cancer therapy

The iPSCs have shown tremendous potential in the field of cancer therapy. Their unique characteristics, such as pluripotency and self-renewal, enable the development of innovative approaches for the treatment of various types of cancer [473].

### iPSC-derived cancer cells for drug screening and personalized treatment

**Fig. 8:**
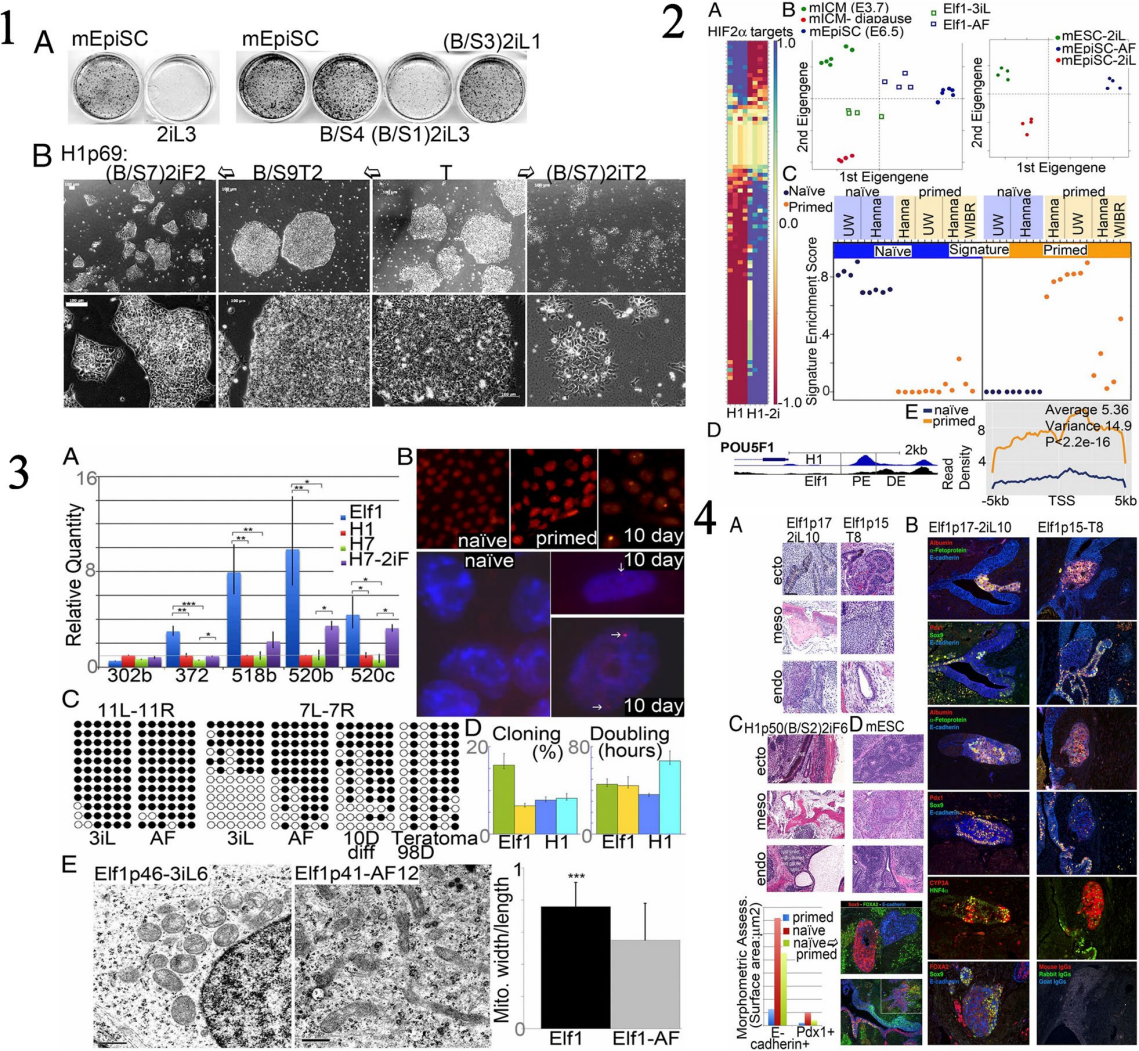
**1** The impact of 2i culture on pluripotent cells in mice and humans. In panel A, mouse pluripotent colonies were subjected to alkaline phosphatase staining. The two plates on the left show that the addition of 2i to mEpiSC colonies leads to differentiation and a loss of alkaline phosphatase positive cells. However, the four plates on the right demonstrate that when mEpiSCs are grown in butyrate plus SAHA before introducing 2i, pluripotent colonies thrive. In panel B, human embryonic stem cells (H1) were either directed back to 2i culture through butyrate exposure or pushed forward towards differentiation without prior exposure to B/S. This indicates that 2i culture must follow B/S exposure to prevent differentiation. The scale bars in the figure represent a length of 100 μM. **2** The genomic analysis conducted on naïve human embryonic stem cells (hESCs). In panel (A), a heat map displays the RNA expression levels of target genes regulated by HIF2α (EPAS1) in H1-2iF cells compared to the parent H1 cells. The comparison was performed using quadruplicate samples and the cells were cultured in TeSR2. Panel (B) presents a principal component analysis (PCA) plot comparing mouse whole genome Agilent array data. The left side of the plot shows embryo data from Hunter et al. (8), while the right side shows mouse embryonic stem cell (mESC) equivalents, including R1p22 (mESC-2iL, naïve), mEpiSC7p24AF (mEpiSC-AF, primed), and mEpiSC7p55(AF7,B/S1)2iL20 (mEpiSC-2iL, toggled to naïve). The expression data of naïve (3iL, green squares) and primed (AF, blue squares) Elf1 cells are also compared with in vivo mouse embryo data in the plot on the left. In panel (C), a comparison is shown between in-house Elf1 expression array data and data generated by Hanna et al. (5). The comparison includes naïve and primed cell lines, represented by dark blue dots and orange dots, respectively. The lines tested on the left side of the graph are grouped identically on the Elf1 primed side. Panel (D) displays DNase I hypersensitivity analysis of the enhancer regions for the POU5F1 gene in Elf1 and H1 cells. The lower black line represents Elf1, and the blue line above represents H1. The first exon of POU5F1 is shown above the H1 data, along with a 2-kb size bar indicating the proximal enhancer (PE) and distal enhancer (DE). In panel (E), a comparison of ChIP-seq H3K27me3 data is presented. The orange line represents primed hESCs (data from Gafni et al., 6), while the blue line represents naïve Elf1-2iL cells. The comparison focuses on the subset of genes from panel (C) that are associated with Gene Ontology “developmental genes” (*n* = 648). **3** The analysis of different stages of pluripotency in human embryonic stem cells (hESCs). Panel (A) shows the results of microRNA analysis related to pluripotency. Panel (B) demonstrates the labeling of XIST (X-inactive specific transcript) using a technique called fluorescence in situ hybridization (FISH). In the left image, Elf1-3iLs cells do not show a cloud-like XIST signal, whereas Elf1s primed cells exhibit two XIST signals. Furthermore, cells differentiated for 10 days display a single XIST signal (represented by a red dot) within the nucleus. When the nucleus is highlighted using DAPI staining and the field is magnified, XIST remains undetectable in naïve Elf1 cells (lower left). However, upon differentiation, the XIST signal becomes detectable on one or both X chromosomes (red dots, white arrows, lower right). Panel (C) presents the results of bisulfite sequencing of the XIST promoter using different primer sets. The figure shows that XIST remains methylated throughout the naïve and primed stages. However, using specific primers, methylation appears to decrease in naïve cells compared to primed cells. This decrease is also observed in in vitro-differentiated cells and in a teratoma at day 98. The circles represent CpG sites, where open circles indicate unmethylated and filled circles indicate methylated CpGs. Panel (D) consists of graphs representing the cloning efficiency (percentage) and doubling times (hours) of Elf1 naive, Elf1 primed, H1 naive, and H1 primed cells. Panel (E) displays electron microscopy images of mitochondria. The left panels highlight the difference in mitochondrial shape between Elf1-3iL and Elf1-AF cells. This difference is quantified in the graph on the right, where an increased ratio indicates a rounder population of mitochondria. The error bars represent the standard error of the mean (SEM). **4** The developmental capacity of teratomas derived from different types of cells. Panel A displays sections of teratomas labeled with H&E staining. Specifically, it shows sections from Elf1p17-2iL10 teratoma (naïve; 42 days) and Elf1p15T8 teratoma (primed, 67 days). Panel B focuses on endoderm-specific labeling of the Elf1 teratomas shown in Panel A. The upper two panels of both tumors represent sequential sections. The upper panel highlights liver development using red (albumin), green (α-fetoprotein), and blue (E-cadherin) labeling. The second set of panels highlights pancreatic development using red (PDX1), green (SOX9), and blue (E-cadherin) labeling. The next three panels (descending) in both tumors are different sequential sections. The first set represents liver development, the second set represents pancreatic development, and the third set represents liver development using alternative markers (labeled as above and red, CYP3A and green, HNF4A). The lower panel of Elf1p17-2iL10 (naïve) is included to emphasize the level of organization of endodermal development within these tumors, labeled with red (FOXA2), green (SOX9), and blue (E-cadherin). The bottom right panel (Elf1p15T8) serves as a negative control. Panel C displays H&E sections of an H1 naïve teratoma (44 days). The graph below the H&E sections quantifies the areas stained for E-cadherin (epithelial cells) or PDX1 (pancreatic progenitors) in primed (H1p44-AF9), naïve [H1p49(B/S3)2iF10], and naïve reverted to primed [H1p49(B/S3, 2iF4)AF5] H1 generated teratomas. The graph indicates that both the overall epithelial developmental potential and the pancreatic subset are enhanced in the naïve state compared to the primed state. Panel D shows the top three panels with H&E sections of an mESC teratoma (naïve, 13 days). The lower panels display immunofluorescent labeling of sections from this mESC teratoma. The scale bars in panels A and D define the scale for all H&E-stained sections and are set at 100 μM. Reprinted from [477] with permission from the PNAS

One of the major challenges in cancer treatment is identifying effective drugs that specifically target cancer cells while sparing healthy cell [474]. iPSCs offer a valuable tool for addressing this challenge by providing a platform for generating patient-specific cancer cells [475]. By reprogramming somatic cells from cancer patients into iPSCs, it is possible to differentiate them back into cancer cells representing the patient’s specific tumor type [15]. These iPSC-derived cancer cells can be used for drug screening and testing the efficacy of various anticancer drugs [105]. Researchers can expose these cells to different compounds and observe their response, allowing for the identification of personalized treatment options. This approach has the potential to improve the success rate of cancer treatment by tailoring therapies to individual patients based on the characteristics of their specific cancer cells [476]. In a recent investigation carried out by Ware et al. (2014), the primary focus was on obtaining nontransgenic hESCs in a naïve state and examining their properties and potential for development [477]. This study builds upon prior research that had demonstrated the advantages of achieving a naïve pluripotent state in mice while highlighting the challenges in replicating the same state in human cells. The researchers employed two strategies for obtaining naïve hESCs. Firstly, they successfully converted existing primed hESC lines into the naïve state by exposing them to histone deacetylase inhibitors, followed by cultivation with MEK/ERK and GSK3 inhibitors (2i) along with FGF2. Secondly, they directly derived naïve hESCs from human embryos using 2i and FGF2. The resultant naïve hESCs exhibited traits consistent with the naïve state, including growth patterns, gene expression, X-inactivation profile, mitochondrial morphology, microRNA profile, and developmental potential in teratomas. Importantly, this research also underscores the role of iPSCs in the context of naïve hESCs. iPSCs are somatic cells reprogrammed to a pluripotent state akin to embryonic stem cells. In Fig. 8, the researchers illustrate the influence of 2i culture on both mouse and human pluripotent cells, emphasizing the adaptability of these culture conditions to various pluripotent cell types, including iPSCs. Furthermore, Fig. 8 presents diverse genomic analyses conducted on naïve hESCs, shedding light on the molecular attributes of these cells and their comparison to other pluripotent states, including iPSCs. Additionally, Fig. 8 offers a comprehensive analysis of the stages of pluripotency in hESCs using various techniques, providing insights into the distinct characteristics of naïve hESCs and their relationship with iPSCs. Lastly, Fig. 8 illustrates the developmental potential of teratomas derived from different cell states, encompassing naïve and primed hESCs. This underscores the importance of comprehending and characterizing pluripotent states, including naïve and iPSCs, due to their significant implications for research in regenerative medicine and developmental biology.

### iPSCs for developing cancer vaccines and immunotherapies

Immunotherapy has emerged as a promising approach for cancer treatment, harnessing the body’s immune system to target and eliminate cancer cells. iPSCs offer a novel avenue for developing cancer vaccines and immunotherapies [478]. Researchers can reprogram iPSCs to express specific tumor antigens, which are proteins found on the surface of cancer cells that can trigger an immune response [479]. These iPSCs can be differentiated into dendritic cells or other antigen-presenting cells, which are crucial for activating the immune system [480]. The iPSC-derived antigen-presenting cells can then be used to stimulate the patient’s immune system, priming it to recognize and attack cancer cells expressing the specific tumor antigens. This approach holds promise for the development of personalized immunotherapies that target the unique characteristics of each patient’s cancer cells [481]. Table 6 highlights the significant role of iPSCs in the field of cancer vaccines and immunotherapies.

**Table 6:** iPSCs for developing cancer vaccines and immunotherapies

iPSC Line Used	Cancer Type	Immune Response Observed	Comparison with Traditional Cancer Cell Lines	Signal Pathways	References
iPSC-derived dendritic cells	Melanoma	Activation of CD8 + T cells	More efficient antigen presentation compared to traditional dendritic cells	Not applicable	[482]
iPSC-derived NK cells	Various solid tumors	Enhanced cytotoxicity towards tumor cells	More effective than peripheral blood-derived NK cells	NKG2D, DNAM-1, and NKp30 pathways	[131]
iPSC-derived CAR-T cells	B-cell acute lymphoblastic leukemia (B-ALL)	Potent killing of B-ALL cells	Higher efficacy and specificity compared to CAR-T cells derived from peripheral blood	CD19 and CD22 pathways	[483]
iPSC-derived TCR-T cells	Melanoma	Antigen-specific cytotoxicity towards melanoma cells	Enhanced specificity and reduced toxicity compared to TCR-T cells derived from peripheral blood	TCR signaling pathway	[484]
iPSC-derived tumor cells	Various solid tumors	Induction of tumor-specific immune responses	More closely resemble primary tumors compared to traditional cancer cell lines	Not applicable	[131]
iPSC-derived mesenchymal stem cells	Various solid tumors	Modulation of immune response towards tumor cells	More effective than mesenchymal stem cells derived from bone marrow	TGF-β and PGE2 pathways	[131]
iPSC-derived tumor-associated macrophages	Breast cancer	Promotion of tumor cell phagocytosis and activation of antitumor immune response	Enhanced phagocytic capacity and polarization compared to traditional macrophage cell lines	Toll-like receptor (TLR) signaling pathway	[214]
iPSC-derived cancer stem cells	Colorectal cancer	Generation of tumor-specific cytotoxic T lymphocytes (CTLs)	More efficient generation of CTLs targeting cancer stem cell antigens compared to traditional cancer stem cell lines	Wnt/β-catenin signaling pathway	[130]
iPSC-derived antigen-presenting cells (APCs)	Lung cancer	Activation of tumor-specific CD4 + T cells	Enhanced antigen presentation capacity and cytokine secretion compared to traditional APC lines	MHC class II and co-stimulatory signaling pathways	[216]
iPSC-derived natural killer (NK) cell-engagers	Acute myeloid leukemia (AML)	Selective killing of AML cells through targeted recognition	Improved specificity and potency compared to NK cell-engagers derived from primary NK cells	CD16 and Fc receptor signaling pathways	[485]
iPSC-derived tumor-infiltrating lymphocytes (TILs)	Ovarian cancer	Tumor cell lysis and cytokine production	Higher tumor recognition and effector functions compared to TILs derived from tumor tissues	TCR and co-stimulatory signaling pathways	[232, 233, 387]
iPSC-derived chimeric antigen receptor-natural killer (CAR-NK) cells	Non-small cell lung cancer (NSCLC)	Enhanced killing of NSCLC cells expressing specific antigens	Improved persistence and tumor cell recognition compared to CAR-NK cells derived from peripheral blood	CAR signaling pathway	[486]
iPSC-derived tumor-specific cytotoxic T lymphocytes (CTLs)	Prostate cancer	Generation of antigen-specific CTLs targeting prostate cancer cells	Higher specificity and potency compared to CTLs derived from peripheral blood	TCR and co-stimulatory signaling pathways	[267, 404–406]
iPSC-derived cancer-associated fibroblasts (CAFs)	Pancreatic cancer	Modulation of tumor microenvironment and promotion of antitumor immune response	Enhanced secretion of cytokines and extracellular matrix remodeling compared to traditional CAF lines	TGF-β and NF-κB signaling pathways	[426, 427]
iPSC-derived neoantigen-presenting dendritic cells	Lung cancer	Induction of neoantigen-specific T cell responses	Efficient presentation of personalized neoantigens compared to traditional dendritic cells	MHC class I and co-stimulatory signaling pathways	[216]
iPSC-derived oncolytic viruses	Brain tumors	Selective replication within tumor cells and induction of antitumor immune response	Enhanced tumor tropism and immunogenicity compared to traditional oncolytic viruses	RIG-I and STING signaling pathways	[43]
iPSC-derived tumor-infiltrating lymphocytes (TILs)	Gastric cancer	Tumor cell recognition and secretion of pro-inflammatory cytokines	Improved tumor specificity and effector functions compared to TILs derived from tumor tissues	TCR and co-stimulatory signaling pathways	[50]
iPSC-derived natural killer (NK) cells expressing bispecific killer cell engagers (BiKEs)	Multiple myeloma	Targeted killing of multiple myeloma cells through dual antigen recognition	Increased specificity and cytotoxicity compared to NK cells expressing traditional BiKEs	CD16 and Fc receptor signaling pathways	[273, 298]
iPSC-derived cancer-targeting antibodies	Breast cancer	Binding and neutralization of cancer-specific antigens	Enhanced specificity and affinity compared to antibodies derived from hybridoma cell lines	B cell receptor (BCR) signaling pathway	[114–116]
iPSC-derived cancer vaccines	Prostate cancer	Induction of tumor-specific immune responses	More precise targeting of tumor antigens compared to traditional cancer vaccines	Not applicable	[120]
iPSC-derived tumor-infiltrating lymphocytes (TILs) expressing chimeric antigen receptors (CARs)	Leukemia	Targeted killing of leukemia cells expressing specific antigens	Improved efficacy and persistence compared to TILs or CAR-T cells derived from peripheral blood	CAR signaling pathway	[132]
iPSC-derived tumor-associated neutrophils	Lung cancer	Activation of innate immune response against tumor cells	Enhanced tumor cell phagocytosis and release of cytotoxic granules compared to traditional neutrophil cell lines	Toll-like receptor (TLR) signaling pathway	[216]
iPSC-derived cancer-targeting peptides	Pancreatic cancer	Selective binding and inhibition of cancer cell growth	Higher affinity and specificity compared to peptides derived from synthetic libraries	Not applicable	[133]
iPSC-derived regulatory T cells (Tregs)	Colorectal cancer	Suppression of antitumor immune responses	Enhanced immunosuppressive functions and stability compared to Tregs derived from peripheral blood	TGF-β and IL-10 signaling pathways	[130, 138]
iPSC-derived cancer-targeting nanocarriers	Ovarian cancer	Enhanced delivery of therapeutic agents to tumor cells	Improved tumor targeting and drug release compared to traditional nanocarriers	Not applicable	[139]
iPSC-derived tumor-infiltrating lymphocytes (TILs) expressing T cell bispecific antibodies	Lymphoma	Targeted killing of lymphoma cells through dual antigen recognition	Increased efficacy and specificity compared to TILs or monoclonal antibodies alone	TCR and co-stimulatory signaling pathways	[245, 246]
iPSC-derived cancer-specific antibodies conjugated with cytotoxic payloads	Colorectal cancer	Selective delivery of cytotoxic agents to cancer cells	Higher precision and potency compared to conventional antibody–drug conjugates	Not applicable	[130]
iPSC-derived cancer-targeting exosomes	Liver cancer	Induction of antitumor immune responses and inhibition of tumor growth	Improved stability and specificity compared to exosomes derived from other cell sources	Not applicable	[174, 247–249]
iPSC-derived tumor-infiltrating lymphocytes (TILs) engineered with checkpoint inhibitors	Melanoma	Enhanced antitumor activity and resistance to immune suppression	Improved functionality and persistence compared to TILs alone	TCR and checkpoint signaling pathways	[140, 141]
iPSC-derived cancer-targeting aptamers	Pancreatic cancer	Binding and inhibition of cancer-specific proteins	Higher affinity and specificity compared to aptamers derived from other sources	Not applicable	[147]
iPSC-derived cancer-targeting oncolytic viruses	Pancreatic cancer	Selective replication within tumor cells and induction of antitumor immune response	Enhanced tumor tropism and immunogenicity compared to traditional oncolytic viruses	RIG-I and STING signaling pathways	[147]
iPSC-derived tumor-specific chimeric antigen receptor natural killer (CAR-NK) cells	Lymphoma	Specific killing of lymphoma cells expressing tumor-specific antigens	Improved persistence and safety compared to CAR-T cells derived from peripheral blood	CAR signaling pathway	[245, 246]
iPSC-derived cancer-targeting peptides conjugated with immune checkpoint inhibitors	Breast cancer	Selective targeting of cancer cells and inhibition of immune evasion mechanisms	Enhanced specificity and potency compared to individual agents alone	Not applicable	[114–116]
iPSC-derived tumor-associated B cells	Lung cancer	Modulation of immune response and antitumor activity	Higher antigen presentation capacity and cytokine secretion compared to traditional B cell lines	B cell receptor (BCR) signaling pathway	[216]
iPSC-derived cancer-targeting nanoparticles	Prostate cancer	Targeted delivery of therapeutic agents to prostate cancer cells	Improved tumor specificity and drug release kinetics compared to conventional nanoparticles	Not applicable	[52, 266–268]
iPSC-derived tumor-infiltrating lymphocytes (TILs) expressing enhanced cytokine receptors	Renal cell carcinoma	Augmented cytokine signaling and antitumor activity	Increased cytokine responsiveness and functional potency compared to TILs alone	Cytokine receptor signaling pathways	[301, 315, 316]
iPSC-derived cancer-targeting antibodies conjugated with immune-modulating agents	Pancreatic cancer	Specific binding to cancer cells and modulation of immune response	Enhanced specificity and immune-modulating effects compared to conventional antibody therapies	Not applicable	[236, 238, 311]
iPSC-derived tumor-infiltrating lymphocytes (TILs) expressing bispecific T cell engagers (BiTEs)	Acute lymphoblastic leukemia (ALL)	Targeted killing of ALL cells through dual antigen recognition	Improved efficacy and specificity compared to TILs or BiTEs derived from peripheral blood	TCR and co-stimulatory signaling pathways	[487]
iPSC-derived cancer-targeting microRNAs	Colorectal cancer	Inhibition of cancer cell growth and metastasis	Higher specificity and stability compared to synthetic microRNAs	Not applicable	[130]
iPSC-derived tumor-associated natural killer (NK) cells	Pancreatic cancer	Activation of innate immune response and cytotoxicity against tumor cells	Improved tumor cell recognition and killing capacity compared to traditional NK cell lines	NK cell activating signaling pathways	[147]
iPSC-derived cancer vaccines utilizing CRISPR-Cas9 gene editing	Lung cancer	Induction of tumor-specific immune responses through targeted genetic modifications	Enhanced precision and efficacy compared to traditional cancer vaccines	Not applicable	[144, 145]
iPSC-derived tumor-infiltrating lymphocytes (TILs) expressing immune checkpoint inhibitors	Head and neck cancer	Reversal of immune suppression and enhanced antitumor activity	Improved functionality and checkpoint blockade compared to TILs alone	TCR and checkpoint signaling pathways	[278, 408, 409]
iPSC-derived cancer-targeting dendritic cell vaccines	Brain cancer	Induction of tumor-specific immune responses and activation of cytotoxic T cells	Enhanced antigen presentation and immunogenicity compared to traditional dendritic cell vaccines	Not applicable	[285–289]
iPSC-derived tumor-infiltrating lymphocytes (TILs) engineered with cytokine gene expression	Melanoma	Augmented antitumor activity and cytokine production	Improved persistence and effector functions compared to unmodified TILs	Cytokine signaling pathways	[140, 141]
iPSC-derived cancer-targeting aptamers conjugated with chemotherapy drugs	Ovarian cancer	Selective delivery of chemotherapy drugs to cancer cells	Higher affinity and specificity compared to traditional drug conjugates	Not applicable	[232, 233, 304]
iPSC-derived tumor-associated regulatory T cells (Tregs)	Breast cancer	Suppression of antitumor immune response and modulation of tumor microenvironment	Enhanced immunosuppressive functions and stability compared to traditional Tregs	TGF-β and IL-10 signaling pathways	[109, 214, 215]
iPSC-derived cancer vaccines targeting tumor-specific neoantigens	Colorectal cancer	Generation of immune response against unique tumor neoantigens	Improved specificity and efficacy compared to traditional cancer vaccines	Not applicable	[130, 138]
iPSC-derived tumor-infiltrating lymphocytes (TILs) expressing chemokine receptors	Prostate cancer	Enhanced migration and infiltration into tumor sites	Improved tumor homing and antitumor activity compared to TILs alone	Chemokine signaling pathways	[267, 404–406]

### Utilizing iPSCs for cancer cell differentiation and apoptosis

Another mechanism through which iPSCs contribute to cancer therapy is by directing their differentiation into specific cell types that can target and eliminate cancer cells [25]. Researchers can guide iPSCs to differentiate into immune cells, such as NK cells or T cells, which have the ability to recognize and destroy cancer cells [25]. The iPSC-derived NK cells can be engineered to enhance their anti-cancer properties, such as increasing their cytotoxic activity or improving their targeting capabilities. These modified iPSC-derived NK cells can then be used as a cell-based therapy to directly kill cancer cells figure [131]. Similarly, iPSC-derived T cells can be modified to express CARs that recognize specific cancer cell antigens, enabling them to selectively target and eliminate cancer cells [488]. Furthermore, iPSCs can be used to induce apoptosis, programmed cell death, in cancer cells. By understanding the signaling pathways and molecular changes that drive cancer cell survival, researchers can engineer iPSCs to produce and release therapeutic molecules or microRNAs that promote cancer cell apoptosis [481]. This approach offers a potential strategy for selectively eliminating cancer cells while minimizing harm to healthy cells.

## Current limitations and challenges in iPSC-based cancer therapy

In recent years, iPSCs have emerged as a promising tool in the field of cancer therapy. These cells, which can be derived from adult somatic cells and reprogrammed to exhibit pluripotency, hold great potential for personalized medicine and regenerative therapies [489]. However, despite the significant progress made in this area, there are several limitations and challenges that need to be addressed before iPSC-based cancer therapy can be widely adopted. One of the major challenges in iPSC-based cancer therapy is the technical difficulty associated with generating high-quality iPSCs [490]. The reprogramming process itself is complex and inefficient, often resulting in low-quality iPSCs with genomic abnormalities [491]. These abnormalities can limit the therapeutic potential of iPSCs and may even pose safety risks, such as tumorigenicity [491]. Researchers are actively working on improving the efficiency and quality of iPSC generation. Techniques such as the use of non-integrating reprogramming methods, optimization of culture conditions, and the development of novel reprogramming factors are being explored to enhance the generation of high-quality iPSCs [492, 493]. However, further research is needed to overcome these technical difficulties and ensure the reliable production of iPSCs for cancer therapy. Another significant challenge in iPSC-based cancer therapy lies in the regulatory and safety aspects of using these cells in clinical settings [494]. iPSCs, being a relatively new technology, pose unique regulatory challenges. The regulatory agencies need to establish clear guidelines and standards for the production, characterization, and quality control of iPSCs for cancer treatment [40]. Safety is also a crucial concern when using iPSCs in cancer therapy. As mentioned earlier, iPSCs can harbor genomic abnormalities that could potentially lead to tumorigenicity. Rigorous safety assessments and monitoring protocols should be implemented to minimize the risk of adverse events and ensure patient safety [495]. The use of iPSCs in cancer therapy raises important ethical and legal concerns that need to be carefully addressed [496]. The generation and manipulation of iPSCs involve the use of human embryos or adult somatic cells, which raises ethical questions regarding the source of cells and the potential destruction of embryos [497]. To navigate these ethical challenges, researchers and policymakers must engage in comprehensive discussions and establish clear guidelines regarding the sources of cells, informed consent, and the potential uses of iPSCs in cancer therapy [497]. Striking a balance between scientific advancement and ethical considerations is crucial to ensure the responsible and ethical use of iPSCs in cancer treatment. In addition to the ethical concerns related to the source of cells, there are broader ethical and legal concerns surrounding the use of iPSCs in cancer therapy. These concerns include issues of privacy, consent, and potential commercial exploitation of iPSC technology [498]. The use of iPSCs for personalized cancer therapy requires the collection and storage of patients’ biological materials, including somatic cells. Ensuring the privacy and confidentiality of patients’ data is essential to maintain trust in the healthcare system. Moreover, there is a need to address potential conflicts of interest and ensure equitable access to iPSC-based cancer therapies. Policies and regulations should be in place to prevent the commercial exploitation of iPSC technology and to promote fair and affordable access to these treatments. Generating high-quality iPSCs remains a technical challenge in iPSC-based cancer therapy [499]. The reprogramming process can be affected by various factors, such as the age and quality of the somatic cells used as the starting material [500]. Researchers have observed that iPSCs derived from older donors or from cells with genetic abnormalities tend to have a higher risk of genomic instability and lower differentiation potential [501]. Overcoming these technical difficulties is crucial to ensure the generation of iPSCs that are suitable for use in cancer therapy. Efforts are underway to improve the efficiency and reliability of iPSC generation. Researchers are exploring different reprogramming methods, including the use of small molecules and modified RNA, to enhance the efficiency and quality of iPSC production [502, 503]. Additionally, advancements in gene editing technologies such as CRISPR-Cas9 are being utilized to correct genomic abnormalities in iPSCs, further improving their quality and safety [504]. Regulatory and safety issues are significant challenges that need to be addressed when considering the use of iPSCs in cancer treatment. The regulatory landscape for iPSC-based therapies is still evolving, and regulatory agencies are actively working to establish guidelines and standards [505]. Safety concerns related to iPSC-based cancer therapy include the potential for tumor formation and immune rejection [506, 507]. To mitigate these risks, extensive preclinical studies and rigorous safety assessments are required before iPSC-based therapies can progress to clinical trials [508]. Long-term monitoring and follow-up of patients receiving iPSC-based treatments are also necessary to evaluate their safety and efficacy [507]. Moreover, regulatory agencies need to establish clear criteria for the characterization and quality control of iPSCs used in cancer therapy [509]. This includes ensuring that the iPSCs have the desired pluripotent properties and are free from genetic abnormalities and contaminants [510]. While iPSC-based cancer therapy holds immense promise, there are several limitations and challenges that need to be overcome. Technical difficulties in generating high-quality iPSCs and addressing regulatory, safety, ethical, and legal concerns are crucial areas that require further research and development [511]. Collaboration among researchers, policymakers, and regulatory agencies is essential to navigate these challenges and unlock the full potential of iPSC-based cancer therapy, ultimately leading to improved patient outcomes and personalized treatments in the future.

## The biogenesis of cancer

Cancer is a multistep disease that is characterized by continuous and excessive cell division. Globally, it is the second leading cause of death [16, 17]. Cancer is caused by genetic mutations and epigenetic alterations. During the initial stages of cancer development, normal cells undergo transformation toward a neoplastic state, acquiring new capabilities such as unlimited replicative potential, resistance to cell death, and stimulation of angiogenesis. Biogenesis refers to the process by which normal cells become cancerous [18]. This article will explore the various aspects of tumorigenesis, including the cancer cell-of-origin hypothesis, the interplay between genetics, epigenetics, and environmental factors, and the role of stem cells in tumorigenesis.

### Overview of tumorigenesis

During the past 20 years, we have gained a great deal of knowledge about how various cancers develop at the molecular and cellular levels [19]. Variability in response to anti-cancer drugs among cancer patients can be elucidated by genetic molecular features such as mutations and copy number changes, and DNA methylation [20]. In addition to improving our understanding of this process, identifying genes and pathways involved will also help us to develop new therapeutic targets [21]. In cancer, chromosomal changes and genes are disrupted through genomic approaches. It is recognized that tumorigenesis is a complex process that involves progressive transformations triggered by multiple factors and it is regulated by both oncogenes and tumor suppressor genes. Several growth-promoting and growth-restricting mechanisms regulate the cell cycle which is crucial for proper division and propagation. A disruption of this regulation may lead to uncontrolled proliferation and genomic instability, which may trigger the development of cancer [22]. Activation of oncogenes leads to tumorigenesis, which controls cell proliferation and apoptosis. They can be activated by structural alterations resulting from mutation or gene fusion. Additionally, tumor suppressor genes (TSG) encode proteins that regulate cell proliferation negatively. They have included in two classes: the “caretakers” of the genome (or Type I), which are DNA repair genes that protect the genome from mutations (XPB, MSH2, etc.), and the gatekeepers (or Type II), which avert cancer through direct control of cell growth (p53, p16, etc.).The inactivation of tumor suppressor genes by loss or mutation is a vital step for the development of tumors [23]. Besides genetic alterations, epigenetic mechanisms contribute to the development of malignant phenotypes, according to growing evidence [24]. The study of epigenetics, which is dynamic and susceptible to environmental factors, is concerned with mechanisms that change gene expression without altering the primary DNA sequence. Epigenetic processes are heritable and reversible and consist of changes in DNA methylation, histone modifications, small noncoding microRNAs (miRNA), and nucleosome remodeling [25]. The alteration of cellular methylation status by a specific methyltransferase might explain the differences in the probability of malignant transformation [26]. Tumor cells in different tissue with a wide range of patterns of histone modification, genome-wide or in individual genes, demonstrate the presence of epigenetic heterogeneity at a cellular level [27]. The combined action of multiple epigenetic factors results in tumorigenesis. For example, the repression of tumor suppressor genes is caused by the methylation of DNA CpG islands with hypoacetylated and hypermethylated histones [28]. Several hallmarks of epigenetic events have been identified during gene silencing, including histone H3 and H4 hypoacetylation, histone H3K9 methylation, and cytosine methylation [29].

### Overview of the cancer cell-of-origin hypothesis

The cancer cell-of-origin hypothesis is a fundamental concept in cancer research that aims to identify the specific type of cell from which a tumor originates [512]. This hypothesis suggests that certain cancers arise from a small subset of cells within a tissue or organ that possess unique properties, enabling them to acquire the genetic and epigenetic alterations necessary for tumor initiation and progression [513]. According to recent studies published in renowned scientific journals, researchers have made significant progress in elucidating the origins of various types of cancer [512]. Some studies focused on breast cancer, revealing that a small population of mammary stem cells, which normally aid in the maintenance and repair of breast tissue, can undergo genetic mutations that transform them into cancer-initiating cells. These findings support the cancer cell-of-origin hypothesis, highlighting the importance of targeting these specific cells for effective treatment strategies [514]. Another study investigated the origins of brain tumors, specifically glioblastoma, one of the most aggressive and lethal forms of brain cancer [515]. By analyzing human brain tissue samples and using advanced genetic sequencing techniques, scientists identified a subset of neural stem cells as the likely cell-of-origin for glioblastoma. These cells possess the ability to self-renew and differentiate into various types of brain cells, making them susceptible to acquiring oncogenic mutations that lead to tumor formation [514]. In addition to breast and brain cancer, the cancer cell-of-origin hypothesis has also been explored in other malignancies [516]. Research on colorectal cancer has suggested that a small population of intestinal stem cells may give rise to the development of adenomas, the precursor lesions of colorectal cancer [517]. Similarly, in skin cancer, studies have implicated epidermal stem cells as the cell-of-origin for various types of skin tumors, including basal cell carcinoma and squamous cell carcinoma [518]. Understanding the cell-of-origin for different cancers is not only crucial for unraveling the molecular mechanisms underlying tumor development but also has significant implications for personalized medicine and targeted therapies [519]. By identifying and characterizing the unique properties of cancer-initiating cells, researchers can develop strategies to specifically target and eliminate these cells, thus preventing tumor recurrence and improving patient outcomes [518]. Advancements in single-cell sequencing technologies have played a pivotal role in advancing our understanding of the cancer cell-of-origin hypothesis. These cutting-edge techniques enable researchers to analyze individual cells within a tumor and decipher their genomic and epigenomic landscapes [520]. By comparing the genetic profiles of cancer-initiating cells with their normal counterparts, scientists can identify key genetic alterations that drive tumor initiation and progression [521]. Moreover, the cancer cell-of-origin hypothesis has prompted further investigation into the role of the tumor microenvironment in tumor development [521]. Emerging evidence suggests that interactions between cancer-initiating cells and their surrounding microenvironment, including immune cells, fibroblasts, and blood vessels, play a critical role in promoting tumor growth and metastasis [522]. Targeting these interactions may provide novel therapeutic opportunities for cancer treatment. The cancer cell-of-origin hypothesis has revolutionized our understanding of tumor initiation and progression [523]. By identifying the specific cells from which cancers originate, researchers have made significant strides in deciphering the underlying mechanisms of various malignancies. This knowledge has paved the way for the development of personalized medicine approaches that target cancer-initiating cells, offering new hope for improved treatment outcomes [524]. Continued research in this field holds promise for further advancements in cancer prevention, diagnosis, and therapy, ultimately leading to better patient care and survival rates [525].

The relationship between cell-of-origin and cancer stem cells is a fundamental aspect of cancer biology. While the cell-of-origin initiates the oncogenic process, CSCs sustain and drive tumor growth, metastasis, and treatment resistance [512]. Recognizing the roles and characteristics of both cell types is vital for advancing our understanding of cancer and developing more precise and effective treatments. Cell-of-origin refers to the normal cell type from which cancer originates within the body [525]. Cancer typically arises when these normal cells accumulate genetic mutations or epigenetic changes that lead to uncontrolled growth and the development of a malignant tumor. The specific cell-of-origin can vary depending on the type of cancer and the tissue or organ in which it develops [521]. For instance, in lung cancer, the cell-of-origin may be a normal lung epithelial cell, while in breast cancer, it could be a mammary gland cell. The cell-of-origin plays a crucial role in shaping the characteristics of the resulting cancer, including its growth patterns and response to treatment. Cancer stem cells (CSCs), on the other hand, represent a specialized subset of cells within a tumor [526]. These cells are distinguished by their unique ability to self-renew and give rise to various cell types found within the tumor [525]. CSCs are thought to derive from either a small population of cancer cells that acquire additional mutations or from normal stem cells within the tissue that undergo dedifferentiation. They are often found at the apex of the tumor hierarchy and are responsible for driving tumor growth, initiating new tumors (tumor initiation), promoting metastasis, and conferring resistance to conventional cancer therapies [526]. One of the critical distinctions between cell-of-origin and CSCs lies in their roles in tumorigenesis [512]. The cell-of-origin is the initial cell that undergoes oncogenic events, leading to the formation of a cancerous lesion. In contrast, CSCs are responsible for the long-term maintenance of the tumor, playing a central role in its sustained growth and progression. Because CSCs possess self-renewal abilities and are highly resistant to treatment, they are often the culprits behind cancer recurrence after initial therapy [526]. Understanding the clinical implications of the cell-of-origin and CSCs is essential for cancer research and treatment strategies. Identifying the cell-of-origin provides insights into the tumor’s characteristics and behavior, helping clinicians tailor treatment approaches. For example, knowledge of the cell-of-origin can inform decisions about targeted therapies or treatments designed to eliminate the bulk of tumor cells derived from the cell-of-origin. In contrast, targeting CSCs has become a focal point in cancer therapy development [525]. Strategies aimed at eradicating or inhibiting CSCs are crucial for preventing tumor relapse and improving long-term treatment outcomes. By selectively targeting these stem-like cells within a tumor, researchers and clinicians hope to undermine the tumor’s ability to regenerate and resist treatment, ultimately leading to more effective cancer therapies [521].

### Understanding the interplay between genetics, epigenetics and environment in tumorigenesis

There is increasingly evidence supporting that genetic and epigenetic mechanisms do not operate separately during tumorigenesis, they work together and are intertwined and take advantage of each other. Additionally, epigenetic changes may occur due to chance or as a result of environmental factors [30]. Ultimately, gene expression and abnormal phenotypes influenced by genetics, epigenetics and environment [31]. Although the genetic road to cancer or genome instability is relatively straightforward which occurred by mutation of tumor suppressors and/or oncogenes causes either a loss or gain of function and abnormal expression. Epigenetic pathways determine tumorigenesis through integrating numerous epigenetic variations, which are much more complex [31]. Epigenetics is based on the idea that interaction between the environment and the epigenome can alter phenotypes and contribute to disease susceptibility. It is noted that these changes could be transmitted down through generations [32]. Epigenetics is susceptible to environmental stressors. For example exposure to metals, chemical and xenobiotic compounds, air pollution, benzene, organic pollutants, and radiation can induce mutations that contribute to the development of cancer especially during embryonic stages, environmental factors have a more crucial impact on the genome and even can increase the risk of cancer in F_1_, F_2_ and F_3_ generation [33]. The repression of tumor suppressor genes can also be induced by unhealthy habits, diet and pharmacological agents apart from chemical and physical environmental contributors. Several studies have shown how diet and food availability affect the epigenome in humans and how these epigenetic changes may be involved in several diseases in adulthood. There is an association between folate intake in the diet and epigenetic status in mammals [34] and has been related to methylation changes in colon cancer [35] and hyperhomocysteinemia [36]. Moreover, pharmacological treatments including those used for epilepsy, bipolar disorder, serious depression, migraine, and schizophrenia, complementary treatment for latent HIV infection can also induce genome-wide epigenetic changes [37]. In this regard, it remains unknown what part of the changes are caused by the interaction of the environment with the epigenome and what part are the result of just genetics. In order to, further research should focus on understanding the causes of these changes. Several of these questions will be answered in the future by next-generation technologies.

### The role of stem cells in tumorigenesis

**Fig. 9:**
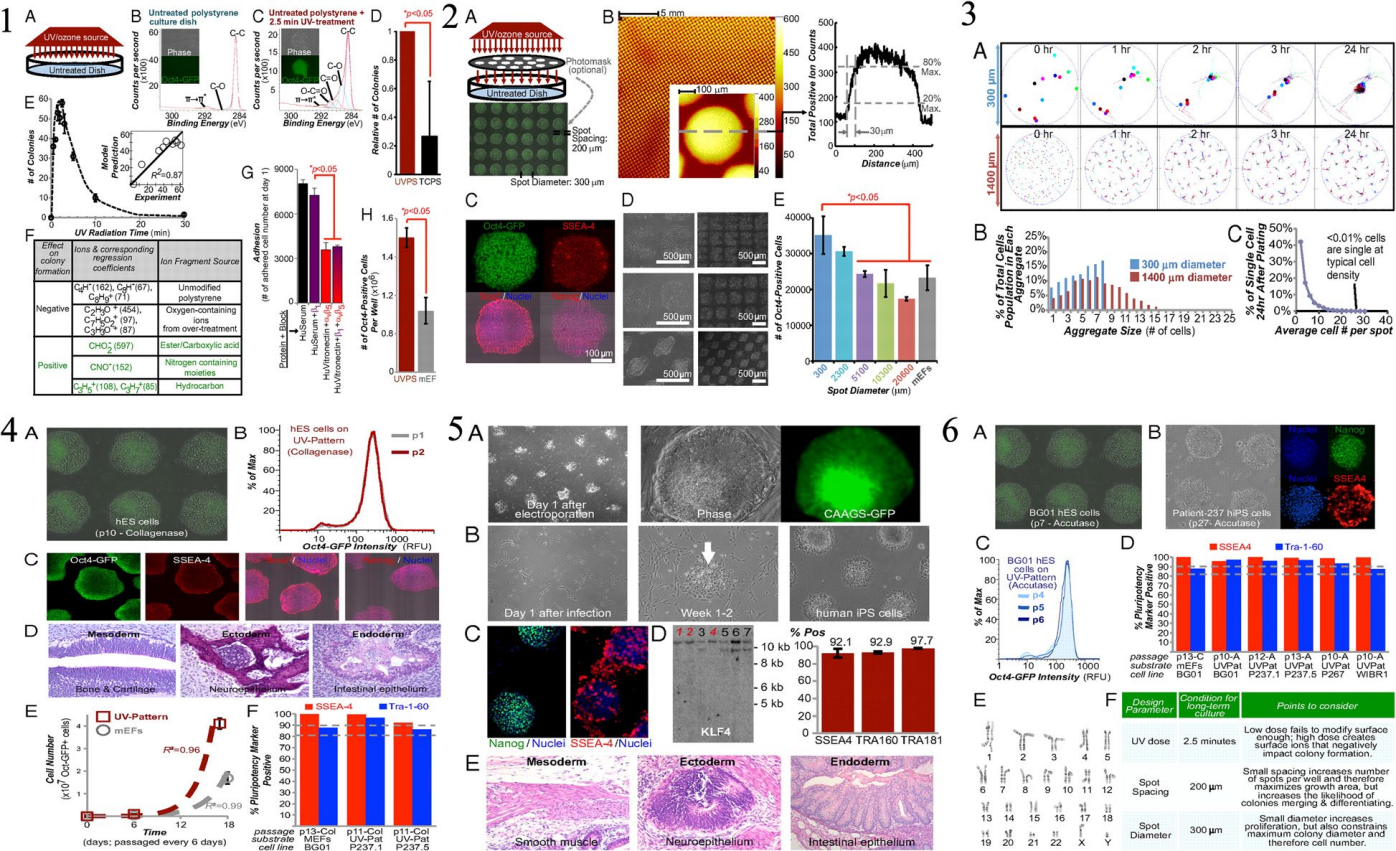
**1** The comparison between the growth of undifferentiated human pluripotent stem cells on chemically optimized substrates and feeder-containing substrates. The figure includes multiple panels showing different aspects of the experiment. Panel A depicts a schematic diagram of the UV treatment process, while panels B and C display XPS spectra indicating the surface chemical functionality of a polystyrene culture dish before and after UV treatment. Phase-contrast and fluorescence images of transgenic Oct4-GFP hESCs on these surfaces are shown, with bright green fluorescence indicating strong expression of the pluripotency marker Oct4. Panel D presents the relative number of hESC colonies on UVPS (UV-treated polystyrene) compared to conventional TCPS (standard tissue culture polystyrene) on the seventh day after cell seeding. Panel E demonstrates colony formation on virgin polystyrene treated with various UV doses, and the inset shows the prediction of colony numbers based on a PLS model. In panel F, the PLS model is used to identify surface ions that either support or inhibit hESC colony formation based on ToF–SIMS data. Panel G presents the number of adhered cells after 24 h of culture on UVPS coated with either human serum or recombinant human vitronectin, along with integrin-blocking antibodies. The results show that blocking αvβ5 integrin reduces adhesion, while blocking β1 integrin has minimal effect. Finally, panel H compares the number of undifferentiated Oct4-GFP-positive hESCs per well on UVPS and standard mouse embryonic feeder (mEF)-containing substrates after seven days of culture. UVPS coated with vitronectin is represented by the red bar, while the gray bar represents the mEF-containing substrates. The error bars in all panels indicate 95% confidence intervals, and the experiments were conducted with a sample size of three. **2** The optimization of polystyrene substrates through chemical and geometrical modifications. Panel A provides a schematic representation of the UV treatment process, which can be controlled spatially by inserting a photomask between the UV source and the dish. An overlay of phase-contrast and fluorescent images shows transgenic Oct4-GFP hESC cultures on a UV-patterned polystyrene substrate. The substrate, referred to as UV-Pattern, was coated with FBS (fetal bovine serum). Panel B presents a Time-of-Flight Secondary Ion Mass Spectrometry (ToF–SIMS) scan of the UVPS (UV-patterned polystyrene) surface after patterning with a photomask. The scan reveals the intensity of all positive ions, with different colors indicating varying intensities. The profile demonstrates a resolution of 30 μm between the points where the ion intensity changes from 20 to 80%. The abbreviation “Max.” stands for maximum. Panel C shows immunostaining of pluripotency markers in cells cultured on the UV-Pattern described in Panel A. Panel D demonstrates the possibility of patterning human embryonic stem cells (hESCs) or human-induced pluripotent stem cells (hiPSCs) using different geometries, suggesting versatility in the patterning process. Panel E presents the results of the experiment, showing the number of undifferentiated Oct4-GFP-positive cells in each well after 7 days of culture. The measurement was performed using flow cytometry on constant area patterns. Each well initially contained 15,000 cells, and the cumulative UV-treated area per well remained the same across all patterns. Error bars represent the 95% confidence intervals, and the surfaces were precoated with 20% bovine serum. The cells were seeded in the presence of a ROCK inhibitor for the first 8–12 h. **3** The results of simulating cell behavior on substrates patterned with UV light. In the first part (A), snapshots of human embryonic stem cells (hESCs) or hiPSCs are shown on spots with diameters of 300 and 1,400 μm. These snapshots were taken during the simulation and demonstrate that the majority of cells aggregate within 3 h, which is consistent with observations made during live imaging. The second part (B) presents the distribution of cells within each aggregate as predicted by the cell migration model. This prediction is shown for two different patterned spot diameters: 300 μm and 1,400 μm. It is important to note that no ROCK inhibitor was present in the media during these simulations. Lastly, part (C) provides information about the percentage of cells that exist as single cells, not paired or in colonies, as a function of cell density. When cells are seeded at a typical density used in routine cell culture (60,000 cells per well in a 6-well plate), the data indicates that less than 0.01% of cells remain as single cells. **4** The use of a UV-patterned substrate to facilitate long-term culture of cells. Panel A shows an overlay of phase-contrast and fluorescent images of transgenic Oct4-GFP BG01 hESC cultures on the UV-patterned substrate after 10 passages using collagenase dissociation. Panel B presents flow cytometry data of cells after two consecutive passages on the UV-patterned substrate, indicating the relative fluorescent units (RFU) and maximum (Max) values. The passage number (p) is also mentioned. Panel C displays immunostaining results for pluripotency markers in cells grown on the UV-patterned substrate. Panel D exhibits the formation of teratomas in immunodeficient mice by cells cultured on the UV-patterned substrate. Hematoxylin and eosin (H&E) staining of the teratoma reveals the presence of tissues representing all three germ layers. Panel E depicts the number of undifferentiated Oct4-GFP-positive hESCs over three passages using accutase on the UV-patterned substrate (red) compared to standard mouse embryonic feeder (mEF)-containing substrates (gray) when seeded with 24,000 cells per well of a six-well plate. The error bars indicate 95% confidence intervals, and the high R2 coefficient of determination suggests a good fit to the exponential growth model. Panel F shows flow cytometry data for pluripotency markers SSEA-4 and Tra-1–60 after more than 10 consecutive passages on the UV-patterned substrate for two different hiPSC lines, P237.1 and P237.5. Collagenase passaging (Col) is mentioned. In the case of transgenic Oct4-GFP BG01 hESCs passaged on MEFs, only GFP-positive cells were analyzed for Tra-1–60 and SSEA-4 expression, excluding MEFs from the analysis. **5** The utilization of UV-patterned substrates to facilitate the reprogramming and gene modification of human pluripotent stem cells. In panel A, phase-contrast images display BG01 human embryonic stem cells (hESCs) on UV-patterned substrates with specific dimensions. These cells were subjected to electroporation with CAAGS-GFP targeting and ZFN plasmids. Following electroporation, the cells were initially cultured in the presence of ROCK inhibitor. A successful targeted clone was then transferred to mouse embryonic fibroblasts (mEFs) and exhibited a high level of green fluorescent protein (GFP) expression after more than two months of culture. Panel B includes phase-contrast and immunostained images of “patient-237” fibroblasts on UV-patterned polystyrene. These fibroblasts were infected with a modified version of the pHAGE-STEMCCA vector, which contains loxP sites for Cre-mediated excision. The patterned surface was coated with human serum, enabling fibroblasts to adhere to the untreated areas of the dish. Over a period of four weeks, the fibroblasts underwent morphological changes and formed colonies of hiPSCs on the UV-patterned substrates. Panel C depicts the immunostaining of pluripotency markers in the patient-237 hiPSC line grown on the UV-patterned substrate. In panel D, Southern blot analysis of genomic DNA from various patient-237 hiPSC lines is shown, focusing on the Klf4 gene. The analysis reveals different bands representing the presence or absence of the reprogramming vector. The red-labeled cell lines indicate successful excision of the reprogramming vector upon Cre-recombinase expression. The loss of specific viral KLF4 bands indicates the isolation of vector-free hiPSCs through clonal selection. The accompanying bar graph illustrates the percentage of cells positive for pluripotency markers SSEA-4, TRA-1–60, and TRA-1–81, as determined by flow cytometry in a vector-free patient-237 hiPSC line after two passages on the UV-patterned substrate. Error bars represent the 95% confidence intervals based on three replicates. “Pos” denotes positive. Panel E showcases teratoma formation in immunodeficient mice resulting from the injection of vector-free hiPSCs that were reprogrammed and cultured on the UV-patterned substrate. Hematoxylin and eosin (H&E) staining of the teratoma reveals the presence of tissues representative of all three germ layers. **6** The use of a UV-patterned substrate to facilitate the transfer of individual human pluripotent stem cells. In panel A, images show BG01 human embryonic stem cells (hESCs) cultured on the “UV-Pattern” substrate after 7 and 27 passages using single-cell accutase dissociation. The image at passage 7 contains a fluorescent overlay indicating high expression of the Oct4-GFP marker. Panel B displays patient-237 hiPSCs at passage 27, with immunostaining indicating expression of the pluripotency marker Nanog (green) in all cell nuclei and high expression of SSEA-4 (red). The surfaces of the substrates were coated with 20% bovine serum, and cells were seeded in the presence of a ROCK inhibitor for the initial 8–12 h. Panel C presents flow cytometry results of BG01 hESCs with the Oct4-GFP reporter after three consecutive passages on the UV-Pattern substrate using accutase. Panel D shows flow cytometry results for pluripotency markers SSEA-4 and Tra-1–60 in cells from five different cell lines after more than 10 consecutive passages on the UV-Pattern substrate. The letter “A” denotes accutase-mediated passaging. In the case of transgenic Oct4-GFP BG01 cells passaged on mouse embryonic fibroblasts (mEFs), only GFP-positive cells were analyzed for Tra-1–60 and SSEA-4 expression, excluding mEFs from the analysis. Panel E demonstrates that patient-237 hiPSCs propagated on the UV-Pattern substrate for over 5 months (27 passages) maintained a normal 46XY karyotype. Lastly, panel F provides the design parameters used to develop the UV-treated culture system for human pluripotent stem cells. Reprinted from [527] with permission from the PNAS

Stem cells have been used for over 30 years for cancer treatment via tissue regeneration and as delivery vehicles. Research communities have been directed towards advancement in the field of cancer research following the recent introduction of cancer stem cells as the backbone of cancer development [7]. Stem cells are undifferentiated and have the ability to self-renew and proliferate for longer periods, as well as produce multiple types of cells [38]. In a recent investigation led by Saha et al., the primary focus was on the development of specially modified surfaces to improve the cultivation of human pluripotent stem cells (hPSCs) within a completely defined environment [527]. The study’s objective was to address the shortcomings associated with traditional culture systems reliant on feeder cells and establish a standardized and reproducible platform for hPSC cultivation**. **Figure 9 underscores the crucial role of chemically optimized surfaces in fostering the growth of more undifferentiated hPSCs compared to substrates with feeder cells, underscoring the superiority of these engineered surfaces. Figure 9 illustrates the physical changes applied to the polystyrene substrates, encompassing both chemical and geometrical modifications, which were pivotal in enhancing their performance. To further advance the engineered surfaces, the study employed computational modeling, as depicted in Fig. 9, to simulate and predict cell behavior on substrates featuring UV-patterned spots, aiding in the design and enhancement of these surfaces. Figure 9 demonstrates the successful long-term cultivation of stem cells on these UV-patterned substrates, underscoring their suitability for maintaining hPSCs in a healthy, undifferentiated state. Additionally**, **Fig. 9 provides experimental findings and visual proof supporting the effective reprogramming and genetic modification of hPSCs utilizing the UV-patterned substrates, highlighting their potential utility in disease modeling and personalized medicine applications. Finally, Fig. 9 illustrates the utilization of UV-patterned substrates to facilitate the transfer of individual hPSCs, further emphasizing the practical advantages offered by these engineered surfaces. Oncogenes and anti-oncogenes play a key role in initiation of cancer which is followed by the conversion normal stem cells into cancer cells under certain environmental conditions [39]. In general, stem cells reveal various levels of differentiation potential, starting with totipotency, pluripotency, multipotency, oligopotency and finally unipotency/monopotency [528]. The role of stem cells in tumorigenesis encompasses the concept of Cancer Stem Cells (CSCs), which represent a subset of cancer cells sharing characteristics with normal stem cells [247]. CSCs play a pivotal role in driving uncontrolled tumor progression through their abilities in self-renewal [528]. Although it’s worth noting that unlike normal stem cells, not all CSCs exhibit pluripotent differentiation potential, which refers to the capacity for differentiation into all three germ layers [247]. Cancer stem cells are hypothesized to cause metastasis and resistance to therapy, as well as post-operative recurrences. As a result, targeting CSCs may provide new treatments for cancer patients [42]. Recently, many molecular mechanisms have been elucidated that explain tumorigenesis in cancer and stem cell self-renewal [43]. Abnormal activation of signaling pathways is involved in tumor pathogenesis and plays critical roles in growth, progression, and relapse of cancers. The following are signaling cascades that are frequently dysregulated in cancer: Ras/Raf/MEK/ERK (MAPK) as a major determinant in the control of proliferation, survival, and differentiation. And the PTEN/PI3K/AKT/mTOR pathways. Activating mutations of Ras and Raf frequently leading to activation of their downstream targets MEK1/2 and ERK1/2 [44]. Similarly, in the PI3K/AKT/mTOR pathway, mutation/inactivation of PTEN resulting in activation of PI3K and its downstream targets AKT and mTOR [45]. Wnt signaling plays a crucial role in regulating endogenous stem cells as well as tissue development and homeostasis. Cancer stem cells are influenced by abnormal Wnt signaling, which directly contributes to the development and maintenance of many cancers [529]. The Hedgehog (Hh) signaling pathway with three proteins which is involved in activation (Hedgehog (Hh) ligand, Patched (Ptch) and Smoothened (Smo)) is a conserved evolutionary pathway that transmits signals from the cell membrane to the nucleus. It is critical for the regeneration of tissue and is normally inactive or poorly active. The Hh signaling pathway may be involved in various stages of carcinogenesis, as well as in early tumor stages and metastatic tumors [47]. Besides being masses of malignant cells, tumorigenesis is also affected by changes in the tumor microenvironment (TME). Tumor microenvironments are formed by interactions between malignant and non-transformed cells like endothelial and immune cells, MSCs, and fibroblast-like stroma cells, the tumor vasculature and lymphatics, as well as fibroblasts, pericytes and sometimes adipocytes [48]. Essentially, this microenvironment contribute to maintain tumor phenotypes by triggering self-renewal of CSCs, stimulating angiogenesis, and recruiting cells that produce additional factors that drive metastasis and invasiveness of tumor cells [49]. Over several decades, cancer biomarkers like long non-coding RNAs as one of the most important regulatory factors can be used effectively for diagnosis, therapy, and prognosis via their role in identification cancer stem cells and their related microenvironments. They are detectable in liquid biopsy samples such as plasma, saliva, and urine [50]. In both developed and developing countries, cancer remains a significant economic and social burden. It is well known that cancer contributes to increased mortality, poor health, and high healthcare costs in the long run. Therefore, further research is imperative to fully understand the mechanisms underlying tumorigenesis and to develop novel therapeutic strategies to promote the health and well-being of cancer patients [51].

## The iPSCs as a model for tumorigenesis

The iPSCs have emerged as a valuable model for studying tumorigenesis. Their ability to differentiate into cancer cells, their genetic fidelity to patient-derived cells, and their capacity to recapitulate tumorigenic properties make them an ideal tool for investigating the complex processes underlying tumor initiation, progression, and response to therapy. iPSCs offer a unique opportunity to study the molecular and genetic changes occurring during tumorigenesis, providing insights into the key factors driving tumor development [530]. As research in this field advances, iPSCs hold great promise for advancing our understanding of cancer biology and paving the way for personalized and targeted cancer therapies [531]. Moreover, iPSCs offer the advantage of being able to model the early stages of tumorigenesis, which is often challenging to study using traditional cancer cell models [532]. By inducing the differentiation of iPSCs into specific cell lineages relevant to the type of cancer being investigated, researchers can gain insights into the initial cellular events that lead to tumor formation [532]. This includes the acquisition of cancer-specific mutations, epigenetic modifications, and alterations in signaling pathways [533]. iPSCs can provide a time-resolved snapshot of the molecular changes occurring during early carcinogenesis, offering a valuable tool for identifying novel biomarkers and potential therapeutic targets [534, 535]. In addition to their potential in studying cancer initiation, iPSCs can be employed to investigate the dynamic nature of tumor progression[536]. By generating iPSC-derived cancer cells at different stages of tumor development, researchers can observe and analyze the molecular changes that occur as the cancer progresses [537]. This longitudinal approach enables the identification of key genetic and epigenetic events driving tumor growth, invasion, and metastasis [538]. Furthermore, iPSCs can be used to model the heterogeneity observed within tumors, allowing researchers to study the subpopulations of cancer cells with distinct properties, such as stem-like cells or cells with drug-resistant phenotypes [539]. This information is crucial for developing targeted therapies that can effectively eradicate different cancer cell populations. The use of iPSCs in tumorigenesis research also extends to the field of drug discovery and personalized medicine [540]. iPSCs derived from patients with specific types of cancer can be employed in drug screening assays to evaluate the efficacy and toxicity of potential therapeutic agents [541]. This approach holds promise for developing personalized treatment strategies, as iPSCs can be used to predict patient-specific responses to different drugs. By analyzing the drug response profiles of iPSC-derived cancer cells, researchers can identify individualized treatment regimens tailored to each patient’s unique genetic and cellular characteristics, ultimately improving treatment outcomes [540]. While the potential of iPSCs in tumorigenesis research is immense, several challenges and limitations need to be addressed. One challenge is the efficient and reliable generation of iPSCs from patient samples, as this process can be time-consuming and may have variable success rates. Additionally, ensuring the faithful differentiation of iPSCs into the desired cancer cell types can be complex, requiring the optimization of differentiation protocols [542]. Furthermore, the long-term stability and genetic integrity of iPSCs and their differentiated derivatives need to be carefully monitored to avoid unintended genetic or epigenetic changes that could impact the reliability of the model [542]. The iPSCs represent a powerful and versatile model for studying tumorigenesis. Their ability to recapitulate tumorigenic properties, investigate molecular and genetic changes, and model the heterogeneity of tumors makes them a valuable tool for advancing our understanding of cancer development and progression. Furthermore, iPSCs have the potential to revolutionize drug discovery and personalized medicine by enabling the development of tailored therapies based on individual patient characteristics[543]. As researchers continue to overcome challenges and refine techniques in iPSC research, the promising potential of iPSCs for tumorigenesis and therapy will be further realized, paving the way for improved diagnostics, treatments, and patient outcomes in the field of oncology.

### The potential of iPSCs to study tumorigenesis

iPSCs have emerged as a promising resource for delving into the origins of cancer, a multifaceted ailment stemming from a combination of genetic, epigenetic, and environmental influences [532].

**Fig. 10:**
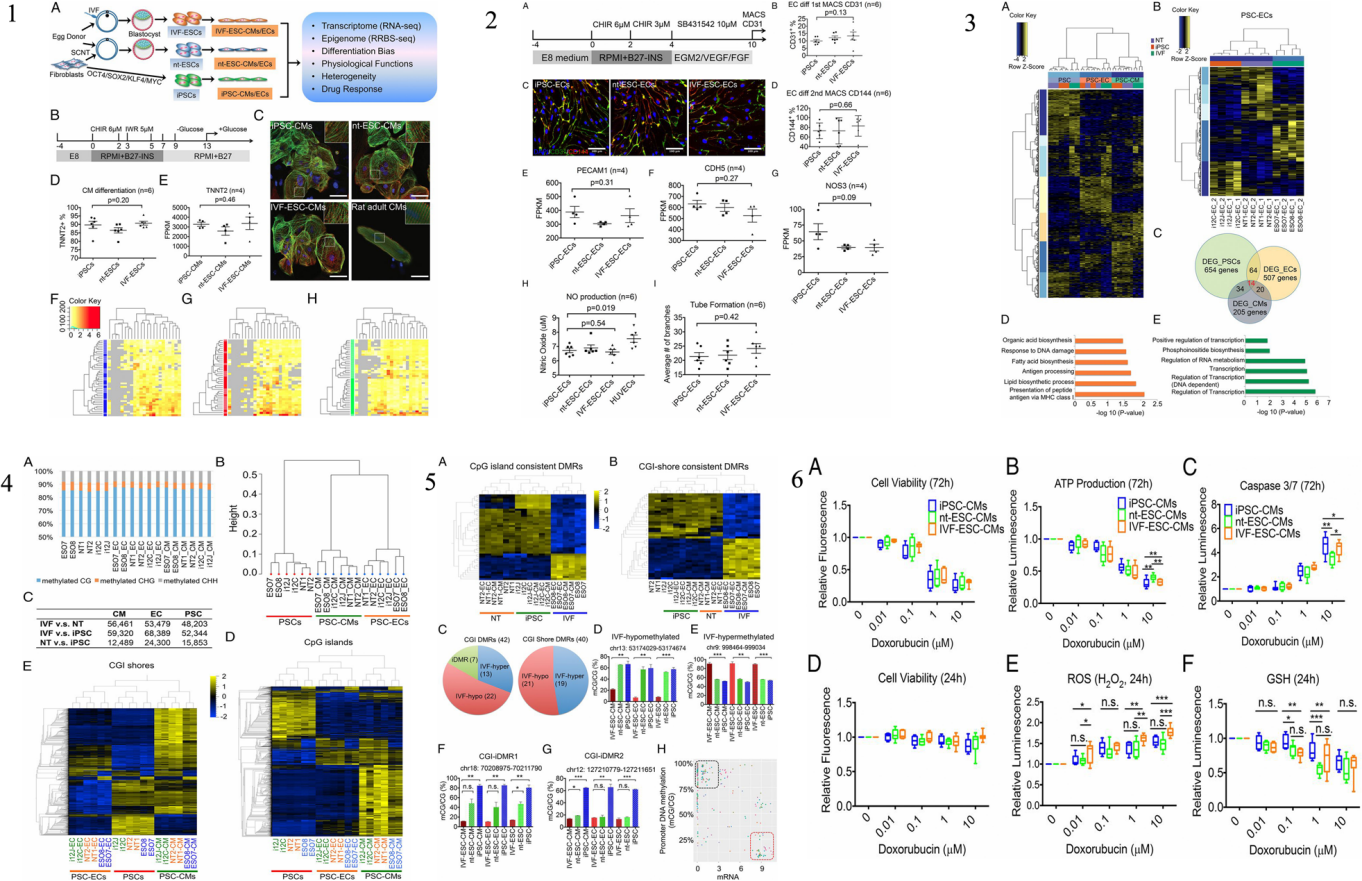
**1** The process of differentiating human iPSCs, nuclear transfer embryonic stem cells (nt-ESCs), and in vitro fertilization embryonic stem cells (IVF-ESCs) into cardiac cells. Panel (A) presents an overview of the experimental design used in the study. Panel (B) illustrates a monolayer cardiac differentiation protocol facilitated by small molecules. Panel (C) shows the sarcomere structures of pluripotent stem cell-derived cardiac cells (PSC-CMs) and rat adult cardiac cells, stained for cardiac troponin T (green), α-actinin (red), and the nuclei counterstained with DAPI (blue). The scale bars represent 25 μm, and the magnification is 600 × . Panel (D) quantifies the efficiency of cardiac differentiation by calculating the percentage of cells positive for TNNT2 (cardiac troponin T) using flow cytometry. Panel (E) compares the expression of TNNT2 in iPSC-derived cardiac cells (iPSC-CMs), nt-ESC-derived cardiac cells (nt-ESC-CMs), and IVF-ESC-derived cardiac cells (IVF-ESC-CMs). Panels (F–H) depict the heterogeneity of cardiac cells derived from different types of pluripotent stem cells using single-cell quantitative polymerase chain reaction (qPCR) analysis. Blue, red, and green colors represent iPSC-CMs, nt-ESC-CMs, and IVF-ESC-CMs, respectively. Heavy and light colors indicate two different cell lines within each category. Each row represents a single cell, while each column represents a single gene. The color key applies to panels F–H. Statistical analysis using one-way ANOVA was performed, and the error bars represent the standard error of the mean (SEM). **2** The generation of endothelial cells (ECs) from various types of pluripotent stem cells (PSCs) using different methods. In (A), a protocol involving small molecules is shown for inducing endothelial differentiation. The effectiveness of the differentiation process is evaluated in (B) by determining the percentage of CD31 + cells at day 12 of differentiation. Representative immunofluorescence staining of PSC-derived ECs using CD31 and CD144 antibodies is presented in (C), with the nuclei counterstained using DAPI. (D) compares the maintenance of endothelial characteristics among induced pluripotent stem cell-derived ECs (iPSC-ECs), nuclear transfer embryonic stem cell-derived ECs (nt-ESC–ECs), and in vitro fertilization-derived ECs (IVF-ESC-ECs) by measuring the percentage of CD144 + cells. No significant differences were observed among these cell types. The expression levels of EC-specific marker genes, PECAM1 (E), CDH5 (F), and NOS3 (G), were similar in iPSC-ECs, nt-ESC–ECs, and IVF-ESC-ECs. The production of nitric oxide by PSC-ECs and human umbilical vein endothelial cells (HUVECs) is shown in (H), while (I) presents the average number of branches in tubes formed by PSC-ECs. **3** The results obtained from analyzing the global gene-expression profiles of pluripotent stem cells (PSCs), PSC-derived cardiomyocytes (PSC-CMs), and PSC-derived endothelial cells (PSC-ECs) using RNA sequencing (RNA-seq). A) The differentially expressed genes (DEGs) between PSCs, PSC-CMs, and PSC-ECs were clustered using unsupervised hierarchical clustering (with a statistical significance threshold of *q* < 0.1). B) PSC-ECs were grouped together based on the specific reprogramming approaches used to generate the iPSCs (i12C, i12J), non-transgenic embryonic stem cells (nt-ESCs) (NT1, NT2), and in vitro fertilization-derived embryonic stem cells (IVF-ESCs) (ESO7, ESO8) (with a statistical significance threshold of *q* < 0.1). C) The number of DEGs identified in PSCs, PSC-CMs, and PSC-ECs due to the different reprogramming approaches is shown, with overlapping regions indicating the number of consistent DEGs shared among the different cell types. D) Gene ontology (GO) analysis was performed to identify enriched functional terms for the DEGs between iPSC-CMs, nt-ESC-CMs, and IVF-ESC-CMs (with a statistical significance threshold of *P* < 0.05). E) GO terms associated with the DEGs in ECs derived from iPSCs, nt-ESCs, and IVF-ESCs were identified using GO analysis (with a statistical significance threshold of *P* < 0.05). **4** The results obtained from analyzing the global DNA methylome of pluripotent stem cells (PSCs), PSC-derived cardiomyocytes (PSC-CMs), and PSC-derived endothelial cells (PSC-ECs) using a technique called RRBS-seq. A) This part shows the percentages of different types of methylated cytosines (mCG, mCHG, and mCHH) among all observed 5-methylcytosines in PSCs, PSC-CMs, and PSC-ECs.B) An unsupervised hierarchical clustering analysis is performed based on the global CpG methylation levels of PSCs, PSC-CMs, and PSC-ECs. The clustering groups include iPSCs, non-transgenic embryonic stem cells (nt-ESCs), in vitro fertilization-derived ESCs (IVF-ESCs), and their respective differentiated cells (CMs and ECs). The height of the cluster trees represents the similarity or dissimilarity between different objects and groups. C) The number of differentially methylated cytosines (DMCs) identified through pairwise comparisons is depicted in this section. The identified DMCs have a statistical significance (*q* < 0.01) and a methylation difference of at least 25%. D) Another unsupervised hierarchical clustering analysis is conducted, but this time for differentially methylated regions (DMRs) in CpG islands (CGIs) across the genome in PSCs, PSC-CMs, and PSC-ECs. The DMRs shown here have a statistical significance (*q* < 0.01) and amount to a total of 3,452. E) Lastly, an unsupervised hierarchical clustering analysis is presented for 2,324 DMRs located in CGI shores (regions adjacent to CGIs) in PSCs, PSC-CMs, and PSC-ECs. These DMRs also have a statistical significance (*q* < 0.01). **5** The identification of consistent differentially methylated regions (DMRs) in undifferentiated pluripotent stem cells (PSCs) and fully differentiated cells. In panel A, 42 consistent DMRs within CpG islands (CGIs) were found in both PSCs and differentiated cells. Panel B shows 40 consistent DMRs located in CGI shores, which were either hypermethylated or hypomethylated in in vitro fertilization (IVF) samples. Panel C provides the numbers of IVF-specific hypermethylated, IVF-specific hypomethylated, and inter-individual DMRs persistently present in PSCs, PSC-derived cardiomyocytes (PSC-CMs), and PSC-derived endothelial cells (PSC-ECs). The consistent DMRs specific to iPSCs were not found in CGI shores. Panels D and E represent IVF-specific consistent CGI-DMRs identified in undifferentiated PSCs and differentiated cells. Panels F and G demonstrate that the methylation levels of iPSC-specific consistent CGI-DMRs in iPSCs were higher compared to those in non-transgenic embryonic stem cells (nt-ESCs) and IVF-ESCs. Panel H shows the results of Spearman's correlation analysis, indicating a significant correlation between consistent promoter DMRs and the mRNA abundance of the associated genes (*P* < 2.2e − 16). **6** The results of a study examining the toxic effects of doxorubicin on cardiomyocytes (CMs) derived from iPSCs, non-transgenic embryonic stem cells (nt-ESCs), and in vitro fertilization-derived ESCs (IVF-ESCs). Panel (A) shows the dose-dependent impact of doxorubicin on the viability of PSC-CMs. The viability was measured using a Prestoblue cell viability assay, and the results indicate that as the dose of doxorubicin increases, the viability of PSC-CMs decreases. The values were normalized to the viability at 0 μM doxorubicin. Panel (B) displays the effect of doxorubicin treatment on the production of ATP in PSC-CMs. ATP production was measured using a CellTiter-Glo assay, and the data suggests that doxorubicin treatment negatively affects ATP production in PSC-CMs. Panel (C) demonstrates the assessment of cellular apoptosis in PSC-CMs after doxorubicin treatment. A luminescent Caspase 3/7 assay was used to measure apoptosis, and the results indicate that doxorubicin treatment leads to increased cellular apoptosis in PSC-CMs. Panel (D) reveals that the viability of PSC-CMs is not significantly affected after 24 h of doxorubicin treatment. Panel (E) presents the detection of whole-cell reactive oxygen species (ROS), specifically hydrogen peroxide (H2O2), in PSC-CMs after different doses of doxorubicin treatment for 24 h. The data suggests that doxorubicin administration leads to an increase in ROS levels in PSC-CMs. Panel (F) shows the acute influence of doxorubicin treatment on the mitochondrial glutathione (GSH) concentration in PSC-CMs. The GSH concentration was measured using a GSH-Glo Glutathione kit, and the results indicate that doxorubicin treatment has an impact on the mitochondrial GSH concentration in PSC-CMs. Reprinted from [547] with permission from the PNAS

Tumorigenesis is a complex series of events where changes occur in how genes are expressed, how proteins function, and how cells behave, all culminating in the formation of cancerous tumors [536]. While there have been notable advancements in the field of cancer research, several facets of how tumors develop are still not fully comprehended. This lack of understanding presents difficulties in crafting successful treatments for various cancer forms. iPSCs, on the other hand, originate from mature somatic cells that have undergone reprogramming to attain a pluripotent state, enabling them to transform into any cell type within the body [539]. The capability to create iPSCs from individuals afflicted with diverse forms of cancer offers scientists a potent means to investigate the molecular alterations that transpire during the development of tumors. iPSCs can be transformed into various cell categories pertinent to cancer, including cancer stem cells, which are thought to play a pivotal role in the onset and advancement of numerous cancer types [534, 535]. Analyzing the actions of cancer stem cells that originate from iPSCs allows scientists to uncover the fundamental processes driving the onset and advancement of cancer. Beyond serving as a distinct method for investigating how tumors form, iPSCs can serve as valuable tools for screening potential drugs and tailoring medical treatments. iPSCs can be generated from individuals with various cancer types, facilitating the creation of customized cancer models specific to each patient [536]. These models can be used to screen potential therapies, identify drugs that are most effective in specific patient populations, and develop personalized treatment plans. This approach has the potential to significantly improve cancer treatment outcomes by tailoring therapies to individual patients based on their unique genetic makeup and cancer characteristics [532]. Another potential application of iPSCs in cancer research is the development of immunotherapies. Immunotherapies are a promising approach to cancer treatment that involves harnessing the patient’s immune system to fight cancer [539]. Nonetheless, the difficulty in creating successful immunotherapies arises from the intricate interplay between cancer cells and the immune system. iPSCs offer a potential solution by allowing for the creation of patient-specific immune cells, facilitating the development of customized immunotherapeutic approaches [536]. These therapies have the potential to be more effective and have fewer side effects than current approaches to cancer treatment. Despite the potential of iPSCs in cancer research, there are still many challenges that must be addressed [532]. One of the main challenges is the tumorigenic properties of iPSCs. iPSCs have been shown to have a higher propensity to form tumors than other types of stem cells, which could limit their use in cancer research and therapy [536]. However, recent advances in iPSC technology have led to the development of safer and more efficient methods for generating iPSCs, which could help to address this challenge. Another challenge is the ethical and legal considerations surrounding iPSC research [532]. The use of human embryonic stem cells in research has been controversial due to ethical concerns, but iPSCs offer a viable alternative that avoids these issues. However, there are still ethical and legal considerations that must be addressed, such as ensuring that iPSC research is conducted in an ethical and responsible manner and that patient privacy is protected [536]. In a recent investigations provided the study delved into the molecular and functional similarities between differentiated cells originating from induced pluripotent stem cells (iPSCs) and embryonic stem cells derived through somatic cell nuclear transfer (SCNT), known as nt-ESCs [544]. The research compared the differentiation processes and traits of cardiomyocytes (PSC-CMs) and endothelial cells (PSC-ECs) derived from genetically matched sets of iPSCs, nt-ESCs, and in vitro fertilization embryo-derived ESCs (IVF-ESCs). The study revealed that iPSC-derived cells displayed comparable lineage gene expression, cellular diversity, physiological characteristics, and metabolic functions when compared to their corresponding nt-ESC counterparts. Figure 10 illustrates the cardiac differentiation process across various human stem cell types, emphasizing the role of iPSCs. Additionally, Fig. 10 demonstrates the generation of endothelial cells from different pluripotent stem cell sources. The RNA-seq analysis results in Fig. 10 offered insights into the global gene expression profiles of PSCs, PSC-CMs, and PSC-ECs. Furthermore, Fig. 10 provides a comprehensive examination of DNA methylation patterns in these cell types using RRBS-seq. These findings suggest that iPSCs can effectively replace nt-ESCs in generating patient-specific differentiated cells, facilitating disease modeling and preclinical drug testing. Figure 10 identifies consistent differentially methylated regions in undifferentiated PSCs and fully differentiated cells, while Fig. 10 illustrates the impact of doxorubicin-induced toxicity on CMs derived from various sources, including iPSCs, nt-ESCs, and IVF-ESCs. These results underscore the potential of iPSCs in regenerative medicine and emphasize the significance of considering genetic composition when assessing the molecular and functional attributes of differentiated cells. The iPSCs offer significant potential for studying tumorigenesis, developing new cancer therapies, and improving patient outcomes [545, 546]. The ability to generate iPSCs from patients with different types of cancer provides a powerful tool for studying the molecular changes that occur during cancer initiation and progression. iPSCs can also be used for drug screening, personalized medicine, and the development of immunotherapies [24]. While there are still challenges that must be addressed, the potential benefits of iPSC research in tumorigenesis are significant and warrant continued investment in this field [543]. Moreover, iPSCs hold promise for early detection of cancer. Detecting cancer at an early stage is crucial for improving patient outcomes, as it allows for timely intervention and treatment. iPSCs can be utilized to create disease models that mimic the early stages of cancer development, providing valuable insights into the molecular and cellular changes that occur during this critical phase [545, 546]. By studying these models, researchers can identify biomarkers and develop innovative diagnostic tools for the early detection of cancer. This can lead to more effective screening strategies and the ability to detect cancer before it progresses to an advanced stage [543]. Furthermore, iPSCs offer a valuable platform for understanding the role of epigenetic modifications in tumorigenesis. Epigenetic alterations, such as DNA methylation and histone modifications, play a significant role in the development and progression of cancer. iPSCs can be reprogrammed from patient-derived somatic cells, capturing the epigenetic marks present in the original cells [545, 546]. By comparing iPSCs derived from healthy individuals and those with cancer, researchers can identify specific epigenetic modifications associated with tumorigenesis. This knowledge can lead to the development of targeted therapies aimed at reversing or inhibiting these cancer-associated epigenetic changes [24]. Additionally, iPSCs provide an opportunity for studying the tumor microenvironment and its influence on tumorigenesis. The tumor microenvironment consists of various cell types, including immune cells, fibroblasts, and blood vessels, which interact with cancer cells and impact tumor growth and metastasis. iPSCs can be differentiated into these different cell types, allowing researchers to recreate a simplified version of the tumor microenvironment in the laboratory [543]. By studying the interactions between iPSC-derived tumor cells and the surrounding microenvironment, researchers can gain insights into the complex signaling pathways and cellular crosstalk involved in cancer progression. This knowledge can aid in the development of novel therapeutic strategies targeting the tumor microenvironment [545, 546].

### Comparison of iPSCs with traditional cancer cell models

The iPSCs have emerged as a promising tool in cancer research, providing unique advantages compared to traditional cancer cell models [548]. Comparing iPSCs to traditional cancer cell models highlights the unique advantages and limitations of each model. While traditional cancer cell models have been critical in advancing our understanding of cancer biology and drug discovery [545, 546], iPSCs offer novel capabilities that enhance our ability to study cancer development and progress towards personalized medicine [24]. By leveraging the strengths of both models, researchers can continue to make progress in cancer research, ultimately leading to more effective therapies and improved patient outcomes. While traditional cancer cell models, such as established cancer cell lines and patient-derived xenografts (PDX), have been instrumental in advancing our understanding of cancer biology and drug discovery, iPSCs offer distinct benefits that enhance our ability to study tumorigenesis and develop effective therapies [24, 549]. One key advantage of iPSCs is their ability to recapitulate the genetic and epigenetic diversity observed in tumors [550]. Traditional cancer cell models often represent a specific subtype or clone of cancer cells, limiting their applicability to studying the heterogeneity seen in clinical tumors [546]. In contrast, iPSCs can be generated from various somatic cells, including cancer cells, allowing for the generation of iPSC lines that reflect the genomic and epigenomic alterations found in individual patients [551, 552]. This enables researchers to study the effects of specific mutations and alterations on cancer development and progression, offering valuable insights into personalized medicine approaches [552]. Moreover, iPSCs have the unique ability to differentiate into various cell types, including different lineages of cancer cells. This feature allows researchers to generate diverse populations of cancer cells that mirror the heterogeneity within tumors [553]. By differentiating iPSCs into specific cancer cell lineages, researchers can investigate the molecular changes associated with cancer progression and metastasis as well as identifying potential therapeutic targets [554]. In addition to their capacity for differentiation, iPSCs can also be utilized to model early stages of cancer development [125]. Traditional cancer cell models often represent advanced stages of the disease, limiting the ability to study the initial events leading to cancer formation [543]. iPSCs, on the other hand, can be reprogrammed from somatic cells obtained from healthy individuals, allowing researchers to study the early stages of carcinogenesis [554]. This provides a unique opportunity to uncover the molecular changes and cellular processes that drive cancer initiation, offering insights into early detection and prevention strategies [555]. Furthermore, iPSCs offer a powerful tool for drug screening and personalized therapy development. Traditional cancer cell models have been extensively used in high-throughput drug screening assays. However, these models often fail to accurately predict drug responses in patients, partly due to their limited representation of patient-specific genetic backgrounds [556]. iPSCs, with their ability to capture patient-specific genetic information, can be utilized to generate personalized cancer cell models. By deriving iPSCs from patient samples and differentiating them into cancer cells, researchers can create an individualized platform for testing drug efficacy and toxicity, aiding in the development of personalized treatment regimens [548]. Despite these advantages, there are challenges and limitations associated with iPSCs compared to traditional cancer cell models. One major challenge is the complexity and cost of iPSC generation and maintenance [33]. The reprogramming process itself is time-consuming and requires specialized expertise. Additionally, the long-term culture of iPSCs can lead to genomic instability and alterations in their epigenetic landscape, potentially affecting their suitability as cancer models [542]. These technical challenges need to be addressed to ensure the reliability and reproducibility of iPSC-based cancer research [542]. Moreover, ethical considerations surrounding the use of iPSCs should be taken into account. The generation of iPSCs involves the manipulation of human embryos or the reprogramming of adult somatic cells, raising ethical concerns and legal regulations. These ethical considerations should be carefully addressed to ensure the responsible and ethical use of iPSCs in cancer research and therapy development [557]. In conclusion, iPSCs offer distinct advantages when compared to traditional cancer cell models, enabling researchers to study the complexity and heterogeneity of tumors more effectively [549]. Their ability to recapitulate the genetic and epigenetic diversity of tumors, differentiate into multiple cell lineages, model early stages of carcinogenesis, and generate personalized cancer cell models makes iPSCs a powerful tool in cancer research [125, 540]. However, challenges and limitations such as technical difficulties and ethical considerations need to be addressed to ensure the reliability and ethical use of iPSCs in cancer research and therapy development. Table 7 presents a comprehensive overview of the advantages and limitations associated with two prominent cancer research models: iPSCs and traditional cancer cell models.

**Table 7:** Comprehensive analysis of advantages and limitations of iPSCs and traditional cancer cell models

Type of Cell Model	Advantages	Limitations	Additional Insights	References
iPSCs	Personalized Disease Modeling: Can be generated from patient samples, allowing personalized disease modeling	Complex Differentiation: Generating specific cell types from iPSCs can be challenging and time-consuming	iPSCs offer a unique opportunity for patient-specific research, but their complex differentiation can be a practical hurdle	[543]
	Disease Modeling: iPSCs can be differentiated into various cell types affected by cancer, providing a relevant model for studying disease mechanisms	Genetic Variability: iPSC lines may have genetic variations that can influence experimental outcomes	Researchers need to carefully account for genetic variability when working with iPSCs	[24]
	Drug Screening: iPSCs can be used to test the efficacy and toxicity of potential cancer therapeutics before clinical trials	Tumorigenic Potential: iPSCs can form tumors if not properly differentiated or controlled	Stringent differentiation protocols are crucial to avoid tumorigenicity in iPSC-based models	[24, 558]
Traditional Cancer Cell Models	Established Cell Lines: A wide range of well-characterized cancer cell lines are available, enabling standardized experiments and comparisons	Genetic Drift: Cancer cell lines may undergo genetic changes over time, deviating from the original tumor characteristics	Periodic authentication and monitoring are essential to maintain genetic stability in cell lines	[546]
	Simplified Models: Traditional cancer cell lines can provide a simplified representation of specific cancer types, facilitating experimental manipulations and high-throughput screening	Lack of Tumor Microenvironment: These models often lack the complex interactions with stromal cells and immune components present in actual tumors	Traditional cell lines may not fully recapitulate the tumor microenvironment, limiting their translational relevance	[546]
	Faster Experiments: Traditional cancer cell lines can proliferate rapidly, allowing for faster experimental results and drug screening	Limited Clinical Relevance: These models may not fully recapitulate the heterogeneity and complexity of tumors in patients	Rapid proliferation comes at the cost of potential oversimplification of cancer biology	[545]
	Availability of Large-Scale Datasets: Many traditional cancer cell lines have extensive genomic and proteomic data available, aiding in data analysis and interpretation	Cross-Contamination: Cell lines can get contaminated or misidentified, leading to inaccurate or unreliable results	Data derived from cell lines should be interpreted with caution and validated using other models	[546]
	Easy Manipulation: Traditional cancer cell lines can be easily manipulated genetically, allowing for targeted gene knockouts or overexpression studies	Metabolic Changes: Cell lines may exhibit metabolic alterations compared to primary tumor cells, affecting drug response and metabolism studies	Genetic manipulation offers experimental flexibility, but metabolic differences should be considered when assessing drug responses	[545]

### The use of iPSCs to study the tumorigenic properties of cancer cells

The iPSCs have emerged as a powerful tool in the study of cancer biology. With their ability to differentiate into various cell types and self-renew, iPSCs offer a unique model for investigating the tumorigenic properties of cancer cells [559]. Tumorigenic properties refer to the ability of cancer cells to form tumors. Cancer cells can acquire these properties through a variety of molecular changes, such as alterations in gene expression, mutations, and changes in signaling pathways [560]. By studying these properties, researchers can gain insights into the mechanisms of cancer development and identify potential targets for cancer therapy [561]. One of the challenges in studying cancer is the heterogeneity of tumor cells. Cancer cells are genetically and phenotypically diverse, even within the same tumor [562]. This heterogeneity makes it difficult to accurately characterize the properties of cancer cells and develop effective therapies [562]. iPSCs offer a solution to this challenge by providing a homogeneous population of cells with a known genetic background. iPSCs can be generated from patient-derived somatic cells, including cancer cells. These iPSCs retain the genetic and epigenetic alterations found in the original cells and can be differentiated into various cell types, including cancer cells [552]. By comparing the properties of iPSC-derived cancer cells with those of the original cancer cells, researchers can identify changes in gene expression and signaling pathways that contribute to tumorigenesis. One approach to studying the tumorigenic properties of cancer cells using iPSCs involves the generation of tumor organoids [563]. Tumor organoids are three-dimensional cultures of cells that mimic the structure and function of a tumor [563]. iPSC-derived cancer cells can be used to generate tumor organoids that closely resemble the original tumor [368]. These organoids can be used to study the properties of the tumor, such as growth rate, invasion, and response to therapy [564]. Another approach involves the use of iPSC-derived cancer cells in xenograft models. Xenograft models involve the transplantation of human cancer cells into immunocompromised mice [565]. iPSC-derived cancer cells can be used to generate xenograft models that closely resemble the original tumor [565]. These models can be used to study the properties of the tumor in vivo, such as growth rate, invasion, and response to therapy. The use of iPSCs to study the tumorigenic properties of cancer cells has several potential implications for cancer therapy. By identifying the molecular changes that contribute to tumorigenesis, researchers can develop targeted therapies that selectively inhibit these changes [566]. iPSC-derived cancer cells can also be used to screen drugs for their efficacy in killing cancer cells. This approach has the potential to identify new drugs and drug combinations that are effective in treating cancer [5]. Additionally, iPSC-derived cancer cells can be used in personalized medicine [24]. By generating iPSCs from patient-derived cells and differentiating them into cancer cells, researchers can create a patient-specific model of the tumor. This model can be used to identify the most effective treatment for the patient, based on the properties of their tumor [24]. Despite the potential benefits of using iPSCs to study the tumorigenic properties of cancer cells, there are several limitations and challenges. One challenge is the cost and technical expertise required to generate and maintain iPSCs [567]. Additionally, iPSC-derived cancer cells may not fully capture the complexity of the original tumor, as they may lack the microenvironmental cues and interactions with other cell types that contribute to tumorigenesis [490]. Another limitation is the potential for iPSC-derived cancer cells to form tumors when transplanted into mice [506]. iPSCs have been shown to have tumorigenic potential, and this risk is heightened when iPSCs are differentiated into cancer cells. Therefore, careful consideration and rigorous characterization are necessary when using iPSC-derived cancer cells in xenograft models or transplantation experiments [506]. Despite these challenges, the use of iPSCs to study the tumorigenic properties of cancer cells holds great promise. The ability to generate patient-specific models of tumors allows for personalized approaches to cancer therapy [24]. By understanding the molecular changes that drive tumorigenesis, researchers can develop targeted therapies that are tailored to individual patients, increasing the chances of successful treatment outcomes [24]. Furthermore, the use of iPSC-derived cancer cells in drug screening can accelerate the discovery and development of new cancer therapies [568]. Traditional drug screening methods often fail to accurately predict the response of human tumors due to the limitations of using immortalized cancer cell lines [569]. iPSC-derived cancer cells provide a more representative model that better reflects the complexity and heterogeneity of human tumors, increasing the likelihood of identifying effective treatments [559]. In addition to their potential in drug discovery, iPSCs offer valuable insights into the early detection and prevention of cancer. By studying the properties of iPSC-derived cancer cells, researchers can identify biomarkers and molecular signatures associated with early stages of tumorigenesis. This knowledge can contribute to the development of non-invasive diagnostic tools for the early detection of cancer, enabling timely interventions and improved patient outcomes. The iPSCs have opened up new avenues for studying the tumorigenic properties of cancer cells. Their ability to recapitulate the genetic and epigenetic alterations found in cancer cells, combined with their potential for differentiation into various cell types, provides a valuable platform for investigating the mechanisms of tumorigenesis [570]. The use of iPSC-derived cancer cells in tumor organoids and xenograft models allows for the study of tumor growth, invasion, and response to therapy in a controlled and reproducible manner [571]. Moreover, iPSCs have the potential to drive advancements in personalized medicine, drug discovery, and early cancer detection. However, challenges such as cost, technical expertise, tumor heterogeneity, tumorigenicity, and ethical considerations must be addressed to fully harness the potential of iPSCs in cancer research [507, 572]. Continued investment in iPSC research and collaboration between scientists, clinicians, and ethicists is crucial to unlock the full potential of iPSCs in understanding and treating cancer.

### The use of iPSCs to study the molecular and genetic changes during tumorigenesis

One recent study published in the journal Cell Stem Cell demonstrated the potential of iPSCs in cancer research by using them to study the molecular changes that occur during the progression of colorectal cancer. The researchers generated iPSCs from both healthy individuals and patients with early-stage colorectal cancer and then differentiated them into intestinal organoids. Using this model, they were able to identify key molecular changes that occur during the early stages of colorectal cancer development, including alterations in the Wnt signaling pathway and changes in the expression of genes involved in cellular differentiation. They also observed an increase in the number of cancer stem cells present in the organoids derived from cancer patients, which could contribute to the development and progression of the disease [573]. Another study published in the journal Nature Communications utilized iPSCs to study the genetic changes that occur during the development of lung cancer. The researchers used iPSCs derived from patients with lung cancer to create lung organoids that closely resemble the cellular architecture of human lung tissue. By comparing the genetic profiles of the lung organoids derived from healthy individuals and patients with lung cancer, the researchers were able to identify mutations in several key genes that are frequently mutated in lung cancer, including TP53, KRAS, and EGFR. They also observed changes in the expression of genes involved in cell adhesion and signaling pathways that are known to play a role in cancer development [574]. These studies demonstrate the potential of iPSCs in cancer research, particularly in understanding the early molecular and genetic changes that occur during tumorigenesis. iPSC-derived models can provide a more accurate representation of human disease than traditional cell culture models and offer a more cost-effective and ethical alternative to animal models [543]. Additionally, iPSCs have the potential to be used in the development of personalized cancer therapies. By generating iPSCs from individual patients, researchers can create organoids that closely resemble the patient’s own tissue, allowing for more accurate testing of potential treatments and the development of personalized therapeutic approaches [575–577]. However, there are also challenges associated with the use of iPSCs in cancer research. One major limitation is the potential for iPSCs to harbor genetic abnormalities or epigenetic changes that could affect their behavior and skew the results of experiments. Researchers must carefully screen and characterize iPSCs to ensure that they are of high quality and free from abnormalities before using them in experiments [542]. Furthermore, there are ethical and legal considerations associated with the use of iPSCs, particularly when it comes to the creation and use of iPSCs derived from human embryos [557]. Researchers must navigate complex regulatory frameworks and ensure that they are conducting their research in an ethical and responsible manner [557]. Despite these challenges, the use of iPSCs in cancer research holds tremendous promise for advancing our understanding of tumorigenesis, identifying new therapeutic targets, and developing personalized treatments for the disease. With continued investment and research, iPSCs have the potential to revolutionize cancer research and improve outcomes for patients around the world [574].

## Applications of iPSCs in tumorigenesis

The iPSCs have emerged as a powerful tool in cancer research, offering diverse applications in various aspects of tumorigenesis [507]. The utilization of patient-derived iPSCs has undeniably emerged as a powerful tool in the realm of cancer research. Various research groups across the globe have undertaken compelling investigations into the intricate relationship between germline mutations and the occurrence of cancer. Through meticulous experimentation, these dedicated teams have harnessed the potential of iPSCs to unveil profound insights into the underlying mechanisms of cancer development and, in turn, drive forward the frontiers of drug discovery [572]. One notable aspect of this vibrant field is the considerable body of work that has culminated in critical findings regarding cancer etiology and progression. The omission of a comprehensive review of these pivotal discoveries by the authors is a regrettable oversight. These findings represent a cornerstone of contemporary iPSC-based cancer research, providing valuable benchmarks for the broader scientific community. By neglecting to acknowledge and incorporate these contributions, the authors risk presenting an incomplete analysis of the field, leaving gaps in the understanding of the complex interplay between germline mutations and cancer [574]. To ensure a well-rounded and robust assessment of the iPSC-based cancer research landscape, it is imperative for the authors to address this significant deficiency in their work. By integrating these important contributions into their analysis, the authors can enrich their study and provide a more holistic perspective on the transformative potential of patient-derived iPSCs in deciphering the mysteries of cancer. This collaborative and inclusive approach will not only benefit the authors’ work but also foster a deeper understanding of the broader scientific community’s collective efforts in unraveling the complexities of cancer biology [542]. Moreover, the incorporation of these crucial findings from various research groups will enhance the overall scientific rigor of the authors’ analysis. By acknowledging the extensive body of work conducted in the field of iPSC-based cancer research, the authors can strengthen the foundation upon which their research is built. This inclusivity will not only validate the efforts of their peers but also elevate the credibility and comprehensiveness of their own work [574]. Additionally, the integration of these research findings can provide a broader context for the authors’ work, allowing readers and fellow researchers to appreciate the interconnectedness of different studies within this multidisciplinary field. It can also help identify potential gaps or areas that require further investigation, fostering a collaborative environment for future research endeavors [575–577].

### The use of iPSCs in developing new cancer therapies

The iPSCs have emerged as a promising tool in the development of new cancer therapies, offering unprecedented opportunities to advance our understanding of tumorigenesis and transform the landscape of cancer treatment [578, 579]. These remarkable cells, which are derived from adult somatic cells through a process of reprogramming, possess the ability to differentiate into various cell types, including those relevant to cancer, making them an invaluable resource for studying disease mechanisms and developing novel therapeutic strategies [24, 580, 581]. Table 8 presents a comprehensive overview of the current iPSC-based cancer therapies that have emerged as promising strategies in the field of oncology. One of the key advantages of iPSCs in cancer therapy lies in their ability to provide a robust and patient-specific model for studying tumorigenesis [564, 577]. By reprogramming cells from cancer patients, researchers can generate iPSCs that carry the genetic and epigenetic signatures of the individual’s tumor [550]. These iPSCs can then be differentiated into the specific cell types affected by cancer, allowing for detailed investigation of the molecular changes and abnormalities associated with the disease [582]. This personalized approach enables researchers to gain insights into the underlying mechanisms of cancer development, identify novel therapeutic targets, and tailor treatment strategies to individual patients [24, 581, 583]. Furthermore, iPSCs offer a unique platform for drug screening and the development of targeted therapies [540, 584]. Traditional cancer cell lines and animal models often fail to accurately replicate the complexity and heterogeneity of human tumors, limiting their predictive value in preclinical studies [545, 546]. iPSC-derived cancer cells, on the other hand, can more faithfully recapitulate the genetic and phenotypic characteristics of the patient’s tumor, making them an ideal tool for testing the efficacy and toxicity of potential therapeutics [576, 585]. Through high-throughput screening approaches, large libraries of compounds can be screened against iPSC-derived cancer cells, leading to the identification of novel drug candidates and personalized treatment options [548, 586]. Immunotherapies, which harness the body’s immune system to target and eliminate cancer cells, have revolutionized cancer treatment in recent years [555, 587]. iPSCs hold great promise in this field as well, as they can be engineered to express tumor-specific antigens and used as a source for generating patient-specific immune cells. By differentiating iPSCs into immune cells, such as T cells or natural killer cells, researchers can create a personalized immunotherapy approach that is tailored to the patient’s specific tumor antigens [588–590]. This personalized immunotherapy has the potential to enhance the efficacy of treatment while minimizing off-target effects, leading to more targeted and effective cancer therapies [104, 531, 581, 591]. Moreover, iPSCs can be utilized for cancer early detection and diagnosis. Through their ability to differentiate into various cell types, including those found in tumors, iPSCs can be employed to generate specific cell populations that mimic the early stages of cancer development [533, 539, 592]. By studying the molecular changes and aberrant signaling pathways present in these iPSC-derived cancer cells, researchers can gain valuable insights into the early detection and diagnosis of cancer [267, 554, 593]. This knowledge can then be translated into the development of innovative diagnostic tools and biomarkers for improved cancer screening and early intervention [125]. Despite the tremendous potential of iPSCs in cancer therapy, several challenges and limitations need to be addressed. One major hurdle is the risk of tumorigenicity associated with the transplantation of iPSCs or their derivatives [594, 595]. The pluripotent nature of iPSCs renders them capable of uncontrolled growth and potential tumor formation. Therefore, stringent quality control measures and extensive characterization of iPSCs and their differentiated progeny are essential to ensure their safety and efficacy in clinical applications [542, 594].

**Table 8:** Current iPSC-based cancer therapies

Therapy Name	Target Indication	Mechanism	Key Point	References
iPSC-Derived NK Cells	Solid Tumors	Natural Killer (NK) cells derived from iPSCs are used to target and kill cancer cells	Promising immunotherapy approach with enhanced tumor-specific killing abilities	[596]
iPSC-Derived T Cells	Leukemia, Lymphomas	T cells derived from iPSCs are genetically modified to express chimeric antigen receptors (CARs) to specifically recognize and eliminate cancer cells	Offers a personalized and targeted therapy for hematological malignancies	[551]
iPSC-Derived Dendritic Cells	Melanoma, Lung Cancer	Dendritic cells derived from iPSCs are used to stimulate the immune system and activate T cells against cancer cells	Shows potential as an adjuvant therapy to boost the immune response against solid tumors	[590]
iPSC-Derived Mesenchymal Stem Cells	Various Cancers	Mesenchymal stem cells derived from iPSCs are engineered to deliver anti-cancer agents or suppress tumor growth through paracrine signaling	Provides a versatile platform for targeted drug delivery and modulating the tumor microenvironment	[597]
iPSC-Based Gene Therapy	Various Cancers	iPSCs are genetically modified to correct cancer-related genetic mutations and then differentiated into specific cell types for transplantation	Offers a potential curative approach by addressing the underlying genetic defects in cancer cells	[555]
iPSC-Derived CAR-T Cells	Solid Tumors	T cells derived from iPSCs are engineered to express chimeric antigen receptors (CARs) targeting specific cancer antigens for enhanced tumor recognition and elimination	Shows potential for treating solid tumors that are challenging to target with conventional CAR-T cell therapies	[588, 598]
iPSC-Derived Natural Killer (NK) Cell Engagers	Hematological Malignancies	iPSC-derived NK cells are modified to express bispecific or trispecific antibodies that bind both NK cells and cancer cells, enhancing the killing of cancer cells	Offers a novel approach to improve NK cell-mediated cytotoxicity and potentially overcome tumor immune evasion mechanisms	[599]
iPSC-Derived Oncolytic Viruses	Various Cancers	iPSCs are genetically modified to produce oncolytic viruses that selectively replicate in and kill cancer cells while sparing healthy cells	Represents a promising strategy for targeted cancer cell destruction with potential for systemic administration	[537, 600]
iPSC-Derived Tumor Organoids	Personalized Medicine	iPSCs are differentiated into 3D organoids that mimic the patient's tumor, allowing for drug screening, personalized treatment selection, and studying tumor biology	Enables precision medicine by providing a patient-specific model for drug testing and studying tumor characteristics	[125, 130, 601]
iPSC-Based Cancer Vaccines	Preventive Therapy	iPSCs are engineered to express tumor-specific antigens, and the resulting antigen-presenting cells stimulate the immune system to recognize and attack cancer cells	Offers a potential strategy for cancer prevention and immune priming against future cancer development	[109, 570]
iPSC-Derived Tumor-Targeting Nanoparticles	Various Cancers	iPSCs are utilized to generate tumor-targeting nanoparticles loaded with therapeutic agents, allowing for precise drug delivery to cancer cells	Offers a targeted approach for delivering chemotherapy drugs and reducing systemic toxicity	[218]
iPSC-Derived Tumor-Specific Viral Vectors	Various Cancers	iPSCs are engineered to produce viral vectors carrying tumor-specific antigens or therapeutic genes, which can selectively infect and destroy cancer cells	Shows potential for precise and efficient cancer cell targeting while minimizing off-target effects	[536]
iPSC-Based Immunotherapies	Various Cancers	iPSCs are differentiated into various immune cell types, such as macrophages or dendritic cells, to stimulate anti-tumor immune responses or modulate the tumor microenvironment	Offers a versatile platform for developing different immunotherapeutic approaches tailored to specific cancer types and patient needs	[104, 602]
iPSC-Derived Microenvironment Models	Drug Development	iPSCs are differentiated into multiple cell types to recreate tumor microenvironments in vitro, allowing for studying cancer biology, drug response, and developing personalized therapies	Facilitates the development of more effective and targeted cancer treatments through improved understanding of tumor biology and drug testing	[603]
iPSC-Derived Exosomes	Various Cancers	iPSCs are used to generate exosomes loaded with therapeutic molecules such as RNA, proteins, or small molecules, which can be delivered to cancer cells to modulate their behavior or sensitize them to treatments	Provides a novel approach for targeted delivery of therapeutic cargo and potential modulation of cancer cell behavior	[604]
iPSC-Derived Tumor-Associated Macrophages	Solid Tumors	iPSCs are differentiated into macrophages with tumor-specific characteristics, enabling the study of tumor-immune interactions and potential modulation of the tumor microenvironment	Offers insights into tumor-immune dynamics and potential therapeutic interventions targeting tumor-associated macrophages	[605]
Tumor-Specific Antigen iPSC-Derived Libraries	Immunotherapy	iPSCs are used to generate libraries of tumor-specific antigens, which can be utilized for personalized cancer immunotherapies to trigger immune responses against specific cancer cells	Enables the development of customized immunotherapies targeting individual patients' unique tumor antigens	[324, 531]
iPSC-Derived Gene Editing for Tumor Suppression	Various Cancers	iPSCs are genetically modified to correct cancer-associated gene mutations or introduce tumor suppressor genes, which can be used for cell replacement therapies or as a platform for studying cancer genetics	Provides a potential strategy for directly targeting cancer-causing genetic mutations and exploring the role of specific genes in cancer development	[555, 606]
iPSC-Based Drug Screening Platforms	Drug Development	iPSCs are differentiated into specific cancer cell types and utilized as high-throughput platforms for testing the efficacy and toxicity of potential anti-cancer drugs, aiding in drug discovery and development	Enhances the drug development process by enabling rapid and cost-effective screening of potential therapeutic compounds	[542, 584]
iPSC-Derived Oncolytic Vaccines	Various Cancers	iPSCs are engineered to express tumor-specific antigens and viral components, leading to the production of oncolytic vaccines that stimulate anti-cancer immune responses	Provides a dual mechanism of viral-mediated tumor cell destruction and immune system activation	[537]
iPSC-Derived Antibody Therapies	Solid Tumors, Leukemia	iPSCs are used to generate antibodies targeting specific cancer cell surface markers, which can be utilized for antibody-based immunotherapies or as diagnostic tools	Offers a potential targeted therapy option with reduced off-target effects and improved specificity	[581, 583]
iPSC-Based Tumor Invasion Models	Cancer Research	iPSCs are differentiated into organoids or 3D models that mimic the invasive behavior of cancer cells, enabling the study of tumor invasion mechanisms and testing of anti-metastatic therapies	Offers a valuable tool for investigating the complex process of tumor invasion and developing interventions to prevent metastasis	[530, 556]
iPSC-Derived Cancer Stem Cell Targeting	Solid Tumors	iPSCs are engineered to express molecules or antibodies that specifically target and eliminate cancer stem cells, which are implicated in tumor initiation, progression, and therapy resistance	Offers a potential strategy for eradicating cancer-initiating cells and preventing tumor recurrence	[542]
iPSC-Derived Immune Checkpoint Modulators	Various Cancers	iPSCs are differentiated into immune cells or engineered to express molecules that can modulate immune checkpoint pathways, enhancing anti-tumor immune responses	Provides a means to overcome immune suppression and improve the effectiveness of cancer immunotherapy	[589, 607, 608]
iPSC-Based Drug Sensitivity Testing	Precision Medicine	iPSCs derived from patient samples are differentiated into cancer cells and used to test the response to various drugs, guiding personalized treatment selection based on individual drug sensitivity profiles	Facilitates personalized medicine by predicting patient-specific drug responses and optimizing treatment strategies	[584, 609]
iPSC-Based Tumor Immunogenicity Studies	Cancer Immunology	iPSCs are utilized to generate tumor cells with specific genetic alterations or antigen profiles, allowing for the investigation of tumor immunogenicity and the development of personalized immunotherapeutic strategies	Enables a better understanding of the immune response against cancer cells and the design of tailored immunotherapies	[610]

### The use of iPSCs in drug screening and development

The use of iPSCs in drug screening and development has emerged as a promising avenue in the field of medicine. iPSCs, which are derived from adult cells reprogrammed to possess pluripotency, offer a unique platform for studying disease mechanisms and evaluating the efficacy and safety of potential drugs [568, 575, 584]. This paragraph will delve into the advantages of iPSCs in drug screening and development, highlighting their potential to revolutionize the process of discovering new therapies. One of the key advantages of using iPSCs in drug screening is their ability to recapitulate disease phenotypes in a controlled laboratory setting [575]. By reprogramming patient-specific cells, researchers can generate iPSCs that carry the genetic signature of a particular disease [553, 611]. These disease-specific iPSCs can then be differentiated into relevant cell types affected by the disease, such as neurons for neurodegenerative disorders or cardiomyocytes for cardiovascular diseases [611, 612]. This approach enables scientists to model the disease in a dish, allowing for the identification of novel drug targets and the screening of potential therapeutics in a more physiologically relevant context [576, 613]. Furthermore, iPSCs provide a valuable tool for studying rare diseases or conditions where access to patient samples is limited. These cells can be generated from easily accessible somatic cells, such as skin cells or blood cells, and then differentiated into the desired cell types [21]. This not only circumvents the need for invasive procedures to obtain patient-specific samples but also allows for the creation of large, diverse libraries of disease-specific iPSCs [556, 576, 614]. These libraries can be shared among researchers, fostering collaboration and accelerating drug discovery efforts for conditions that have previously been challenging to study. The use of iPSCs in drug screening also holds promise for personalized medicine. iPSCs can be derived from individual patients, allowing for the development of tailored therapies based on a patient’s specific genetic background. This approach has the potential to transform the field of oncology, as it can aid in the identification of personalized treatment strategies for cancer patients. By generating iPSCs from tumor cells, researchers can create a personalized model that mimics the unique characteristics of a patient’s cancer [15, 615, 616]. This model can be used to screen a variety of drugs and identify the most effective treatment options, thereby improving patient outcomes and reducing unnecessary exposure to ineffective therapies [616]. In addition to personalized medicine, iPSCs offer an invaluable resource for drug toxicity testing [617–619]. Many promising drug candidates fail during the later stages of development due to unexpected toxic effects on vital organs or systems. By using iPSCs to generate different cell types, researchers can evaluate the potential toxicity of drugs in a controlled and reproducible manner [619]. For instance, iPSC-derived liver cells can be used to assess the hepatotoxicity of drug candidates [620], while iPSC-derived cardiomyocytes can provide insights into potential cardiac side effects [621, 622]. This early identification of drug toxicity can help pharmaceutical companies make informed decisions about which compounds to advance in the drug development pipeline, ultimately reducing the risk of adverse effects in clinical trials and improving patient safety [622]. Moreover, iPSCs enable the screening of existing drugs for new therapeutic applications [623]. Repurposing known drugs for different diseases can significantly reduce the time and cost associated with developing new treatments. iPSCs provide a reliable platform for evaluating the efficacy of approved drugs in various disease models. By exposing iPSC-derived disease-relevant cells to a library of known compounds, researchers can identify drugs that exhibit unexpected therapeutic effects or synergistic interactions with existing therapies [624]. This approach has the potential to uncover new treatment options for a range of diseases, opening up avenues for drug repurposing and expanding the therapeutic arsenal available to clinicians. The use of iPSCs in drug screening and development holds tremendous potential to transform the field of medicine [568, 611]. These versatile cells allow for the generation of disease-specific models, personalized medicine approaches, toxicity testing, and drug repurposing efforts. iPSCs provide a unique platform for studying disease mechanisms and evaluating the efficacy and safety of potential drugs [535, 623]. By reprogramming patient-specific cells, researchers can create disease-specific iPSCs that accurately recapitulate the genetic signature and phenotypic characteristics of various diseases [594, 625, 626]. The ability to generate disease-specific cell types from iPSCs allows researchers to study the underlying mechanisms of diseases in a controlled laboratory environment [622]. This approach provides valuable insights into disease progression, identifying key molecular pathways and targets that can be exploited for therapeutic interventions [609, 614]. By screening potential drugs on iPSC-derived disease models, researchers can assess their effectiveness in restoring normal cellular function and halting disease progression [627]. This screening process helps prioritize drug candidates for further development, increasing the efficiency of the drug discovery process and reducing the reliance on animal models. Personalized medicine is another area where iPSCs have the potential to revolutionize drug development. By generating iPSCs from individual patients, it is possible to create patient-specific models that accurately reflect their genetic makeup and disease characteristics. This personalized approach allows for tailored treatment strategies, optimizing therapeutic outcomes and minimizing adverse effects [241, 617]. iPSCs can be used to screen a variety of drugs on patient-specific disease models, identifying the most effective treatments for individual patients. This approach has particular relevance in the field of oncology, where tumor-derived iPSCs can be utilized to screen a range of targeted therapies and chemotherapeutic agents, enabling clinicians to make informed treatment decisions based on the unique biology of a patient’s tumor [15]. One of the critical advantages of iPSCs in drug screening is their potential to predict drug toxicity and side effects. Adverse drug reactions and toxic effects are significant challenges in drug development. iPSCs offer a reliable and scalable platform for assessing drug toxicity by differentiating them into organ-specific cell types [585, 617, 618]. By subjecting iPSC-derived cells to potential drug candidates, researchers can evaluate their safety profiles, identifying any potential toxic effects on specific organs or systems [620]. With ongoing research and advancements in iPSC technology, the integration of iPSCs in drug discovery pipelines holds great promise for accelerating the development of safe and effective treatments for a wide range of diseases.

### The use of iPSCs in personalized medicine

The use of iPSCs in personalized medicine has emerged as a groundbreaking approach with the potential to revolutionize patient care [568, 575, 584]. Personalized medicine aims to tailor medical treatments to individual patients based on their unique genetic, epigenetic, and environmental characteristics. iPSCs, which can be generated from adult cells through reprogramming, offer a remarkable tool for modeling diseases, understanding individual variations, and developing personalized therapies [575]. One of the primary advantages of iPSCs in personalized medicine is their ability to recapitulate the genetic makeup of individual patients [623]. By reprogramming cells from patients with specific diseases or conditions, iPSCs can serve as disease models that accurately reflect the genetic variations present in those patients. This allows researchers to study the underlying mechanisms of diseases at the cellular level and design personalized treatment strategies [622]. Furthermore, iPSCs can be differentiated into various cell types, including those affected by specific diseases. This differentiation potential enables the generation of disease-specific cells, such as cardiomyocytes for heart diseases or neurons for neurodegenerative disorders [568, 575, 584]. By studying these disease-specific cells derived from iPSCs, researchers can gain insights into the molecular changes associated with the disease and identify potential therapeutic targets. In personalized medicine, iPSCs have shown great promise in drug discovery and development [622]. Traditional drug development often relies on animal models or cell lines that may not fully capture the complexity of human diseases. iPSCs, on the other hand, provide a human-specific platform for drug screening and testing. By generating iPSCs from patients with varying responses to specific drugs, researchers can evaluate drug efficacy and toxicity on patient-specific cells [575]. This approach has the potential to optimize drug selection and dosage, leading to improved treatment outcomes and reduced adverse effects. Moreover, iPSCs can be utilized in the field of regenerative medicine, which aims to replace or repair damaged tissues and organs [623]. By differentiating iPSCs into specific cell types, it becomes possible to generate patient-specific cells for transplantation. This approach mitigates the risk of immune rejection, as the transplanted cells are derived from the patient’s own iPSCs. For instance, iPSCs can be differentiated into cardiomyocytes and used for cell-based therapy in patients with heart diseases. This personalized regenerative approach holds immense potential for restoring tissue function and improving patient outcomes [568, 575, 584]. The application of iPSCs in cancer personalized medicine is particularly exciting. Cancer is a highly heterogeneous disease, and treatment response can vary significantly among patients. iPSCs offer the opportunity to generate patient-specific cancer models that closely resemble the individual’s tumor. These models can be used to study the molecular characteristics of the cancer, identify potential therapeutic targets, and predict the response to specific treatments. iPSC-based cancer models also facilitate the screening of anti-cancer drugs, enabling the selection of personalized treatment regimens based on the unique genetic profile of each patient's cancer cells [622]. However, despite the immense potential of iPSCs in personalized medicine, several challenges need to be addressed. The process of reprogramming cells into iPSCs is complex and time-consuming, limiting their immediate clinical application. Additionally, ensuring the safety and efficacy of iPSC-derived cells for transplantation requires rigorous quality control and validation [575]. Moreover, ethical considerations surrounding the generation and use of iPSCs, such as obtaining informed consent and protecting patient privacy, must be carefully addressed to ensure responsible and ethical practices. The iPSCs hold tremendous promise in personalized medicine, offering a unique platform for disease modeling, drug screening, and regenerative therapies [568, 575, 584]. Their ability to recapitulate patient-specific genetic variations and differentiate into disease-specific cells provides valuable insights into individual diseases and facilitates the development of personalized treatment strategies. While challenges exist, continued research and development in iPSC technology, along with the establishment of robust ethical guidelines, will pave the way for the widespread implementation of iPSCs in personalized medicine [623]. To unlock the full potential of iPSCs in personalized medicine, further research is needed to optimize the reprogramming process and improve the efficiency of iPSC generation. Advances in gene-editing technologies, such as CRISPR-Cas9, have the potential to enhance the precision and safety of iPSC reprogramming, making it a more feasible and reliable method for clinical applications [575]. Additionally, efforts should be made to develop standardized protocols for iPSC differentiation into various cell types, ensuring consistency and reproducibility across different laboratories and research settings [622]. Another crucial aspect that requires attention is the establishment of robust bioinformatics and data analysis pipelines to handle the large amount of data generated from iPSC-based personalized medicine studies. Integration of genomics, transcriptomics, proteomics, and other omics data from iPSC-derived cells can provide a comprehensive understanding of disease mechanisms and identify biomarkers for personalized diagnostics and treatment monitoring [623]. The use of iPSCs in personalized medicine holds immense promise for advancing patient care. iPSCs provide a powerful tool for disease modeling, drug screening, and regenerative therapies, enabling a more precise and tailored approach to treatment. While challenges exist, ongoing research and technological advancements, along with collaborative efforts between different stakeholders, will pave the way for the integration of iPSC-based personalized medicine into mainstream healthcare. With continued investment in iPSC research and responsible application, which can harness the full potential of iPSCs to transform the landscape of personalized medicine, offering patients more effective, targeted, and personalized treatment options [568, 575, 584].

### The use of iPSCs in cancer early detection and diagnosis

**Fig. 11:**
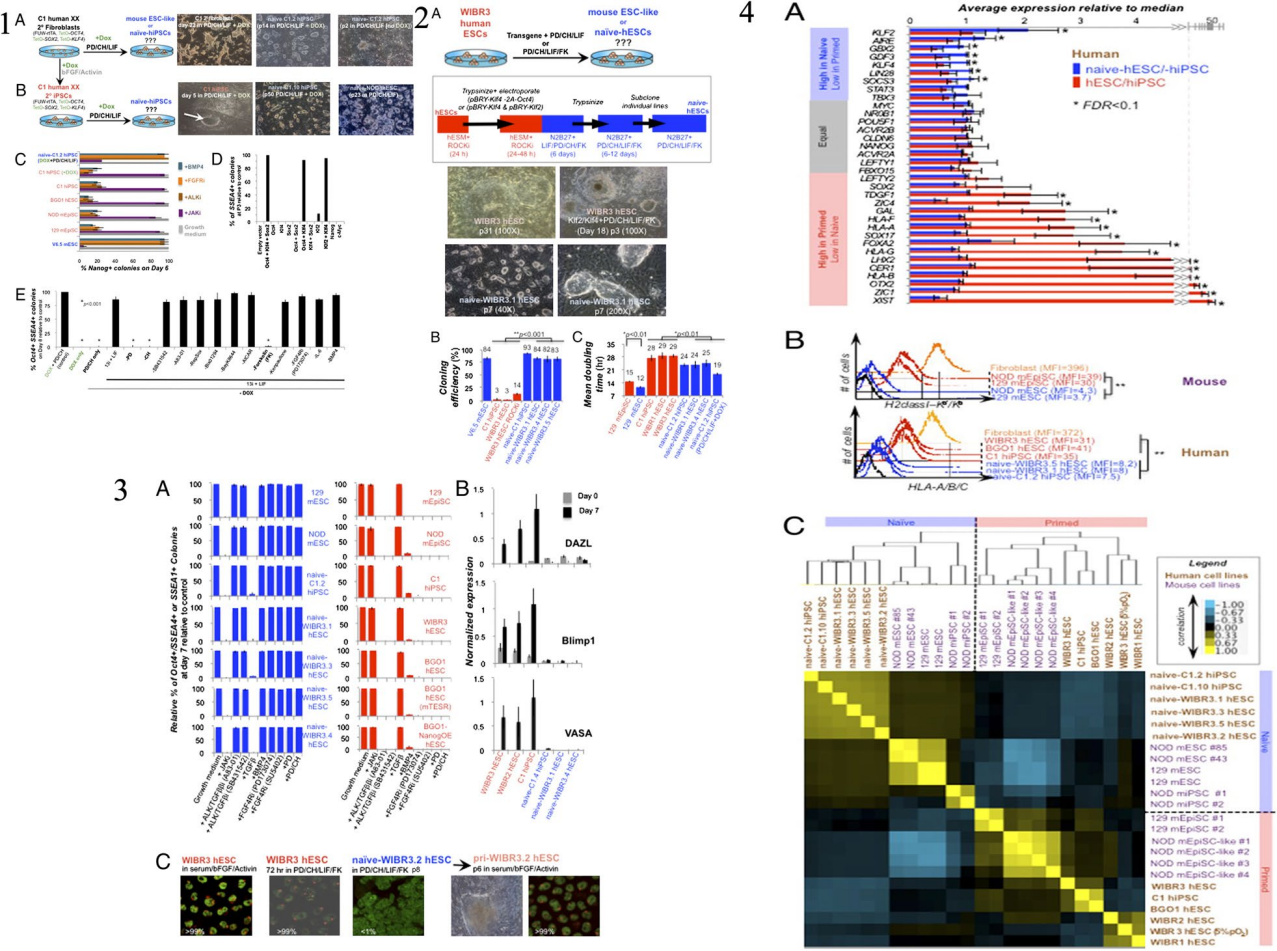
**1** The process of deriving iPSCs with characteristics similar to naïve mouse embryonic stem cells (mESCs). Panel A shows the overall strategy and representative images of C1 cultures and a subcloned cell line called C1.2 at various reprogramming stages. The passage number (p) is indicated. Additionally, images of NOD mESCs (mouse ESCs) and C1.2 hiPSCs (human iPSCs derived from C1) after withdrawal of doxycycline (DOX) are presented. Panel B demonstrates the maintenance of the C1 hiPSC line in a conventional growth condition for human embryonic stem cells (hESCs) supplemented with basic fibroblast growth factor (bFGF) and serum. The C1 line is transferred to a medium called N2B27 PD/CH/LIF + DOX, and emerging colonies are subcloned. A representative clone called C1.10 hiPSC is shown. Panel C explores the signaling dependence of pluripotent cell lines. Pluripotent cells are divided equally and plated on feeders in different growth media that are typically used for maintaining these cell lines. After 36 h, the wells are treated with specific inhibitors or growth factors. After 6 days, the wells are fixed and stained for a pluripotency marker called Nanog to determine the relative percentage of pluripotent colonies. Colony formation is normalized to an internal control growth medium without inhibitors. Panel D focuses on the reprogramming process of the C1.2 hiPSC line. The cells are electroporated with mammalian expression vectors expressing specific reprogramming factors and subjected to puromycin selection. The cells are then passaged in a medium called PD/CH/LIF without DOX. The values indicate the relative percentage of SSEA4 + colonies obtained compared to control cells that were transfected with a polycistronic construct encoding Oct4, Klf4, and Sox2. Panel E investigates the screening of factors that enable the propagation of transgene-independent C1 hiPSCs, meaning these cells no longer require DOX for stabilization. The effects of removing individual factors from a pool of 13 small molecules or cytokines are examined on the survival and pluripotency maintenance of C1 hiPSCs. C1 cells are plated on feeders in N2B27 media with the indicated factors. The P values obtained using Student’s t-test indicate significant changes compared to cells grown in DOX/PD/CH/LIF conditions, which are defined as the control with 100% survival. **2** The characteristics of naïve human embryonic stem cell (hESC) lines. In panel A, a diagram outlines the process of reverting hESCs to generate naïve hESCs. Representative images of WIBR3 hESCs at different stages of the reversion process in the presence of PD (small molecule), CH (chemical), LIF (leukemia inhibitory factor), and FK (forskolin) are shown. The passage number (p) and magnifications of the captured images are indicated. Panel B presents the single-cell cloning efficiency of various pluripotent stem cell lines. This efficiency is determined by counting the number of wells containing colonies positive for Nanog (a pluripotency marker) after 7 days. Panel C displays the estimated cell doubling time. Plated cells were counted at 1, 4, and 7 days after plating in triplicates, and the increase in cell number was used to calculate the average doubling time. The error bars represent the standard deviation (SD), and the P values, determined using Student's t-test, indicate significant differences between the average values of hESC/hiPSC lines compared to the average values of naïve hESC/hiPSC lines. **3** The similarities in signaling and epigenetic characteristics between naïve human embryonic stem cells (hESCs) and mouse embryonic stem cells (mESCs). In panel A, the dependence of pluripotent cell lines on specific signaling pathways was assessed. After a 7-day period, the wells were fixed and stained to determine the percentage of colonies positive for pluripotency markers. Mouse stem cells were stained with SSEA1. The colony formation was normalized to a control growth medium without inhibitors, which was represented in the first left column. Normalized percentages below 5% were categorized as “sensitivity” to the presence of the supplemented inhibitor. Panel B shows the expression of early germ-cell markers through RT-PCR in the presence or absence of BMP4/7/8 cytokines. Lastly, in panel C, a representative analysis using fluorescence in situ hybridization (FISH) was conducted to examine the presence of XIST RNA (red) and Cot1 nuclear RNA (green). The Pri-WIBR3.2 cell line was analyzed after being passaged in conventional bFGF/serum-containing human ESC growth conditions. The numbers provided in the figure indicate the average percentage of XIST-positive nuclei counted. **4** The similarities in gene expression between naive human embryonic stem cells (hESCs) and naive human-hiPSCs with mouse embryonic stem cells (mESCs). (A) a bar chart comparing the expression levels of pluripotency and lineage-specific marker genes in hESCs and naive hESCs, with asterisks indicating genes that showed significant differences between the two groups of samples; (B) a fluorescence-activated cell sorting (FACS) analysis measuring the surface expression of human and mouse major histocompatibility complex (MHC) class I alleles, with a black graph representing the control isotype match; (C) a cross-species gene expression clustering depicting the grouping of mESCs and naive hESCs as distinct from mEpiSCs (mouse epiblast stem cells) and hESCs. The legend on the right explains that yellow and blue colors represent positive and negative correlations, respectively. The gene expression levels were clustered based on Spearman correlation and average linkage, with mouse samples labeled in purple and human samples labeled in brown. Reprinted from [628] with permission from the PNAS

The use of iPSCs in cancer early detection and diagnosis holds immense promise and potential. Cancer is a complex disease characterized by the accumulation of genetic and epigenetic alterations in cells, leading to uncontrolled proliferation and tumor formation [550, 554]. Early detection and accurate diagnosis are crucial for improving patient outcomes and implementing timely and effective treatment strategies [593]. iPSCs, with their unique properties and capabilities, offer a valuable tool in the quest for early cancer detection. iPSCs can be generated by reprogramming adult somatic cells, such as skin cells or blood cells, into a pluripotent state similar to embryonic stem cells. These iPSCs have the ability to differentiate into various cell types, including those found in different organs and tissues affected by cancer [568, 601]. This characteristic makes iPSCs an attractive candidate for modeling cancer initiation and progression. One of the key applications of iPSCs in cancer early detection is the generation of organoids or miniaturized organs in a dish [564, 603]. In a recent investigation led by Hanna et al., the focus was on creating iPSCs that closely resembled mouse embryonic stem cells (mESCs) in terms of their biological and epigenetic characteristics [628]. The study centered on employing a novel reprogramming approach to transform hESCs into a less mature state resembling mESCs. Figure 11 provides a visual representation of the iPSC generation process using this method. By inducing the expression of Oct4, Klf4, and Klf2 factors in hESCs and subjecting them to specific growth conditions, the researchers successfully generated what they referred to as “epigenetically converted cells.” These cells exhibited striking similarities to mESCs in terms of growth properties, gene expression patterns, and reliance on specific signaling pathways, as highlighted in Fig. 11. Additionally, Fig. 11 underscores the likenesses between naive hESCs and mESCs in signaling and epigenetic characteristics. Figure 11 further demonstrates the connection between naive hESCs, hiPSCs, and mESCs based on their transcriptional profiles, showcasing the success of the reprogramming approach in producing hiPSCs that closely mimic mESCs. These findings pave the way for a deeper understanding of pluripotency in humans and hold great promise for conducting disease-specific research using patient-derived iPSCs. The iPSCs can be directed to differentiate into specific cell types relevant to different cancer types, allowing the creation of organoids that closely mimic the structure and function of actual organs [556]. These organoids can be used to study the early stages of cancer development, providing researchers with a platform to investigate the molecular changes and cellular interactions that drive tumor formation. Furthermore, iPSC-derived organoids can be utilized for personalized medicine approaches in cancer diagnosis [130, 577, 629]. By obtaining patient-derived iPSCs, researchers can generate organoids that replicate the genetic and epigenetic makeup of the individual's tumor. This personalized organoid model can then be used to test the efficacy of different treatment options, helping clinicians identify the most effective therapeutic strategies for specific patients [630, 631]. This approach holds the potential to optimize treatment outcomes by tailoring therapies to the individual characteristics of each patient's cancer. In addition to organoid models, iPSCs can also be employed for the development of non-invasive diagnostic tools for cancer. Traditional cancer diagnostic methods often involve invasive procedures such as biopsies, which can be uncomfortable for patients and may carry associated risks. iPSCs offer a non-invasive alternative by allowing the generation of liquid biopsy models [376, 610]. iPSC-derived cells can be released into the bloodstream and collected for analysis, providing valuable information about the presence and characteristics of tumors [610]. Liquid biopsy-based approaches using iPSCs have the potential to revolutionize cancer diagnosis, enabling early detection and monitoring of disease progression in a minimally invasive manner [632]. Moreover, iPSCs can be genetically modified to express specific reporter genes or markers that are associated with cancer. These modified iPSCs can be used to develop sensitive and specific biosensors for the detection of cancer-related molecules or biomarkers in patient samples. By leveraging the unique properties of iPSCs, such as their ability to self-renew and differentiate, these biosensors can provide highly accurate and reliable diagnostic information [633, 634]. Despite the tremendous potential of iPSCs in cancer early detection and diagnosis, there are challenges that need to be addressed. One such challenge is the efficient and standardized generation of iPSCs from patient samples [490, 635]. The reprogramming process can be time-consuming and may require optimization to ensure the generation of high-quality iPSCs [636]. Additionally, the scalability of iPSC-based diagnostic approaches needs to be improved to enable their widespread clinical implementation [637]. Ethical and legal considerations also come into play when using iPSCs for cancer research and diagnosis. Ensuring informed consent and protecting patient privacy are paramount. Guidelines and regulations should be established to govern the collection, storage, and use of patient-derived iPSCs for diagnostic purposes, ensuring that ethical standards are upheld and patient rights are respected [557, 572]. The use of iPSCs in cancer early detection and diagnosis represents a promising avenue of research [623]. iPSC-derived organoids and liquid biopsy models offer tremendous potential in understanding the molecular mechanisms underlying cancer initiation and progression. These models can help identify key genetic and epigenetic alterations associated with specific cancer types, enabling the development of targeted therapies tailored to individual patients [633, 634]. Furthermore, iPSC-based diagnostic tools, such as biosensors and liquid biopsy approaches, hold the promise of non-invasive and highly sensitive detection of cancer-related molecules and biomarkers. These innovative techniques have the potential to revolutionize cancer diagnosis by providing early detection, monitoring disease progression, and assessing treatment response in a minimally invasive manner [630, 631]. By avoiding the need for invasive procedures like biopsies, iPSC-based diagnostics can significantly improve patient comfort and reduce associated risks. Moreover, iPSCs can be used in conjunction with other diagnostic modalities, such as imaging techniques, to enhance the accuracy and reliability of cancer diagnosis. Combining iPSC-based models with imaging technologies allows for a comprehensive analysis of tumor characteristics, including size, location, and molecular features [633, 634]. This integrative approach can provide clinicians with a more comprehensive understanding of the disease, aiding in the selection of appropriate treatment strategies [630, 631]. Despite the remarkable potential of iPSCs in cancer early detection and diagnosis, several challenges need to be overcome before their widespread clinical implementation. Standardizing the generation of iPSCs and optimizing the differentiation protocols to produce organoids that faithfully recapitulate the complexity of real tumors are essential steps. Additionally, large-scale validation studies are necessary to assess the sensitivity, specificity, and reliability of iPSC-based diagnostic approaches across different cancer types and patient populations [630, 631]. Furthermore, regulatory frameworks and guidelines should be established to address the ethical and legal considerations associated with iPSC research [633, 634]. Ensuring informed consent, protecting patient privacy, and adhering to ethical standards are paramount when utilizing iPSCs in cancer research and diagnostics. Collaboration between researchers, clinicians, ethicists, and regulatory authorities is crucial to develop guidelines that safeguard patient rights and promote responsible and ethical use of iPSCs [610]. The iPSCs offer a promising avenue for early cancer detection and diagnosis. Their ability to generate organoids, develop biosensors, and serve as liquid biopsies opens up new possibilities for understanding the molecular basis of cancer and implementing personalized treatment strategies. While there are challenges to overcome, continued investment in iPSC research and collaborative efforts among stakeholders can pave the way for the clinical translation of iPSC-based approaches, ultimately leading to improved cancer outcomes and patient care [633, 634].

## Future of iPSCs in tumorigenesis

The future of iPSCs in tumorigenesis holds great promise, offering opportunities for advancements in precision medicine, the development of new therapies, the creation of more advanced models for research, and integration with other emerging technologies [549, 556, 638]. The future of iPSCs in tumorigenesis research is extremely promising. iPSCs have the potential to advance precision medicine by providing patient-specific models for personalized therapies and enabling the identification of specific genetic and epigenetic alterations associated with cancer [576]. The development of new cancer therapies is also within reach, as iPSCs can be utilized for drug screening, gene therapy, and the production of immune cells with enhanced anti-tumor properties [551, 555, 584, 596]. However, further advancements are needed in the development of more advanced iPSC models that accurately mimic the complex tumor microenvironments and facilitate the study of tumor heterogeneity and therapeutic resistance. Integrating iPSCs with other emerging technologies, such as gene editing, single-cell sequencing, and bioengineering, will further enhance our understanding of tumorigenesis and aid in the identification of novel biomarkers and therapeutic targets [130, 629, 639]. The continued investment in iPSC research, along with collaborations between scientists and clinicians, will be crucial in unlocking the full potential of iPSCs in tumorigenesis and therapy. The future of iPSCs in tumorigenesis research is bright, offering exciting prospects for precision medicine, the development of innovative therapies, advanced models for research, and integration with other cutting-edge technologies. As scientists continue to explore the vast potential of iPSCs, we can look forward to groundbreaking discoveries that will shape the field of cancer research and ultimately improve patient outcomes.

### iPSCs have the potential to revolutionize cancer precision medicine

These cells can be generated from patients' own somatic cells, allowing for the creation of patient-specific iPSC lines [612, 640]. This opens up avenues for personalized medicine approaches in cancer treatment (Table 9). By deriving iPSCs from cancer patients, researchers can study the molecular changes that occur during tumorigenesis and identify specific genetic and epigenetic alterations associated with the disease [15, 24]. iPSCs can be differentiated into various cell types, including cancer cells, which enables the development of personalized therapies targeting the specific cancer subtypes present in individual patients [24]. The use of iPSCs in precision medicine holds the promise of tailoring treatments to individual patients based *9.2.*

### iPSCs offer the potential to develop new cancer therapies

Through iPSC-based technologies, researchers can generate large quantities of patient-specific cells, including cancer cells, which can be used for drug screening and testing [584]. This allows for the identification of novel compounds or combinations of drugs that are more effective in targeting cancer cells while minimizing the toxicity on healthy tissues [585, 626]. iPSCs can also be genetically engineered to correct cancer-associated mutations or introduce therapeutic genes, providing a platform for gene therapy approaches [555]. Additionally, iPSCs can be utilized to produce immune cells with enhanced anti-tumor properties, facilitating the development of immunotherapies for cancer treatment. The versatility and adaptability of iPSCs make them a valuable tool in the development of innovative cancer therapies [551, 583, 599].

**Table 9:** The transformative role of iPSCs in advancing cancer precision medicine

Benefit	Description	Explanation	Example	Challenges	Column 6: Solution	References
Personalized Therapies	iPSCs can be derived from a patient's own cells, allowing for the creation of personalized cancer models and tailored treatment strategies	iPSCs can capture the genetic and epigenetic diversity of an individual's cancer, enabling more effective and targeted therapies	Generating iPSCs from a cancer patient's tissue and differentiating them into cancer-specific cell types	Ethical and logistical challenges in obtaining and handling patient-derived samples	Establish standardized protocols for iPSC generation and differentiation to streamline the process	[543]
Drug Screening	iPSCs can be used to develop patient-specific cancer models for drug screening, helping identify the most effective treatments with minimal side effects	This approach reduces the risk of adverse reactions and enhances treatment efficacy by simulating the patient's response to different drugs	Cultivating iPSC-derived cancer cells in a lab setting and exposing them to various drug candidates	Variability in iPSC quality and differentiation efficiency	Invest in improved differentiation protocols and quality control measures for iPSC lines	[24]
Cancer Biology Research	iPSCs enable researchers to study cancer initiation, progression, and metastasis in a controlled environment, facilitating a deeper understanding of the disease	By using iPSCs, scientists can dissect the molecular mechanisms underlying cancer development and identify potential targets for therapy	Inducing iPSCs to develop into specific cancer cell types and conducting in-depth molecular analyses	Complex interactions within cancer cell populations and microenvironments	Develop 3D culture models that better mimic the tumor microenvironment for more accurate research	[545]
Gene Editing and CRISPR	iPSCs can be genetically modified using CRISPR-Cas9 technology to study the impact of specific gene mutations and test potential gene therapies	This approach allows for precise manipulation of genes in iPSC-derived cancer cells, aiding in the development of targeted therapies	Introducing CRISPR-edited mutations into iPSCs and observing their effects on cancer-related pathways	Off-target effects and low editing efficiency in some cases	Improve CRISPR technology for higher precision and efficiency in iPSCs	[546]
Disease Modeling	iPSCs can be differentiated into various cell types to model different cancer types, helping researchers study rare or hard-to-access cancers	This expands the scope of cancer research and allows for a broader understanding of the disease	Generating iPSC-derived models of specific cancer types and studying their characteristics and responses	Variability in differentiation protocols and challenges in mimicking the complexity of real tumors	Collaborate with experts in specific cancer types to refine differentiation protocols and model systems	[546]
Cell Therapy Development	iPSCs can serve as a source for generating patient-specific immune cells or therapeutic cells for cancer treatment, potentially improving the safety and effectiveness of cell-based therapies	Using iPSC-derived immune or therapeutic cells can reduce the risk of graft-versus-host disease and enhance the compatibility of cell-based therapies	Differentiating iPSCs into the desired therapeutic cell type and ensuring their safety and efficacy in preclinical studies	Immune rejection and potential tumorigenicity of iPSC-derived cells	Explore methods to enhance immunocompatibility and safety of iPSC-derived cell therapies	[545]

### iPSCs offer the potential to develop new cancer therapies

Through iPSC-based technologies, researchers can generate large quantities of patient-specific cells, including cancer cells, which can be used for drug screening and testing [610]. This allows for the identification of novel compounds or combinations of drugs that are more effective in targeting cancer cells while minimizing the toxicity on healthy tissues. iPSCs can also be genetically engineered to correct cancer-associated mutations or introduce therapeutic genes, providing a platform for gene therapy approaches [542]. Additionally, iPSCs can be utilized to produce immune cells with enhanced anti-tumor properties, facilitating the development of immunotherapies for cancer treatment. The versatility and adaptability of iPSCs make them a valuable tool in the development of innovative cancer therapies [555, 606].

### The development of more advanced iPSC models for tumorigenesis research is a crucial area of focus

Current iPSC models provide valuable insights into the early stages of cancer development and progression. However, there is a need for more sophisticated models that accurately recapitulate the complex dynamics of tumor microenvironments, including interactions with stromal cells, immune cells, and extracellular matrices. Advances in tissue engineering and organoid technology are being integrated with iPSCs to create 3D tumor models that better mimic the physiological conditions of tumors in vivo [324]. These advanced models can enhance our understanding of tumor heterogeneity, therapeutic resistance, and metastasis, enabling the development of more effective treatment strategies [125]. Furthermore, the integration of patient-derived iPSCs with advanced genomic and proteomic analyses can provide comprehensive molecular characterization of tumors, aiding in the identification of novel biomarkers and therapeutic targets [641, 642].

### The integration of iPSCs with other emerging technologies in cancer research holds significant potential

iPSCs can be combined with gene editing techniques, such as CRISPR-Cas9, to introduce precise genetic modifications or perform functional genomics studies to elucidate the role of specific genes in tumorigenesis [606, 638, 643]. Moreover, the integration of iPSCs with single-cell sequencing technologies allows for the analysis of individual cancer cells, revealing heterogeneity and clonal evolution within tumors [639]. This information can guide treatment decisions and identify novel targets for therapy. Furthermore, the integration of iPSCs with bioengineering approaches, such as microfluidics and organ-on-a-chip systems, enables the study of tumor cell migration, invasion, and response to therapeutic agents in more physiologically relevant environments [540]. By combining iPSCs with these emerging technologies, researchers can gain deeper insights into the mechanisms underlying tumorigenesis and develop innovative strategies for cancer diagnosis and treatment. Table 10 provides an insightful overview of emerging technologies in iPSC-based cancer research.

**Table 10:** An overview of the emerging technologies in iPSC-based cancer research

Technology	Description	Potential to Advance the Field	References
CRISPR/Cas9	A gene-editing tool that allows precise modification of DNA	Enables targeted gene editing and functional genomics studies, leading to a better understanding of cancer biology and potential therapeutic targets	[644]
Single-cell RNA sequencing	Sequencing technique that provides transcriptomic data at the single-cell level	Allows identification of cellular heterogeneity within tumors, enabling the characterization of rare cell populations, identification of tumor subtypes, and monitoring of tumor evolution. It can provide insights into the mechanisms of drug resistance and aid in the development of personalized therapies	[611]
Organoid models	Three-dimensional cell cultures that mimic the structure and function of organs	Offers a more physiologically relevant model for studying tumor behavior and response to therapies. Organoids can be derived from patient-specific iPSCs, providing a platform for personalized medicine and drug screening	[95]
Liquid biopsy	Non-invasive analysis of circulating tumor DNA and other biomarkers in blood	Allows monitoring of tumor dynamics and genetic alterations through simple blood tests. Liquid biopsy can provide information on tumor heterogeneity, treatment response, and minimal residual disease, aiding in early detection, treatment selection, and disease monitoring	[645]
High-throughput screening	Automated methods to rapidly test large numbers of molecules or compounds	Accelerates the discovery of new therapeutic targets and drug candidates. High-throughput screening can identify compounds that selectively target cancer cells or modulate specific signaling pathways, facilitating the development of novel treatments and precision medicine approaches	[585]
Single-cell imaging	Advanced microscopy techniques for visualizing cellular processes at the single-cell level	Provides spatial and temporal information about cellular interactions, signaling pathways, and dynamic processes within tumors. Single-cell imaging can uncover new insights into tumor heterogeneity, microenvironment interactions, and therapeutic responses, informing the development of more effective cancer therapies	[634]
Machine learning	Artificial intelligence algorithms that learn from and make predictions or decisions based on data	Enables the analysis of large-scale datasets and extraction of patterns, facilitating the identification of novel biomarkers, prediction of treatment outcomes, and discovery of therapeutic targets. Machine learning can enhance precision medicine approaches by integrating diverse data types and optimizing treatment strategies for individual patients	[646]
Tumor-on-a-chip	Microfluidic platforms that recreate tumor microenvironments and interactions	Mimics the complex architecture and cellular interactions within tumors, allowing the study of tumor growth, invasion, and response to therapies. Tumor-on-a-chip models can aid in drug screening, personalized medicine, and understanding the mechanisms of metastasis, facilitating the development of targeted therapies and improving treatment efficacy	[647]
Spatial transcriptomics	Technique that combines spatial information with transcriptomic profiling	Enables the mapping of gene expression patterns within tissue sections, providing spatial context to molecular data. Spatial transcriptomics can reveal the cellular composition and organization of tumors, identify spatially restricted gene expression patterns, and uncover novel biomarkers or therapeutic targets. It enhances our understanding of tumor heterogeneity and microenvironmental interactions, guiding the development of more precise and effective cancer therapies	[648]
Nanotechnology	Application of nanoscale materials and devices in cancer research	Offers targeted drug delivery systems, imaging agents, and sensors for early detection. Nanotechnology can improve treatment efficacy by enhancing drug delivery to tumors, monitoring therapeutic response, and enabling personalized medicine. Additionally, nanoscale platforms can be used for diagnostic purposes, such as detecting circulating tumor cells or analyzing cancer-related biomarkers	[649]
Immunotherapy	Treatment approach that enhances the immune system's ability to fight cancer	Harnesses the body's immune response to target and destroy cancer cells. Immunotherapies, including immune checkpoint inhibitors and CAR-T cell therapy, have shown remarkable success in certain cancer types and have the potential to revolutionize cancer treatment by providing durable and specific responses	[217]
Epigenetic modifications	Alterations in gene expression patterns without changing the underlying DNA sequence	Studying epigenetic modifications can shed light on the regulatory mechanisms of cancer development and progression. Understanding the epigenetic landscape of tumors can lead to the identification of novel therapeutic targets and the development of epigenetic-based therapies	[650]
Metabolomics	Analysis of metabolites in biological systems	Provides insights into the metabolic rewiring of cancer cells, identifying metabolic vulnerabilities and potential targets for therapeutic intervention. Metabolomics can also aid in biomarker discovery, patient stratification, and monitoring treatment response	[651]
Artificial intelligence	Simulation of human intelligence in machines	Offers powerful tools for data analysis, image recognition, and decision-making in cancer research. AI can assist in image interpretation, drug discovery, treatment optimization, and predicting patient outcomes based on various data types. Integrating AI into clinical practice has the potential to enhance diagnostics, treatment planning, and patient management	[611]
3D bioprinting	Fabrication of three-dimensional tissues or organ-like structures	Enables the creation of patient-specific tumor models for drug screening and personalized medicine. 3D bioprinting can replicate the complex architecture and microenvironment of tumors, allowing researchers to study tumor biology, drug responses, and potential treatment strategies in a more clinically relevant context	[652]
Genome editing	Precision modification of DNA sequences	Techniques like CRISPR/Cas9 enable targeted editing of cancer-related genes, providing insights into their function and potential therapeutic interventions. Genome editing can be used to correct genetic mutations, disrupt oncogenes, or introduce therapeutic genes, opening up new possibilities for precise cancer treatments	[6]
Multi-omics integration	Integration of multiple types of omics data (genomics, transcriptomics, proteomics, etc.)	Combining different omics datasets allows for a comprehensive understanding of cancer biology. Multi-omics integration enables the identification of key molecular pathways, biomarkers, and potential therapeutic targets, paving the way for personalized treatment strategies and precision medicine	[653]
Tumor microenvironment	Study of the cellular and non-cellular components surrounding tumors	Investigating the tumor microenvironment provides insights into its role in tumor growth, invasion, metastasis, and response to therapies. Understanding the complex interactions between cancer cells, immune cells, stromal cells, and the extracellular matrix can lead to the development of targeted therapies that disrupt or modulate these interactions, improving treatment outcomes	[654]

## Case studies

The case studies mentioned emphasize the successful use of iPSCs in studying the development of cancer and their significant impact on the advancement of cancer therapies. iPSCs provide unique opportunities in various areas, including modeling diseases [15], tailoring treatments, screening drugs [556], regenerative medicine [125], immunotherapies [608], early cancer detection, and precision medicine [24]. By serving as a platform to investigate the complex mechanisms involved in cancer development, iPSCs have expanded our knowledge of the genetic, epigenetic, and environmental factors contributing to cancer [545]. Furthermore, iPSCs have played a vital role in developing individualized and targeted therapies, offering great potential for improving patient outcomes and reducing the overall burden of cancer [531]. As research progresses in the field of iPSCs and tumorigenesis, it is important to address the challenges and limitations associated with iPSC-based approaches. This includes enhancing the efficiency of iPSC generation, refining differentiation protocols to generate specific cell types more effectively, and considering the ethical and regulatory aspects of iPSC research [545]. Continued investment in iPSC research and fostering collaboration among scientists, clinicians, and policymakers will further unlock the full potential of iPSCs in cancer research and therapy development. This collaborative effort holds the promise of devising better strategies for cancer prevention, early detection, and personalized treatment approaches that can greatly impact the field of oncology. Table 11 highlights several significant case studies in cancer research that have utilized iPSCs.

**Table 11:** Significant iPSC-based cancer research case studies

Study name	Results	Impact on understanding of tumorigenesis	References
iPSCs as a tool for modeling cancer development and progression	Generated iPSCs from cancer cells and analyzed their behavior in vitro and in vivo, providing a new platform for studying cancer biology and identifying potential therapeutic targets	Demonstrated that iPSCs can be used to model cancer development and progression, enabling researchers to study the disease in a more controlled and reproducible manner	[15, 290]
Characterization of oncogenic mutations in iPSC-derived cancer cells	Used CRISPR-Cas9 gene editing to introduce specific oncogenic mutations into iPSCs and analyzed their effect on cancer development and progression	Revealed the specific genetic mutations that can lead to cancer development and provided a new platform for testing potential cancer therapies	[655, 656]
iPSC-based drug screening for cancer therapy	Screened a library of compounds using iPSC-derived cancer cells and identified several novel drug candidates with anti-cancer activity	Provided a new approach to drug screening for cancer therapy that can improve the efficiency and efficacy of drug discovery	[657, 658]
Comparison of iPSC-derived cancer cells to primary tumor samples	Analyzed the similarities and differences between iPSC-derived cancer cells and primary tumor samples, providing insights into the limitations and potential of iPSC-based cancer research	Highlighted the importance of validating iPSC-derived cancer models against primary tumor samples to ensure their relevance and accuracy	[24]
iPSC-based modeling of cancer metastasis	Generated iPSCs from metastatic cancer cells and analyzed their behavior in vitro and in vivo, providing new insights into the mechanisms underlying cancer metastasis	Revealed the specific genetic and cellular changes that enable cancer cells to metastasize, providing potential targets for developing therapies to prevent cancer spread	[290, 544, 606, 659]
iPSC-based modeling of cancer immunotherapy	Generated iPSCs from patient-derived cancer cells and engineered them to express immune checkpoint inhibitors, providing a new platform for studying the effectiveness of immunotherapy in treating cancer	Demonstrated the potential of iPSC-based models to improve the development and optimization of cancer immunotherapies	[25, 660]
iPSC-based modeling of cancer heterogeneity	Generated iPSCs from different types of cancer cells and analyzed their behavior in vitro and in vivo, providing insights into the heterogeneity of cancer and the importance of personalized medicine	Highlighted the need for personalized medicine approaches that take into account the heterogeneity of cancer and the individual characteristics of each patient's disease	[533, 661]
iPSC-based modeling of drug resistance in cancer	Generated iPSCs from drug-resistant cancer cells and investigated the underlying mechanisms of resistance, identifying potential strategies to overcome it	Provided insights into the molecular mechanisms of drug resistance in cancer, leading to the development of more effective treatment approaches	[662–664]
Investigation of epigenetic modifications in iPSC-derived cancer cells	Analyzed epigenetic changes in iPSC-derived cancer cells compared to normal cells, revealing alterations in gene expression and potential epigenetic targets for therapy	Shed light on the role of epigenetic modifications in cancer development and progression, opening avenues for targeted epigenetic therapies	[216, 665, 666]
iPSC-based modeling of cancer stem cells	Generated iPSCs from cancer stem cells and characterized their properties, including self-renewal and differentiation capabilities, providing insights into their role in tumor initiation and treatment resistance	Enhanced understanding of cancer stem cells' contribution to tumor development, recurrence, and resistance to therapy, guiding the development of therapies targeting these cells	[216, 661, 667, 668]
iPSC-based modeling of tumor microenvironment interactions	Co-cultured iPSC-derived cancer cells with stromal cells to mimic the tumor microenvironment and studied the reciprocal interactions, uncovering the role of stromal cells in tumor growth and progression	Improved understanding of the dynamic interactions between cancer cells and the surrounding microenvironment, leading to the identification of potential therapeutic targets in the tumor microenvironment	[230, 401, 669]
iPSC-based modeling of inherited cancer predisposition syndromes	Generated iPSCs from patients with inherited cancer predisposition syndromes and investigated the molecular mechanisms underlying their increased cancer risk, aiding in the development of targeted prevention and early detection strategies	Provided insights into the genetic factors contributing to inherited cancer predisposition syndromes, facilitating personalized risk assessment and management strategies	[670]
iPSC-based modeling of tumor heterogeneity and clonal evolution	Generated iPSCs from different tumor subclones within a patient and tracked their evolution over time, revealing insights into tumor heterogeneity and clonal dynamics	Advanced our understanding of tumor evolution and the development of resistance, informing the design of combination therapies targeting multiple tumor subclones	[571, 671–673]
iPSC-based modeling of cancer dormancy and recurrence	Induced dormancy in iPSC-derived cancer cells and investigated the factors contributing to their reactivation, providing insights into mechanisms of cancer recurrence	Enhanced understanding of cancer dormancy and the potential drivers of tumor relapse, aiding in the development of strategies to prevent or target dormant cancer cells	[674]
iPSC-based modeling of cancer metabolism	Analyzed metabolic alterations in iPSC-derived cancer cells compared to normal cells, identifying metabolic vulnerabilities and potential targets for therapeutic intervention	Provided insights into the rewiring of cancer cell metabolism and the potential for targeting specific metabolic pathways in cancer treatment	[675, 676]
iPSC-based modeling of immune evasion mechanisms in cancer	Generated iPSCs from cancer cells and examined their interaction with immune cells, uncovering mechanisms of immune evasion and resistance to immunotherapy	Improved understanding of the complex interplay between cancer cells and the immune system, guiding the development of novel immunotherapeutic strategies	[677]
iPSC-based modeling of cancer-associated fibroblasts	Derived iPSCs from cancer-associated fibroblasts and investigated their role in tumor growth and metastasis, revealing the influence of the tumor microenvironment on cancer progression	Enhanced understanding of the interactions between cancer cells and cancer-associated fibroblasts, providing potential targets for therapeutic intervention	[678–680]
iPSC-based modeling of DNA repair defects in cancer	Generated iPSCs from patients with DNA repair deficiencies and examined their susceptibility to genomic instability and cancer development, highlighting the role of DNA repair mechanisms in tumor suppression	Provided insights into the link between DNA repair defects and cancer susceptibility, contributing to the development of personalized therapies for patients with specific DNA repair deficiencies	[681–683]
iPSC-based modeling of cancer cell dormancy and reactivation	Investigated the mechanisms underlying cancer cell dormancy and reactivation using iPSC-derived models, uncovering factors that control the switch between dormant and active states	Improved understanding of the processes involved in cancer cell dormancy and reactivation, providing potential targets for preventing tumor recurrence	[684, 685]
iPSC-based modeling of cancer-associated inflammation	Generated iPSCs from cancer cells and studied their interaction with inflammatory cells, elucidating the role of inflammation in cancer development and progression	Enhanced understanding of the crosstalk between cancer cells and the inflammatory microenvironment, paving the way for the development of anti-inflammatory strategies for cancer treatment	[654, 686, 687]
iPSC-based modeling of therapeutic resistance in cancer	Created iPSCs from cancer cells resistant to specific therapies and investigated the molecular mechanisms underlying the resistance, identifying potential strategies to overcome treatment resistance	Provided insights into the mechanisms of therapeutic resistance in cancer, guiding the development of combination therapies and personalized treatment approaches	[688, 689]
iPSC-based modeling of cancer-induced immunosuppression	Derived iPSCs from cancer cells and immune cells and studied their interactions, revealing the immunosuppressive mechanisms employed by cancer cells to evade immune surveillance	Improved understanding of the mechanisms of cancer-induced immunosuppression, facilitating the development of immunotherapeutic approaches to enhance anti-tumor immune responses	[690–692]
iPSC-based modeling of cancer cell plasticity and lineage reprogramming	Induced lineage reprogramming in iPSC-derived cancer cells and investigated their potential to acquire different cell fates, providing insights into cancer cell plasticity and transdifferentiation	Advanced our understanding of the phenotypic plasticity of cancer cells and their ability to switch between different cell types, contributing to the development of targeted therapies	[280, 693]
iPSC-based modeling of cancer metastatic niche formation	Generated iPSCs from metastatic cancer cells and investigated their interactions with the microenvironment at metastatic sites, uncovering the processes involved in the formation of premetastatic niches	Enhanced understanding of the interactions between metastatic cancer cells and the microenvironment, opening up new avenues for the prevention and treatment of cancer metastasis	[694, 695]
iPSC-based modeling of cancer cell invasion and migration	Generated iPSCs from invasive cancer cells and investigated their migratory behavior, revealing the molecular mechanisms involved in cancer cell invasion and migration	Improved understanding of the processes driving cancer cell invasion and migration, providing potential targets for inhibiting metastasis	[696, 697]
iPSC-based modeling of tumor angiogenesis	Derived iPSCs from cancer cells and endothelial cells to study the process of tumor angiogenesis, uncovering the factors and signaling pathways involved in promoting blood vessel formation in tumors	Enhanced understanding of the mechanisms underlying tumor angiogenesis, facilitating the development of anti-angiogenic therapies for cancer treatment	[533, 661]
iPSC-based modeling of immune cell interactions in the tumor microenvironment	Generated iPSCs from immune cells and cancer cells to study their interactions within the tumor microenvironment, elucidating the dynamics of immune cell infiltration, activation, and suppression	Advanced our understanding of the complex interplay between immune cells and cancer cells in the tumor microenvironment, guiding the development of immunotherapeutic strategies	[30, 698, 699]
iPSC-based modeling of cancer cell metabolism rewiring	Analyzed the metabolic profile of iPSC-derived cancer cells and identified metabolic alterations and rewiring associated with cancer development and progression	Provided insights into the metabolic adaptations of cancer cells, offering potential targets for metabolic therapies in cancer treatment	[700]
iPSC-based modeling of cancer cell senescence and aging	Induced cellular senescence in iPSC-derived cancer cells and studied the effects of senescence on cancer cell behavior and response to therapy, revealing the impact of cellular aging on tumor progression and treatment outcomes	Enhanced understanding of the role of senescence in cancer cell biology and its implications for therapeutic interventions	[701, 702]
iPSC-based modeling of cancer-associated pain	Generated iPSCs from patients with cancer-associated pain and investigated the mechanisms underlying pain perception in cancer, identifying potential targets for pain management and relief	Provided insights into the molecular and cellular basis of cancer-associated pain, contributing to the development of personalized pain management strategies	[703, 704]
iPSC-based modeling of immune cell therapies for cancer	Generated iPSCs from patient-derived immune cells and engineered them to express chimeric antigen receptors (CARs) or T cell receptors (TCRs), providing a platform for studying the efficacy and safety of immune cell therapies	Advanced our understanding of immune cell therapies for cancer, facilitating the development of improved strategies for enhancing anti-tumor immune responses	[705, 706]
iPSC-based modeling of cancer-associated fibrosis	Derived iPSCs from fibroblasts within tumor tissues and studied their role in promoting cancer-associated fibrosis, revealing the mechanisms underlying fibrotic changes in the tumor microenvironment	Improved understanding of the role of cancer-associated fibrosis in tumor progression and resistance to therapy, suggesting potential targets for intervention	[680, 707]
iPSC-based modeling of cancer metabolism heterogeneity	Analyzed metabolic variations among iPSC-derived cancer cells from different patients and tumor types, uncovering metabolic heterogeneity in cancer and its implications for personalized treatment approaches	Enhanced understanding of the metabolic diversity of cancer cells, guiding the development of tailored metabolic therapies	[708, 709]
iPSC-based modeling of cancer cell dormancy in bone marrow niches	Co-cultured iPSC-derived cancer cells with bone marrow stromal cells to mimic the bone marrow microenvironment and investigated the mechanisms underlying cancer cell dormancy in bone metastases	Provided insights into the factors contributing to cancer cell dormancy in the bone marrow and potential strategies to target dormant cells for improved treatment outcomes	[710, 711]
iPSC-based modeling of tumor immunosurveillance escape mechanisms	Derived iPSCs from tumor cells and investigated the mechanisms by which cancer cells evade immune recognition and destruction, revealing the strategies employed by tumors to evade immunosurveillance	Advanced our understanding of immune evasion mechanisms in cancer, informing the development of immunotherapies to overcome immune escape	[712, 713]
iPSC-based modeling of tumor suppressor gene mutations in cancer	Generated iPSCs from patients with inherited or acquired tumor suppressor gene mutations and studied the consequences of these mutations on cancer development and progression	Provided insights into the role of tumor suppressor genes in cancer pathogenesis, aiding in the identification of potential therapeutic targets	[533, 714]
iPSC-based modeling of cancer cell immune evasion through immune checkpoint pathways	Generated iPSCs from cancer cells and investigated the expression and regulation of immune checkpoint molecules, providing insights into the mechanisms of immune evasion in cancer	Enhanced understanding of the role of immune checkpoint pathways in cancer immune evasion, informing the development of targeted immunotherapies	[715]
iPSC-based modeling of cancer cell metabolism and microenvironment interactions	Co-cultured iPSC-derived cancer cells with different cell types representing the tumor microenvironment and studied their metabolic interactions, revealing the metabolic crosstalk between cancer cells and surrounding cells	Improved understanding of the metabolic interplay within the tumor microenvironment, suggesting potential metabolic targets for cancer therapy	[716, 717]
iPSC-based modeling of cancer cell resistance to radiation therapy	Induced iPSC-derived cancer cells to undergo radiation treatment and investigated the mechanisms of radioresistance, uncovering factors contributing to cancer cell survival and resistance to radiation therapy	Provided insights into the mechanisms underlying resistance to radiation therapy, guiding the development of strategies to overcome radioresistance	[718, 719]
iPSC-based modeling of tumor dormancy and recurrence after therapy	Generated iPSCs from residual tumor cells following treatment and studied their ability to reinitiate tumor growth, providing insights into the mechanisms of tumor dormancy and recurrence	Advanced our understanding of tumor dormancy and recurrence, facilitating the development of strategies to prevent tumor relapse after therapy	[720, 721]
iPSC-based modeling of cancer cell metabolism and therapeutic response	Analyzed the metabolic profile of iPSC-derived cancer cells and correlated it with their response to different therapies, identifying metabolic markers predictive of therapeutic outcomes	Provided insights into the relationship between cancer cell metabolism and treatment response, guiding the development of personalized treatment strategies	[722–724]
iPSC-based modeling of cancer cell heterogeneity and clonal evolution during therapy	Generated iPSCs from multiple tumor subclones within a patient and studied their response to therapy, revealing the dynamics of clonal evolution and the emergence of resistant subclones	Improved understanding of tumor heterogeneity and clonal evolution during therapy, informing the design of combination therapies and strategies to prevent therapy resistance	[671, 725]

### iPSC-based disease modeling

**Fig. 12:**
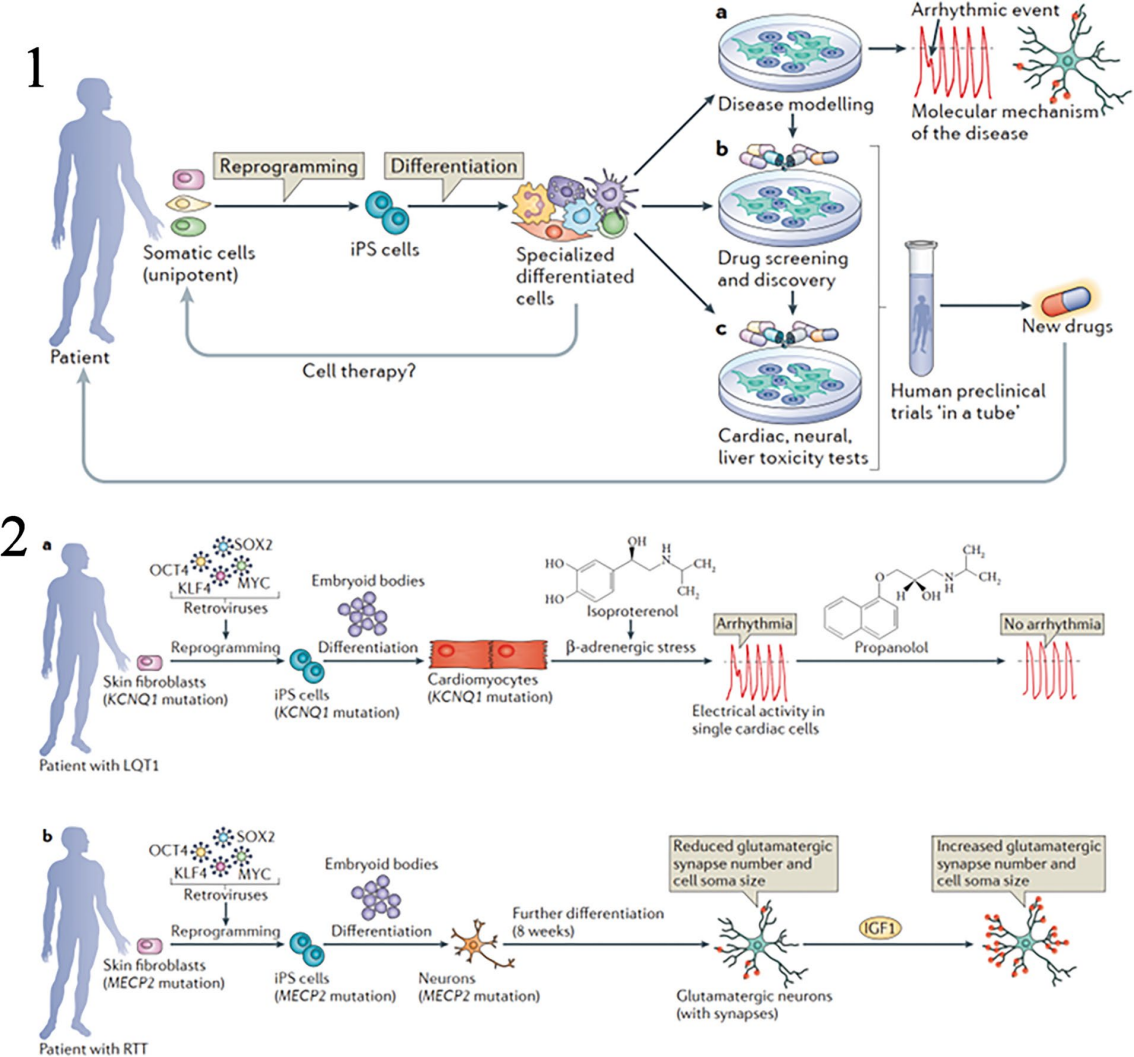
**1** The process of deriving and utilizing human induced pluripotent stem (iPS) cells. It shows that adult somatic cells, which are specialized cells in the body, can be reprogrammed into iPS cells capable of differentiating into various cell types. These iPS cells have several applications. a) One application is disease modeling, where human iPS cells are used to investigate the molecular mechanisms behind disease phenotypes. For example, they can be employed to study the molecular causes of arrhythmia in cardiomyocytes or defects in neurogenic differentiation. b) Human iPS cells can also be utilized in drug screening and discovery. They help determine the effects of candidate drugs and new compounds and identify target pathways. c) Another valuable application of human iPS cells is in conducting toxicity tests for cardiac, neural, and liver cells. These tests assess the toxic responses of cells to drugs and substances. Combining drug screening and toxicity tests allows for human preclinical trials in a controlled laboratory setting, enabling early involvement of “the patient” in the drug discovery process. **2** The use of human induced pluripotent stem (iPS) cells for modeling cardiac and neural diseases and the improvement of disease symptoms. In the first scenario (a), skin fibroblasts taken from a patient with type 1 long QT syndrome (LQT1), which is caused by a mutation in the KCNQ1 potassium channel gene, were reprogrammed into iPS cells using retroviral transduction of four specific genes. These iPS cells were then transformed into clusters called embryoid bodies and subsequently differentiated into cardiomyocytes. The presence of spontaneous contraction in these cells indicated the existence of functioning heart muscle cells. By applying isoprenaline, a substance that mimics β-adrenergic stress, arrhythmic events similar to those observed in LQT1 patients' hearts were induced in the cardiomyocytes. However, when the β-blocker propranolol was administered, the arrhythmia was suppressed. In the second scenario (b), skin fibroblasts were obtained from a patient with Rett syndrome (RTT), which is caused by a mutation in the MECP2 gene responsible for regulating epigenetic processes. These fibroblasts were reprogrammed into human iPS cells using retroviral transduction of the same four genes mentioned earlier. The iPS cells were then differentiated into embryoid bodies, and the appearance of rosette structures indicated the presence of neural precursors. Further differentiation of these precursors resulted in the formation of glutamatergic neurons. These neurons exhibited reduced numbers of glutamatergic synapses (represented by red dots) and a decrease in soma size (the cell body of the neuron). However, treatment with insulin-like growth factor 1 (IGF1) caused an increase in both the number of glutamatergic synapses and the size of the neuron's soma. Reprinted from [726] with permission from the Springer Nature

**Fig. 13:**
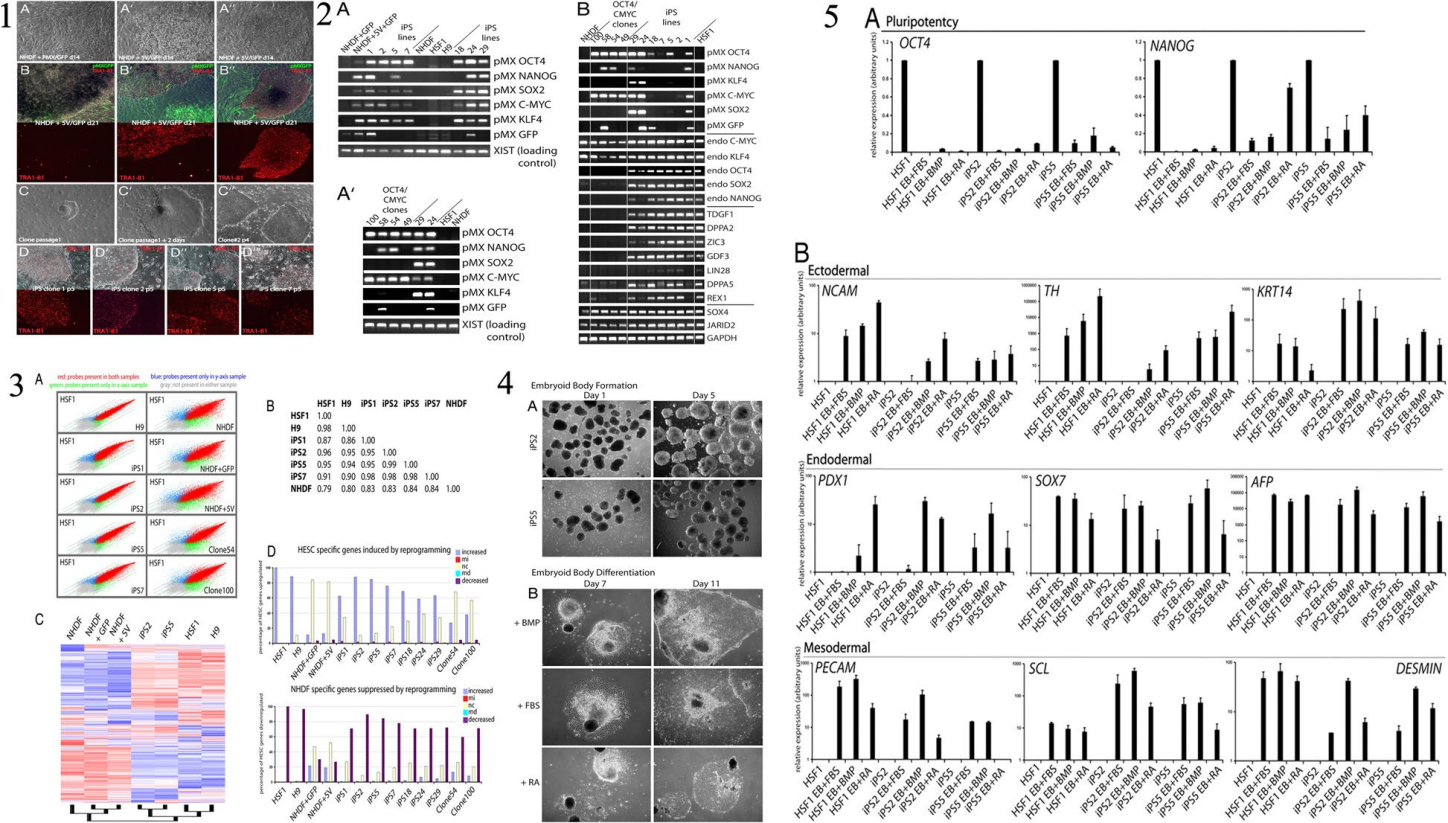
**1** The similarity in appearance between induced pluripotent stem (iPS) clones and human embryonic stem cells (HESC). In panel A', the figure shows colonies of NHDF1 (normal human dermal fibroblast) cells infected with different viruses. These viruses include an empty virus, a GFP-containing virus, or a combination of six viruses, each carrying one of five specific transcription factors or GFP. The colonies are observed under phase contrast microscopy, revealing their diverse morphologies. Panels B-B” provide phase-contrast images of specific colonies from the cultures transduced with the combination of five transcription factors and GFP. These images are merged with live TRA-1–81 staining (shown in red) and GFP fluorescence (shown in green) derived from the pMX-GFP virus. The upper images show the merged view, while the lower images display only the TRA-1–81 channel. It is noteworthy that only a small fraction of colonies exhibit TRA-1–81 positivity, as indicated in panels B and B'. Importantly, the TRA-1–81 staining in these positive colonies closely resembles that of HESC. Panels C–C” display phase-contrast images of iPS clones at different passages, highlighting their morphological characteristics. Finally, panels D-D”' present “live” TRA-1–81 staining merged with the phase-contrast appearance of specific iPS clones at passage 5. **2** The induced pluripotent stem (iPS) clones exhibit important markers found in human embryonic stem cells (HESC). In panel A and A', polymerase chain reaction (PCR) was performed on genomic DNA obtained from various sources: iPS clones, “early” OCT4/C-MYC clones, NHDF1 (normal human dermal fibroblast) cells infected with control or defined factor viruses, and HSF1 or H9 HESC. The PCR targeted specific regions of integrated viruses, with a loading control PCR for a genomic region on the X chromosome within the XIST locus. Additionally, iPS clones 24 and 29 were included in panel A' as a positive control for the PCR conditions. In panel B, reverse transcription PCR (RT-PCR) was conducted to analyze pMX retroviral transcription and the expression of endogenous counterparts of the defined factors, as well as other genes specific to HESC (TDGF1 through REX1), in iPS clones, NHDF1 cells, HSF1 HESC, and OCT4/CMYC clones. It is worth noting that iPS clones 24 and 29, as well as the OCT4/CMYC clones, displayed limited suppression of expression from the viruses they received. **3** The comparison of the transcriptome (gene expression profile) between induced pluripotent stem (iPS) clones and human embryonic stem cells (HESC). In panel A, the expression values of various cell types are presented on a scatter plot using genome-wide microarray expression data. The cell types include fibroblasts infected with control viruses or viruses carrying specific factors, iPS clones 2 and 5, and the HSF1 HESC line. It is observed that iPS clones 2 and 5 exhibit a high similarity to the HSF1 HESC, while iPS lines 1 and 7 show slightly less similarity. Panel B represents the global Pearson correlation analysis of the entire expression data between the different cell types, indicating the degree of similarity in gene expression. Panel C displays the hierarchical clustering of gene-expression data using the indicated cell types. The analysis involved normalization and expression analysis with DNA-chip analyzer (dChip), filtering genes based on a 20% presence call, and removing redundant probe sets. In panel D, the 2,000 most up- and down-regulated genes in HSF1 versus NHDF (normal human dermal fibroblast) were identified from genome-wide expression datasets. The expression of these genes was further analyzed to determine if they were up-regulated, down-regulated, or showed no change in expression between iPS clones (or infected fibroblast pools) and NHDF. The terms “MI” and “MD” represent statistically marginal increase and decrease, respectively. **4** The formation of embryoid bodies (EBs) by induced pluripotent stem (iPS) cells, which is comparable to human embryonic stem cells (HESCs). Panel A displays phase-contrast images of EBs created from iPS clones 2 and 5. Panel B demonstrates the growth of iPS-derived EBs when placed on adherent tissue culture dishes using three distinct media conditions. One of the media conditions includes the presence of bone morphogenetic protein 4 (BMP). **5** The pluripotency of induced pluripotent stem (iPS) cells and the increased expression of markers associated with ectoderm, endoderm, and mesoderm. In panel A, a real-time RT-PCR analysis compares the expression of pluripotency genes in iPS cells and control human embryonic stem cells (HESC) after inducing differentiation through embryoid body (EB) formation and subsequent plating under specific conditions (BMP4, FBS, and retinoic acid). The analysis measures the fold change in gene expression relative to the reference gene GAPDH. Notably, the down-regulation of pluripotency markers like OCT4 and NANOG is observed during EB differentiation. In panel B, a similar analysis is conducted, but this time the expression of marker genes associated with different germ layers is examined. Each marker is specific to a particular germ layer, as indicated. The y-axis represents the fold induction of gene expression compared to undifferentiated cells. While the extent of induction of lineage markers may vary between HESC and iPS clones, the overall pattern remains consistent. Reprinted from [728]with permission from the PNAS

Disease modeling involves generating in vitro models of human diseases to understand the underlying mechanisms, test drug efficacy, and develop new treatments. Traditionally, disease modeling relied on animal models or immortalized cell lines, both of which have limitations in recapitulating human disease due to differences in physiology, genetic makeup, and epigenetic regulation [553]. In a recent study, some researchers provided the remarkable potential of human induced pluripotent stem (iPS) cells for disease modeling was highlighted [726]. The researchers utilized iPS cells to model both cardiac and neural diseases, as depicted in Fig. 12 of their study. By reprogramming somatic cells from patients with these diseases, they were able to generate patient-specific iPS cells, which accurately recapitulated disease phenotypes in vitro. This breakthrough allowed for a deeper understanding of the underlying mechanisms and pathophysiology of these diseases. Additionally, Fig. 12 in the study illustrated the process of deriving and utilizing iPS cells, emphasizing their pivotal role in advancing regenerative medicine and drug discovery. The findings from this study underscore the immense potential of iPS cells in revolutionizing disease research and personalized medicine. The iPSCs offer several advantages over traditional disease models. Firstly, iPSCs can be derived directly from patients with a disease, allowing for the modeling of patient-specific diseases [543]. This is particularly relevant for diseases with a strong genetic component, such as cancer, where different mutations in the same gene can result in different cancer subtypes and response to treatment [543]. Secondly, iPSCs can be differentiated into different cell types, including disease-relevant cell types, such as neurons for neurodegenerative diseases, cardiomyocytes for cardiovascular diseases, and hepatocytes for liver diseases [247, 727]. This enables the generation of more physiologically relevant disease models that mimic the tissue-specific characteristics of the disease [543]. Lastly, iPSC-based disease models can be used to test drug efficacy and toxicity, potentially leading to the development of more effective and safer treatments [125, 626]. In a recent study conducted by Lowry et al., the researchers focused on generating human induced pluripotent stem (iPS) cells from dermal fibroblasts, with the aim of advancing stem cell-based therapies for degenerative diseases [728]. In a recent investigation led by Lowry and colleagues, the primary focus was on the generation of iPS cells from dermal fibroblasts, aiming to advance the use of stem cell-based therapies for degenerative diseases [728]. The study employed specific transcription factors, including KLF4, OCT4, SOX2, and C-MYC, to reprogram dermal fibroblasts into iPS cells. The outcomes, illustrated in Fig. 13, indicated a striking resemblance of the iPS cell clones to human embryonic stem cells (HESC) in terms of their visual characteristics. Furthermore, Fig. 13 presented evidence that these iPS clones expressed key markers typically associated with HESC, suggesting a molecular similarity. Transcriptome analysis, as shown in Fig. 13, further affirmed the likeness in gene expression profiles between iPS clones and HESC. Additionally, Fig. 13 provided evidence that iPS cells could form embryoid bodies (EBs) akin to HESC, and Fig. 13 emphasized the pluripotent nature of iPS cells by demonstrating their capacity to differentiate into ectoderm, endoderm, and mesoderm lineages. These findings underscore the pivotal role of iPSCs in offering an abundant source of patient-specific pluripotent stem cells with potential clinical applications.

### Case study: iPSC-based modeling of Familial Adenomatous Polyposis (FAP)

Familial adenomatous polyposis (FAP) is a rare hereditary disease characterized by the development of multiple adenomatous polyps in the colon and rectum, leading to a high risk of colon cancer. FAP is caused by mutations in the adenomatous polyposis coli (APC) gene, which regulates cell proliferation and differentiation. However, the molecular mechanisms underlying the development of FAP and the progression to colon cancer are not fully understood. In a recent study, iPSCs were generated from skin fibroblasts of FAP patients with different APC mutations and differentiated into intestinal organoids, which mimic the structure and function of the intestinal epithelium. The iPSC-derived organoids from FAP patients showed an increased number of polyps, aberrant Wnt signaling pathway activation, and increased proliferation compared to healthy control organoids. These results suggest that APC mutations lead to increased proliferation and aberrant Wnt signaling in the intestinal epithelium, contributing to the development of FAP and colon cancer. Furthermore, the FAP patient-derived organoids were sensitive to a Wnt inhibitor, demonstrating the potential of using iPSC-based disease models for drug discovery and personalized medicine [729].

### Impact on tumorigenesis understanding

Tumorigenesis is a complex process involving multiple genetic and epigenetic alterations that drive normal cells to become cancerous. Understanding the molecular mechanisms underlying tumorigenesis is critical for developing new cancer therapies and improving patient outcomes [530, 730]. iPSC-based disease modeling provides a powerful tool for studying tumorigenesis, as it allows for the generation of patient-specific disease models that recapitulate the tissue-specific characteristics of the cancer [730]. The iPSC-based disease models have been used to study the molecular changes associated with tumorigenesis, including changes in gene expression, epigenetic modifications, and protein signaling pathways [729]. For example, iPSC-based disease models have been used to study the genetic and epigenetic alterations associated with the development of breast cancer, leukemia, and glioblastoma [533, 549, 627]. These studies have provided valuable insights into the early events of cancer development and the underlying molecular mechanisms. In the context of tumorigenesis, iPSC-based disease models have helped uncover key genes and signaling pathways involved in tumor initiation and progression [543, 730]. By comparing iPSCs derived from healthy individuals and those with cancer, researchers have identified genetic mutations and gene expression changes specific to cancer cells. These findings have shed light on the dysregulation of important cellular processes, such as cell cycle control, DNA repair mechanisms, and cell signaling pathways, contributing to the understanding of how normal cells transform into cancer cells [24]. Furthermore, iPSC-based disease models have been instrumental in elucidating the role of epigenetic modifications in tumorigenesis. Epigenetic alterations, such as DNA methylation and histone modifications, can regulate gene expression patterns and contribute to the development and progression of cancer. iPSCs derived from cancer patients have allowed researchers to investigate epigenetic changes associated with specific cancer types and identify potential epigenetic markers for early cancer detection and targeted therapies [24]. Importantly, iPSC-based disease models have facilitated the study of tumor heterogeneity, a hallmark of cancer, which refers to the genetic and phenotypic diversity observed within tumors. By generating iPSCs from different regions or subpopulations of tumors, researchers have been able to recreate the heterogeneous nature of cancers in vitro [730]. This has enabled the exploration of tumor evolution, clonal dynamics, and the identification of subpopulations of cells responsible for metastasis or resistance to therapy [576]. Such knowledge is invaluable for developing personalized treatment strategies tailored to individual patients. Table 12 presents an overview of key case studies highlighting the application of iPSCs in understanding tumorigenesis.

**Table 12:** Key iPSC-based tumorigenesis case studies

Study Title	Key Results	Impact on Understanding of Tumorigenesis	References
“Induction of pluripotent stem cells from mouse embryonic and adult fibroblast cultures by defined factors” by Takahashi and Yamanaka, 2006	Demonstrated that adult cells could be reprogrammed to a pluripotent state, known as iPSCs, using only a few transcription factors	iPSCs have become a valuable tool for studying tumorigenesis as they can be differentiated into various cell types that can be used to model cancer progression and test potential therapies	[731]
“Modeling cancer using patient-derived induced pluripotent stem cells to understand development of childhood malignancies” by Navarro et al., 2018	Generated iPSCs from pediatric cancer patients to study the molecular mechanisms of cancer development	This study provided insight into the genetic and epigenetic changes that occur during cancer development and demonstrated the potential of iPSCs as a tool for personalized medicine	[15]
“Induced pluripotent stem cells as a tool for disease modelling and drug discovery in melanoma” by Castro-Pérez et al., 2019	Used iPSCs to model melanoma progression and test potential drug therapies	The study highlighted the potential of iPSCs as a platform for drug discovery and personalized medicine in cancer treatment	[732]
“Patient-derived induced pluripotent stem cells recapitulate hematopoietic abnormalities of juvenile myelomonocytic leukemia” by Kotini et al., 2017	Generated iPSCs from patients with juvenile myelomonocytic leukemia (JMML) and identified disease-specific abnormalities in hematopoietic differentiation	The study provided insight into the cellular mechanisms underlying JMML and demonstrated the potential of iPSCs for studying rare diseases	[733]
“Induced pluripotent stem cells from human kidney epithelial cells reprogrammed with OCT4/SOX2/NANOG” by Montserrat et al., 2013	Generated iPSCs from human kidney epithelial cells and identified genes associated with renal cell carcinoma (RCC)	The study provided insight into the genetic changes that occur during RCC development and identified potential therapeutic targets for the disease	[535]
“Induced pluripotent stem cells in Huntington's disease: A review” by Chia et al., 2020	Reviewed the use of iPSCs to model Huntington's disease (HD) and identified potential therapeutic strategies	The study highlighted the potential of iPSCs as a tool for studying neurodegenerative diseases like HD and testing potential therapies	[611]
“Modeling breast cancer using patient-derived induced pluripotent stem cells to study tumor heterogeneity” by Lefort et al., 2022	Generated iPSCs from breast cancer patients and observed the heterogeneity of tumor cells in vitro	This study provided valuable insights into the clonal evolution and heterogeneity of breast cancer, contributing to a better understanding of its progression and potential therapeutic targets	[532]
“Induced pluripotent stem cells as a model for studying the role of oncogenic mutations in lung cancer” by Chen et al., 2017	Introduced specific oncogenic mutations into iPSCs derived from lung cells and observed their effect on cellular behavior and tumorigenic potential	The study revealed the impact of specific oncogenic mutations in driving lung cancer development and provided a platform for screening targeted therapies	[593]
“Induced pluripotent stem cell-based modeling of glioblastoma multiforme” by Plummer et al., 2019	Reprogrammed iPSCs from glioblastoma multiforme (GBM) patients and differentiated them into neural cells to study GBM pathogenesis	The research provided insights into the molecular mechanisms underlying GBM development and allowed for the testing of potential targeted therapies	[125]
“Using induced pluripotent stem cells to study the role of chromosomal rearrangements in leukemia” by Chao et al., 2017	Generated iPSCs from leukemia patients with specific chromosomal rearrangements and investigated their impact on leukemogenesis	This study provided valuable information on the role of chromosomal rearrangements in leukemia development and helped identify potential therapeutic targets	[593]
“Modeling colorectal cancer using patient-derived induced pluripotent stem cells to study tumor initiation and progression” by Crespo et al., 2017	Generated iPSCs from colorectal cancer patients and observed the initiation and progression of tumors in a controlled environment	This study provided insights into the early events of colorectal cancer development, including tumor initiation and progression, and identified potential therapeutic targets	[556]
“Induced pluripotent stem cell-based modeling of prostate cancer to study tumor-stromal interactions” by Buskin et al., 2021	Reprogrammed iPSCs from prostate cancer patients and co-cultured them with stromal cells to investigate tumor-stromal interactions	The research shed light on the complex interplay between tumor cells and the surrounding stromal microenvironment in prostate cancer and identified potential therapeutic strategies	[267]
“Modeling pancreatic ductal adenocarcinoma using induced pluripotent stem cells to study tumor heterogeneity and therapeutic resistance” by Kim et al., 2013	Generated iPSCs from pancreatic ductal adenocarcinoma (PDAC) patients and characterized the heterogeneity of PDAC tumors and their responses to therapies	This study enhanced our understanding of the complex nature of PDAC and provided insights into the mechanisms underlying therapeutic resistance in this challenging cancer type	[554]
“Modeling glioma development using patient-derived induced pluripotent stem cells to study tumor metabolism” by Martinez et al., 2016	Generated iPSCs from glioma patients and analyzed alterations in tumor metabolism pathways	This study provided insights into the metabolic rewiring occurring in glioma development, offering potential targets for therapeutic intervention	[533]

## Personalized medicine and drug screening

Personalized medicine and drug screening are two interconnected concepts that have the potential to revolutionize healthcare [15]. Personalized medicine aims to tailor medical treatment to an individual's unique genetic makeup, lifestyle, and environmental factors [731]. Drug screening, on the other hand, involves testing a large number of compounds to identify potential drug candidates for a specific disease or condition [15]. Personalized medicine is based on the premise that every individual is unique and responds differently to medical treatments. The use of genomic sequencing, proteomic profiling, and other advanced technologies has made it possible to identify specific genetic mutations and biomarkers associated with different diseases [732]. By analyzing an individual's genetic makeup, physicians can identify which drugs are likely to be most effective and which ones may cause harmful side effects. One example of personalized medicine is the use of targeted therapies for cancer treatment [733]. Traditional chemotherapy drugs kill rapidly dividing cells, including healthy cells, leading to many negative side effects. Targeted therapies, however, are designed to specifically target cancer cells by exploiting their genetic vulnerabilities. For instance, some cancer cells have overactive growth receptors on their surface, which targeted therapies can block, slowing down cancer growth. Drug screening is a crucial step in the drug development process. It involves testing thousands of compounds to identify those that have the potential to become effective drugs for a specific disease or condition [611]. High-throughput screening (HTS) is a popular approach that allows researchers to rapidly test a large number of compounds. HTS can be used to identify new drugs, repurpose existing drugs for new uses, or optimize existing drugs to improve their efficacy [731]. Advances in drug screening technologies, such as computer simulations and artificial intelligence (AI), have significantly accelerated the drug discovery process. These technologies can predict how a drug will interact with a specific target, predict toxicity, and identify potential drug combinations [732]. By using these tools, researchers can quickly identify potential drug candidates and prioritize those with the greatest potential for success. Personalized medicine and drug screening are becoming increasingly intertwined [15]. By analyzing an individual's genetic makeup, physicians can identify which drugs are likely to be most effective for a particular patient. This can significantly reduce the trial-and-error process associated with traditional medicine [732]. Drug screening can also be personalized by testing drugs on patient-derived cells or tissue samples. This approach can help predict which drugs are most likely to be effective for a particular patient, and avoid those that are likely to cause harm [733]. One example of the integration of personalized medicine and drug screening is the development of immunotherapies for cancer treatment [731]. Immunotherapies work by stimulating the body's immune system to target and kill cancer cells. However, not all patients respond equally to immunotherapies [733]. By analyzing an individual's genetic makeup, researchers can identify which patients are most likely to respond to a particular immunotherapy and which ones are not. This can help tailor treatment to each patient and improve the overall success rate of immunotherapies. Personalized medicine and drug screening are two important concepts that have the potential to transform healthcare [15]. Personalized medicine aims to tailor medical treatment to an individual's unique genetic makeup, lifestyle, and environmental factors, while drug screening involves testing a large number of compounds to identify potential drug candidates for a specific disease or condition [732]. The integration of these two concepts can significantly improve patient outcomes by reducing trial and error and tailoring treatment to each individual's needs. With advances in technology and research, personalized medicine and drug screening are poised to become the standard of care in many areas of healthcare [733].

## Regenerative medicine and immunotherapies

**Fig. 14:**
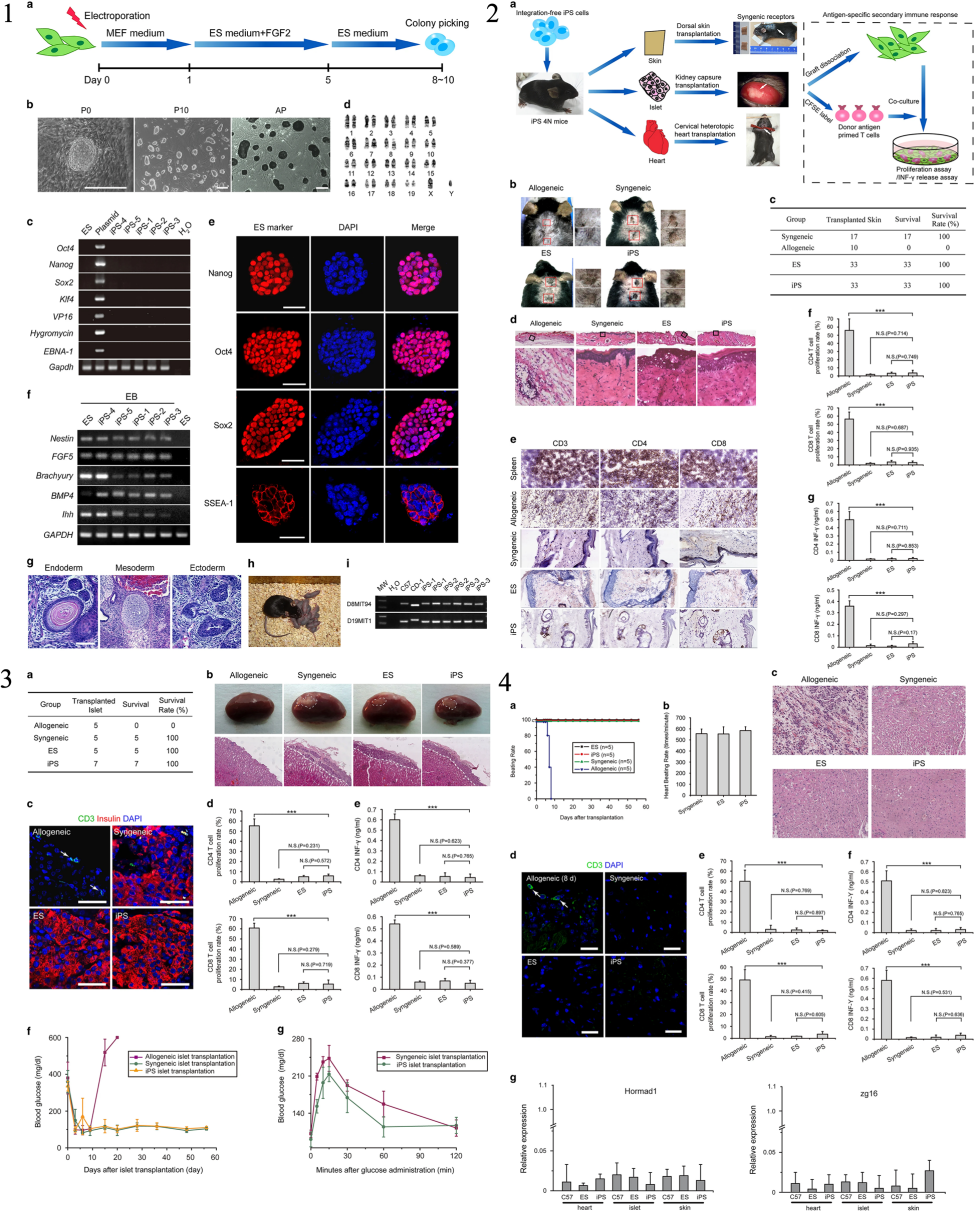
**1** The process of creating 4n complementation mice using iPSCs without the need for integration. Panel (a) shows the method used to generate integration-free iPSCs. Panel (b) displays the morphology and alkaline phosphatase staining of these iPSCs. Panel (c) presents PCR analysis results, indicating the absence of integration of the reprogramming vector in the iPSC lines tested. The reprogramming plasmid serves as a positive control. Panel (d) demonstrates the normal karyotypes of the iPSCs through G-banding chromosomal analysis. Panel (e) exhibits immunofluorescence staining of pluripotent markers (Nanog, Oct3/4, Sox2, and SSEA-1) in iPSCs. Panel (f) displays the results of RT-PCR analysis, indicating successful differentiation of iPSCs into three germ layers. Panel (g) shows the formation of teratomas containing all three embryonic germ layers when iPSCs are injected into severe-combined-immune-deficiency mice. Panel (h) represents iPSC mice generated through 4n complementation. Finally, panel (i) presents the results of SSLP analysis, which distinguishes mice derived from different iPSC lines. **2** The successful transplantation of skins derived from iPSCs, which were well-tolerated by the host and effectively repaired skin wounds. In (a), a schematic diagram demonstrates the transplantation of skin, islets, and hearts derived from iPSC mice onto different locations of recipient mice. T-cell proliferation or interferon (INF)-γ release assays were used to detect primed T cells. (b) showcases the wound repair achieved through the transplantation of iPSC-derived skin. The transplanted iPSC skin, similar to embryonic stem cell (ESm) and genetically identical skin (syngeneic), survived successfully for over 100 days in recipient mice. Allogeneic skin transplants, serving as negative controls, were rejected within three weeks. Representative images in (b) depict the grafts 20 weeks after transplantation. (c) provides a summary of the survival rates of explanted iPSC skin 20 weeks post-transplantation. ESm and syngeneic skin transplants are shown as positive controls, while allogeneic skin transplants are negative controls. (d) displays histological staining (H&E staining) of iPSC skin isolated from recipient mice eight weeks after transplantation. Allografts were stained one week after transplantation and served as a negative control. iPSC skin explants exhibited normal structures similar to ESm and syngeneic mice, while extensive tissue necrosis was observed in allografts. (e) demonstrates that T-cell infiltration was minimal in iPSC skin explants eight weeks after transplantation. T cells were identified through immunostaining using anti-CD3, anti-CD4, and anti-CD8 antibodies. Sections from the spleen and allogeneic skin grafts (one week after transplantation) were used as positive controls. (f) quantifies the percentage of proliferating cells, while (g) presents an interferon (IFN)-γ release assay to detect primed T cells in recipients of iPSC-derived skin. The quantified results are shown as mean ± s.e.m. of triplicates for each group (syngeneic: *n* = 3; ESm: *n* = 6; iPSC: *n* = 6; allogeneic: *n* = 3). **3** The effectiveness of iPSC-derived islets in reducing high glucose levels in diabetic mice. In panel (a), the survival of iPSm islets in C57BL/6 hosts is summarized after 8 weeks of transplantation. Panel (b) displays representative images of iPSm islets that were transplanted under kidney capsules, with dot circles indicating the location of the grafted islets. Panel (c) shows the detection of T-cell infiltration in iPSm islets using an anti-CD3 antibody (shown in green). Engrafted islets are labeled with anti-insulin staining (shown in red). In panel (d), the quantification of T-cell proliferation induced by different stimulators is presented, with the mean and standard error of the mean (s.e.m.) shown for each group (syngeneic, ESm, iPSm, and allogeneic). Panel (e) presents the quantification of interferon (IFN)-γ release, again with the mean and s.e.m. shown for each group (syngeneic, ESm, iPSm, and allogeneic). Panel (f) displays the monitoring of blood glucose levels in diabetic mice that were engrafted with allogeneic, syngeneic, and iPSm islets. The different groups are represented by different colors (iPSm in yellow, syngeneic in green, and allogeneic in purple). Finally, in panel (g), the glucose tolerance test conducted 8 weeks after islet transplantation is shown. Diabetic mice engrafted with iPSm islets (represented in green) exhibited efficient response to high-glucose injection similar to mice transplanted with syngeneic islets (represented in purple). **4** Heart transplantation using iPSC-derived cells. (a) Survival rates of mouse hearts derived from iPSCs (iPSm), embryonic stem cells (ESm), syngeneic (genetically identical), and allogeneic (genetically different) transplants in recipient mice. (b) iPSm hearts beat at similar rates to ESm and syngeneic hearts. (c) Transplanted hearts were examined using H&E staining. (d) T-cell infiltration was assessed by staining heart sections with anti-CD3 antibodies (green). No significant T-cell infiltration was observed in iPSm, ESm, and syngeneic mouse hearts from genetically identical recipients, while allografts showed extensive T-cell infiltration (positive controls). Scale bars represent 50 μm. (e) T-cell proliferation and (f) interferon (INF)-γ release were measured to detect activated T cells in mice with iPSm, ESm, syngeneic, and allogeneic heart transplants. (g) Expression of the Zg16 and Hormad1 genes in transplanted skin, islets, and hearts eight weeks after transplantation. Reprinted from [734] with permission from the Springer Nature

Regenerative medicine and immunotherapies represent groundbreaking approaches in the field of healthcare, offering promising solutions for the treatment of various diseases, including cancer [555, 606]. Regenerative medicine aims to restore, replace, or regenerate damaged tissues and organs, while immunotherapies harness the power of the immune system to combat diseases. These two fields have gained significant attention and are revolutionizing the landscape of modern medicine [731]. Regenerative medicine focuses on harnessing the regenerative potential of stem cells, including iPSCs, to repair and replace damaged tissues and organs [732]. iPSCs are generated by reprogramming adult cells, such as skin cells, into a pluripotent state, giving them the ability to differentiate into various cell types. This remarkable feature of iPSCs makes them an attractive tool for regenerative medicine applications [611]. They can be differentiated into specific cell lineages, such as cardiomyocytes for heart repair or pancreatic beta cells for diabetes treatment [15]. One of the key advantages of regenerative medicine is its potential to provide personalized therapies. By utilizing a patient's own cells to generate iPSCs, it becomes possible to create tissue or organ grafts that are genetically identical to the patient, reducing the risk of immune rejection and the need for immunosuppressive drugs. This personalized approach holds great promise for improving patient outcomes and minimizing complications associated with traditional transplantation methods. In the realm of cancer treatment, regenerative medicine offers innovative strategies. iPSCs can be used to model cancer in a laboratory setting, providing researchers with a platform to study the mechanisms of tumor formation, progression, and response to various treatments. This enables the development of more effective and targeted therapies [555, 606]. Furthermore, iPSCs can be engineered to express therapeutic genes or anti-cancer agents, acting as “cellular factories” that produce and release these substances specifically at the site of the tumor. Immunotherapies, on the other hand, harness the body's immune system to recognize and eliminate cancer cells. Traditional cancer treatments, such as chemotherapy and radiation therapy, often have systemic side effects and can harm healthy tissues [15]. Immunotherapies offer a more targeted approach, aiming to enhance the immune response against cancer cells while minimizing damage to normal cells [732]. One of the most exciting developments in immunotherapy is the use of immune checkpoint inhibitors [611]. These inhibitors block the proteins that prevent immune cells from recognizing and attacking cancer cells. By releasing these immune checkpoints, the body's immune system is reactivated and can mount a robust response against the tumor. This approach has shown remarkable success in treating various cancers, including melanoma, lung cancer, and bladder cancer [555, 606]. Another promising immunotherapy strategy is adoptive cell transfer, which involves isolating immune cells, such as T cells, from a patient, genetically modifying them to express receptors that recognize specific cancer antigens, and then reintroducing these cells back into the patient's body [732]. These modified immune cells can then specifically target and eliminate cancer cells, leading to tumor regression. This approach has shown remarkable efficacy in the treatment of hematological malignancies, such as leukemia and lymphoma. Combining regenerative medicine with immunotherapies can offer even greater therapeutic potential [555, 606]. iPSCs can be genetically engineered to express immune-stimulatory molecules, such as cytokines, which can enhance the immune response against tumors [15]. Furthermore, iPSCs can be differentiated into immune cells, such as dendritic cells or natural killer cells, which can be used in cancer immunotherapies to enhance the anti-tumor immune response. Regenerative medicine and immunotherapies are revolutionizing the field of healthcare, particularly in the realm of cancer treatment. The regenerative potential of iPSCs opens up new avenues for tissue repair and personalized therapies [611]. Meanwhile, immunotherapies are providing targeted and effective approaches to activate the immune system against cancer cells. Combining these two fields can lead to even more effective and personalized treatments for patients [731]. However, as with any emerging technology, there are also challenges and ethical considerations that need to be addressed. One of the key challenges is the safety and efficacy of these therapies [732]. While regenerative medicine and immunotherapies hold great promise, more research is needed to fully understand their mechanisms and potential risks. There is also a need for standardized protocols for the manufacturing, testing, and administration of these therapies to ensure their safety and efficacy. Another consideration is the cost and accessibility of these therapies [611]. Regenerative medicine and immunotherapies can be expensive, and there is a need to ensure that they are accessible to all patients who could benefit from them. This includes developing more affordable and scalable manufacturing methods for these therapies [15]. There are also ethical considerations related to the use of human embryonic stem cells and iPSCs. While the use of iPSCs avoids the ethical concerns associated with the use of embryonic stem cells, there are still concerns regarding the consent and privacy of patients who donate their cells for research [555, 606]. There is a need for clear guidelines and ethical frameworks to ensure that the use of iPSCs is conducted in an ethical and responsible manner. Despite these challenges, regenerative medicine and immunotherapies represent a promising future for healthcare [611]. By harnessing the regenerative potential of stem cells and the power of the immune system, these therapies offer innovative solutions for the treatment of various diseases, including cancer. With continued research and development, regenerative medicine and immunotherapies have the potential to transform the way we approach healthcare and provide personalized and effective treatments for patients. In addition to their potential in cancer treatment, regenerative medicine and immunotherapies hold promise in a wide range of other medical conditions [732]. For example, in the field of tissue engineering, regenerative medicine approaches aim to create functional tissues and organs that can replace damaged or diseased ones. This can be particularly beneficial for patients with organ failure or those in need of organ transplantation. By utilizing stem cells and bioengineering techniques, researchers are making strides in developing functional tissues such as heart muscle, liver tissue, and even entire organs like kidneys [15]. Furthermore, regenerative medicine approaches are being explored for the treatment of degenerative diseases such as Parkinson's and Alzheimer's. The ability to generate specific cell types from stem cells opens up possibilities for cell replacement therapies. Scientists are investigating the use of iPSCs to generate neurons that can be transplanted into the brains of patients with neurodegenerative disorders, potentially restoring lost function and improving quality of life [732]. Immunotherapies, on the other hand, have shown promise in various other areas beyond cancer treatment [611]. For instance, they are being investigated for the treatment of autoimmune diseases, where the immune system mistakenly attacks healthy cells and tissues. By modulating the immune response, immunotherapies offer a targeted approach to suppress or regulate the immune system, reducing inflammation and preventing damage to the body's own tissues. Moreover, immunotherapies have demonstrated potential in infectious diseases, such as HIV and viral hepatitis [555, 606]. Researchers are exploring strategies to boost the immune system's ability to recognize and eliminate viral pathogens, offering new avenues for the development of antiviral therapies [611]. In the field of transplantation, immunotherapies are being investigated to improve the success of organ and tissue transplantation. By modulating the immune response and preventing organ rejection, these therapies have the potential to increase the availability of donor organs and improve patient outcomes [732]. While regenerative medicine and immunotherapies are still relatively new fields, the progress and advancements made so far are highly promising [733]. Ongoing research and clinical trials are expanding our understanding of these approaches and paving the way for their integration into mainstream medical practice [611]. Regenerative medicine and immunotherapies represent transformative approaches in healthcare, with the potential to revolutionize the treatment of various diseases. Regenerative medicine offers the possibility of tissue and organ regeneration, personalized therapies, and disease modeling using iPSCs. Immunotherapies harness the power of the immune system to target and eliminate cancer cells, as well as to treat autoimmune disorders and infectious diseases [555, 606]. In a recent study conducted by Wang et al., the researchers investigated the immunogenicity and functional evaluation of iPSC-derived organs for transplantation [734]. The study aimed to determine whether iPSC-derived organs, including skin, islet, and heart tissues, were capable of surviving, repairing tissue damage, and functioning effectively after transplantation. The researchers utilized 4n complementation to generate iPSC-derived organs without integration. Figure 14 highlights the process of creating 4n complementation mice using iPSCs without integration, showcasing the innovative approach employed in this study. Figure 14 demonstrates the successful transplantation of iPSC-derived skin, which not only survived but also effectively repaired skin wounds in recipient mice. This finding indicates the potential therapeutic application of iPSC-derived skin for tissue regeneration. Figure 14 further illustrates the positive effects of iPSC-derived islets in diabetic mouse models. The transplanted iPSC-derived islets successfully produced insulin and effectively reduced high glucose levels, suggesting their therapeutic potential for treating diabetes. Importantly, Fig. 14 also highlights the limited immunogenicity of iPSC-derived islets, indicating their compatibility for transplantation without eliciting significant immune rejection responses. In Fig. 14, the outcomes of heart transplantation using iPSC-derived hearts are depicted. The iPSC-derived heart grafts displayed normal beating for a duration of over 3 months in syngeneic recipients, demonstrating their functional viability and long-term survival. This finding provides promising evidence for the feasibility of using iPSC-derived hearts in transplantation procedures. In a recent investigation conducted by Wang and colleagues, the researchers explored the immune response and functional assessment of organs derived from iPSCs intended for transplantation [734]. The study aimed to ascertain whether iPSC-derived organs, such as skin, islet, and heart tissues, could endure, repair damaged tissue, and operate effectively post-transplantation. To create iPSC-derived organs devoid of integration, the researchers employed a 4n complementation approach. Figure 14 illustrates the process of generating 4n complementation mice using iPSCs without integration, showcasing the innovative method used. Figure 14 showcases the successful transplantation of iPSC-derived skin, which not only survived but also efficiently healed skin injuries in recipient mice, suggesting the potential therapeutic utility of iPSC-derived skin for tissue regeneration. Figure 14 further illustrates the favorable effects of iPSC-derived islets in diabetic mouse models. These transplanted islets produced insulin and effectively lowered elevated glucose levels, indicating their potential for treating diabetes. Additionally, Fig. 14 highlights the limited immunogenicity of iPSC-derived islets, suggesting their compatibility for transplantation without significant immune rejection. In Fig. 14, the results of heart transplantation using iPSC-derived hearts are depicted. The iPSC-derived heart grafts exhibited normal beating for over 3 months in syngeneic recipients, demonstrating their functional viability and long-term survival. This discovery offers promising evidence for the practicality of utilizing iPSC-derived hearts in transplantation procedures.

## Ethical and legal considerations

In recent years, the iPSCs have emerged as a powerful tool in cancer research and therapy [41, 735]. However, their use raises important ethical and legal considerations that need to be carefully addressed. This section will discuss four key aspects: the ethics of using human cells in iPSC research, intellectual property rights, regulation of iPSC research, and the need for international consensus on ethical and legal issues [15, 736]. The utilization of human cells, including iPSCs, raises ethical questions related to the source of cells, their derivation, and potential risks [45, 737]. iPSCs are typically generated from adult somatic cells, such as skin or blood cells, which are reprogrammed to exhibit pluripotent properties [41, 738]. While these cells offer great potential for advancing cancer research, their use necessitates ethical considerations. Researchers must ensure that individuals providing cells for iPSC research are adequately informed about the purpose, risks, and potential benefits of the research [38, 739]. Respect for donor autonomy and privacy should be maintained throughout the process, including secure data management and confidentiality. Another ethical consideration is the use of embryos in iPSC research. Initially, iPSCs were generated by reprogramming human embryos, which raised ethical concerns due to the destruction of embryos [41, 740]. However, advancements in reprogramming techniques now allow for the generation of iPSCs from adult cells, avoiding the need for embryo destruction. This approach alleviates ethical concerns associated with embryo use [15, 741]. Intellectual property rights play a significant role in iPSC research, as they can impact the accessibility and affordability of iPSC-based cancer therapies. Patents are often filed for specific techniques, methods, or applications involving iPSCs [45]. These patents grant exclusive rights to the patent holder, potentially limiting the development of alternative therapies. The issue of patenting iPSC technology raises concerns about equitable access to treatments and the sharing of scientific knowledge [41, 742]. Balancing the interests of patent holders with the broader goal of advancing cancer research and treatment is crucial [15]. Encouraging collaborations, licensing agreements, and patent pools can foster innovation while ensuring that iPSC technology is accessible to all researchers and clinicians. Regulation is an essential aspect of iPSC research to ensure the safety and ethical conduct of experiments [45, 743]. Regulatory frameworks differ among countries, and variations in regulations can impact the pace of progress in iPSC-based cancer research. Stringent regulations may slow down research by imposing lengthy approval processes and stringent safety requirements [41, 744]. While necessary to protect participants and patients, these regulations should be designed to strike a balance between safety and facilitating scientific advancements. Harmonizing regulatory standards across countries can promote collaboration, knowledge sharing, and the efficient translation of iPSC-based therapies from the lab to the clinic. Given the global nature of scientific research and the potential impact of iPSCs on tumorigenesis and therapy, there is a pressing need for international consensus on ethical and legal issues [15]. Establishing guidelines and standards can ensure uniformity in research practices and promote responsible and ethical use of iPSCs [38, 745]. International consensus can address several aspects, including informed consent procedures, data sharing, privacy protection, and research collaborations [45, 746]. By facilitating dialogue and agreement among researchers, clinicians, ethicists, and policymakers from different countries, international consensus can help navigate the ethical complexities associated with iPSC research [41, 747]. Collaborative efforts such as the International Society for Stem Cell Research (ISSCR) and national regulatory bodies play a vital role in fostering consensus and developing guidelines. These initiatives promote transparency, encourage ethical conduct, and address the legal challenges surrounding iPSC research [45, 748]. Through international collaboration, stakeholders can share best practices, exchange knowledge, and establish common ethical standards. This can enhance the credibility and reliability of iPSC-based cancer research and ensure that the potential benefits are maximized while minimizing potential risks [41, 749]. Moreover, international consensus on intellectual property rights can facilitate the fair and equitable distribution of iPSC technologies. It can encourage licensing agreements that allow for broader access to iPSC-related discoveries, thereby promoting innovation and accelerating progress in cancer research [15, 750]. Addressing ethical and legal considerations surrounding iPSC research requires interdisciplinary engagement. Collaboration between scientists, clinicians, ethicists, legal experts, policymakers, and patient advocacy groups is crucial to develop comprehensive guidelines that navigate the complex ethical landscape [45, 751]. Furthermore, public engagement and dialogue are vital to ensure that societal values and concerns are taken into account. Including diverse perspectives and involving the public in discussions related to iPSC research can foster transparency, trust, and support for scientific endeavors [38, 752]. As with any emerging technology, safety is a paramount concern in iPSC research. It is essential to thoroughly assess the potential risks associated with the use of iPSCs, such as tumorigenicity, genetic instability, and immunogenicity [15, 753–758]. Robust preclinical studies and careful monitoring of patients participating in clinical trials are crucial to ensure the safety and efficacy of iPSC-based therapies. Respecting patient autonomy and ensuring informed consent are central to conducting ethical iPSC research. Patients must have a clear understanding of the nature of the research, potential benefits and risks, and their rights to withdraw from participation at any time [41, 759]. Informed consent processes should be transparent, comprehensive, and culturally sensitive, taking into account the unique challenges and complexities of iPSC research [45, 760]. The development of iPSC-based therapies has the potential to revolutionize cancer treatment, but it is important to ensure that the benefits are accessible and affordable for all patients [38, 761–763]. Addressing issues of affordability, equitable distribution, and fair pricing can help mitigate disparities in access to these advanced therapies [38, 764–768]. Collaboration between researchers, industry, and policymakers is vital in developing strategies to make iPSC-based treatments accessible to diverse populations. Public perception and understanding of iPSC research can greatly influence its acceptance and support. Public education initiatives aimed at increasing awareness and knowledge about iPSCs, their potential applications, and the ethical considerations involved are essential [41, 769]. Open dialogue between scientists and the public can foster trust, address concerns, and ensure that societal values are reflected in the development and implementation of iPSC-based therapies. As iPSC-based therapies move from research settings to clinical applications, long-term monitoring and follow-up of patients are crucial [15, 770]. This is necessary to assess the long-term safety, effectiveness, and potential side effects of iPSC-based treatments [38, 771]. Establishing comprehensive surveillance programs and patient registries can provide valuable data for ongoing evaluation and refinement of iPSC therapies [45, 772]. Effective ethical oversight and governance mechanisms are essential to ensure the responsible conduct of iPSC research. Regulatory bodies, research institutions, and ethics committees play a vital role in reviewing and approving research protocols, monitoring compliance with ethical guidelines, and addressing any ethical concerns that may arise [38, 773]. Table 13 highlights the key ethical and legal considerations associated with iPSC research. These considerations play a crucial role in shaping the ethical framework and legal regulations surrounding the field. They encompass various aspects such as informed consent, privacy protection, research involving human subjects, intellectual property rights, and potential misuse of iPSC technology.

**Table 13:** Key ethical and legal considerations in iPSC research

Consideration	Description	References
Intellectual Property Rights	Refers to the legal ownership and control over the intellectual property (IP) generated from iPSC research. This consideration involves issues such as patenting iPSC technologies, ownership of cell lines, licensing agreements, and potential conflicts over IP rights between researchers, institutions, and commercial entities	[733, 774]
Regulations	Refers to the regulatory frameworks and guidelines that govern iPSC research. This consideration involves compliance with applicable laws, regulations, and ethical guidelines at the national, regional, and institutional levels. It includes obtaining appropriate research approvals, informed consent from donors of biological materials, and adherence to ethical standards in research involving human subjects	[535, 775]
International Consensus	Refers to the need for a global agreement or consensus on ethical and legal standards in iPSC research. This consideration involves addressing differences in regulations and ethical frameworks across countries and fostering collaboration and sharing of data, resources, and knowledge while respecting cultural, legal, and social diversity	[611]
Privacy and Confidentiality	Refers to protecting the privacy and confidentiality of individuals who contribute biological materials for iPSC research. This consideration involves implementing measures to ensure that personal information and data are handled securely, anonymized when necessary, and used only for authorized purposes while complying with applicable privacy laws and regulations	[532]
Informed Consent	Refers to obtaining voluntary, informed, and documented consent from individuals who provide biological materials for iPSC research. This consideration involves ensuring that potential donors are adequately informed about the nature, purpose, risks, and benefits of the research and that their consent is obtained without coercion or undue influence	[593]
Ethical Use of iPSCs in Research	Refers to conducting iPSC research in an ethically responsible manner. This consideration involves adhering to ethical principles, such as respect for autonomy, beneficence, non-maleficence, and justice. It includes considering the potential ethical implications of iPSC research, such as the creation and destruction of embryos, the use of human-animal chimeras, and potential social implications	[38]
Equity and Access to iPSC Technologies	Refers to ensuring equitable access to iPSC technologies, benefits, and potential therapies. This consideration involves addressing issues of fairness, affordability, and accessibility, particularly in the context of healthcare disparities and global health challenges. It includes promoting the inclusion of diverse populations and addressing barriers to access for underserved communities	[15, 776]
Data Sharing and Collaboration	Refers to the sharing of research data, resources, and knowledge among researchers and institutions involved in iPSC research. This consideration involves promoting open science principles, facilitating data sharing while respecting privacy and confidentiality, and fostering collaboration to accelerate scientific progress and maximize the benefits of iPSC research	[45]
Ethical and Responsible Conduct of Research	Refers to upholding high ethical standards and responsible conduct in all aspects of iPSC research. This consideration involves ensuring integrity, transparency, and accountability in research practices, including study design, data collection, analysis, publication, and dissemination. It includes promoting research integrity and addressing conflicts of interest or misconduct	[46]
Genetic Privacy and Discrimination	Refers to protecting the privacy of an individual's genetic information obtained through iPSC research and preventing potential discrimination based on genetic data. This consideration involves implementing safeguards to ensure that genetic information is not misused, disclosed without consent, or used to discriminate against individuals in areas such as employment, insurance, or social services	[47]
Research on Vulnerable Populations	Refers to conducting iPSC research involving vulnerable populations, such as minors, individuals with cognitive impairments, or individuals lacking decision-making capacity. This consideration involves implementing additional safeguards to protect the rights and welfare of these individuals, including obtaining informed consent from legally authorized representatives and ensuring the research benefits outweigh the potential risks	[48]
Benefit-Sharing and Return of Results	Refers to addressing the equitable sharing of benefits and returning research results to participants or communities involved in iPSC research. This consideration involves establishing mechanisms and policies to ensure that the benefits derived from iPSC research, such as potential therapies or commercial products, are shared fairly and that research findings are communicated back to participants in an understandable manner	[49]
Animal Welfare in iPSC Research	Refers to considering and minimizing potential harm or suffering experienced by animals used in iPSC research, particularly in experiments involving human-animal chimeras. This consideration involves following ethical guidelines for animal welfare, implementing appropriate animal care and use protocols, and exploring alternative methods to reduce or replace animal models when feasible	[50]
Research Transparency and Reproducibility	Refers to promoting transparency and reproducibility in iPSC research. This consideration involves sharing research protocols, methods, and data openly, making research findings accessible for scrutiny and replication, and adhering to best practices in research design, statistical analysis, and reporting to ensure the reliability and validity of scientific findings	[51]
Commercialization and Accessibility	Refers to the balance between commercial interests and ensuring accessibility of iPSC technologies and therapies. This consideration involves addressing issues related to affordability, affordability, and fair pricing of iPSC-based products, as well as implementing measures to ensure that essential iPSC research tools and technologies are widely available for scientific advancement	[52]
Governance and Oversight	Refers to establishing appropriate governance and oversight mechanisms for iPSC research. This consideration involves defining responsible conduct guidelines, establishing research ethics committees or institutional review boards, and monitoring compliance with ethical and legal standards to ensure the responsible and accountable conduct of iPSC research	[53]
Social and Cultural Considerations	Refers to recognizing and addressing social and cultural factors in iPSC research. This consideration involves engaging with diverse stakeholders, including communities affected by the research, to understand and address potential cultural, social, or value-based concerns. It includes respecting cultural practices, beliefs, and societal norms in the design, implementation, and dissemination of iPSC research	[45]

## Unveiling the potential and challenges of iPSCs in cancer initiation research

In the field of cancer research, gaining a deep understanding of the complexities involved in the initiation of cancer is a crucial and top-priority objective [15]. One groundbreaking approach that has significantly transformed our methods for deciphering this mysterious process is the application of iPSCs. These extraordinary cellular entities provide a distinct advantage in our quest to unravel the secrets surrounding the onset of cancer, primarily because of their remarkable adaptability [45]. iPSCs possess the exceptional capacity to transform into the very cells from which various types of cancer originate. This unique attribute empowers researchers to explore the molecular and cellular mechanisms underpinning cancer initiation in ways that were previously considered unimaginable [15]. Nevertheless, it is of utmost importance for authors embarking on research in this field to address a pivotal query: What is the actual contribution of iPSCs to our comprehension of cancer initiation? This inquiry requires a thorough examination of the potential advantages and constraints inherent in investigations centered on iPSCs [45]. By engaging in this exploration, scientists can provide invaluable insights into the intricate landscape of cancer initiation. It is through this scrutiny that the genuine worth of iPSCs within the context of cancer research becomes evident. By elucidating the intricate interplay between genetic and environmental factors in the formation of cancerous cells, iPSCs offer a platform for examining the earliest phases of carcinogenesis, potentially paving the path for innovative therapeutic interventions and early detection methods [41]. Nonetheless, it is crucial to acknowledge the limitations associated with the use of iPSCs in unraveling the puzzle of cancer initiation. These limitations encompass challenges related to accurately replicating the microenvironment and epigenetic modifications occurring during the natural progression of cancer [45]. Furthermore, the inherent variability among different iPSC lines, in conjunction with the complexity of modeling various cancer types, emphasizes the need for cautious and meticulous experimental design [41]. While iPSCs present an unparalleled opportunity to shed light on cancer initiation, researchers must navigate a multifaceted terrain marked by subtleties and restrictions to effectively harness their full potential. By addressing these concerns, the scientific community can chart a more precise course towards harnessing the capabilities of iPSCs to illuminate the intricate aspects of cancer initiation [45].

## Conclusion

The iPSCs have revolutionized the field of stem cell research and have shown tremendous potential in the development of new cancer therapies. In recent years, significant progress has been made in iPSC-based tumorigenesis research. Researchers have been able to generate iPSCs from cancer cells, providing a unique model for studying the molecular changes that occur during cancer development and progression. The iPSCs have also been used to develop personalized cancer therapies, allowing for targeted treatments based on a patient's specific genetic and epigenetic profiles. In addition, iPSCs have been used for drug screening, allowing for the identification of new compounds that may be effective in treating various types of cancer. Furthermore, iPSCs have been used to generate immune cells that can be used in immunotherapies, which have shown great promise in the treatment of certain types of cancer. iPSCs have also been used to develop cancer early detection methods, allowing for earlier diagnosis and treatment of cancer. The impact of iPSCs on the future of cancer research and treatment cannot be overstated. iPSCs offer a powerful tool for studying the molecular mechanisms of tumorigenesis and for developing new cancer therapies. The ability to generate iPSCs from cancer cells allows for the study of individual patient's tumors, leading to personalized treatments that may be more effective and have fewer side effects than current treatments. The iPSCs have also been used to develop immunotherapies, which have shown great promise in the treatment of certain types of cancer. Immunotherapies work by harnessing the power of the patient's own immune system to attack cancer cells. iPSCs can be used to generate immune cells that can be used in these therapies, providing a potential source of unlimited immune cells for cancer treatment. Furthermore, iPSCs offer a unique platform for drug screening and the development of new cancer treatments. By using iPSCs to generate different types of cells, researchers can test the efficacy and safety of potential new cancer drugs before testing them in animal models or human clinical trials. While iPSCs hold great promise in the field of cancer research and treatment, there are still many challenges that need to be overcome. One of the major challenges is the tumorigenic properties of iPSCs, which can lead to the formation of teratomas or other types of tumors. Additionally, the efficiency of iPSC generation needs to be improved, and the safety and efficacy of iPSC-based therapies need to be thoroughly evaluated. Continued investment in iPSC research is crucial to unlocking the full potential of these cells. Funding for iPSC research will allow for the development of new technologies and methods for generating iPSCs and for evaluating their safety and efficacy. In addition, continued investment in iPSC research will enable the development of new cancer therapies and the optimization of existing therapies. The iPSCs offer a powerful tool for studying tumorigenesis and for developing new cancer therapies. While significant progress has been made in iPSC-based tumorigenesis research, there are still many challenges that need to be overcome. Continued investment in iPSC research is crucial to unlocking the full potential of these cells and to realizing their promise in the field of cancer research and treatment. Some recommendations for future research include developing new methods for generating iPSCs that are safer and more efficient, improving the safety and efficacy of iPSC-based therapies, and developing new immunotherapies that utilize iPSC-generated immune cells. In addition, further research should focus on understanding the molecular changes that occur during cancer development and progression using iPSCs as a model. This will provide valuable insights into the mechanisms underlying tumorigenesis and help identify potential targets for therapeutic interventions. Moreover, it is important to explore the potential of iPSCs in combination with other treatment modalities. Combining iPSC-based therapies with existing cancer treatments, such as chemotherapy or radiation therapy, may enhance their effectiveness and improve patient outcomes. Additionally, investigating the synergistic effects of iPSC-derived immune cells with other immunotherapeutic approaches could lead to more robust and durable anti-cancer responses. Ethical and legal considerations surrounding iPSC research should also be addressed. As iPSCs can be derived from a patient's own cells, there are fewer ethical concerns compared to other types of stem cells. However, careful regulation and guidelines should be in place to ensure responsible and ethical use of iPSCs in research and clinical applications. In conclusion, iPSCs have demonstrated remarkable potential in the field of cancer research and therapy. The advancements made in iPSC-based tumorigenesis research have shed light on the complex processes involved in cancer development and have opened up new avenues for personalized medicine and innovative treatment strategies. However, there is still much work to be done to fully unlock the potential of iPSCs. Continued investment in iPSC research, both in terms of funding and collaboration between researchers and clinicians, is essential. This will enable further advancements in iPSC generation techniques, enhance our understanding of cancer biology, and facilitate the translation of iPSC-based therapies into clinical practice. With concerted efforts and ongoing research, iPSCs have the potential to revolutionize cancer treatment, improve patient outcomes, and ultimately contribute to the goal of eradicating cancer.

## Data Availability

Not applicable.
